# Supplementary material for: Error-independent effect of sensory uncertainty on motor learning when both feedforward and feedback control processes are engaged
Source: PLoS Comput Biol. 2023 Sep 8;19(9):e1010526. doi: 10.1371/journal.pcbi.1010526 (PMC10522034; doi:10.1371/journal.pcbi.1010526)
Supplement: S1 Table — Abbreviations are std is standard deviation, T is the t-statistic, dof is degrees of freedom, p-corr is the p-value corrected for multiple comparisons, and hedges is Hedges g. (PDF) [file pcbi.1010526.s001.pdf]

row	A	B	mean(A)	std(A)	mean(B)	std(B)	T	dof	p-corr	hedges
0	Bias-scaling	Error-scaling	-366.45	12.70	-343.82	12.23	-17.70	19.00	1.00	-1.78
1	Bias-scaling	Retention-scaling	-366.45	12.70	-324.86	12.48	-24.11	19.00	1.00	-3.24
2	Bias-scaling	State-aim-scaling	-366.45	12.70	-380.96	12.64	21.56	19.00	0.00	1.12
3	Bias-scaling	Output-aim-scaling	-366.45	12.70	-380.96	12.63	21.76	19.00	0.00	1.12
4	Error-scaling	Retention-scaling	-343.82	12.23	-324.86	12.48	-17.16	19.00	1.00	-1.50
5	Error-scaling	State-aim-scaling	-343.82	12.23	-380.96	12.64	30.91	19.00	0.00	2.93
6	Error-scaling	Output-aim-scaling	-343.82	12.23	-380.96	12.63	30.92	19.00	0.00	2.93
7	Retention-scaling	State-aim-scaling	-324.86	12.48	-380.96	12.64	34.79	19.00	0.00	4.38
8	Retention-scaling	Output-aim-scaling	-324.86	12.48	-380.96	12.63	34.77	19.00	0.00	4.38
9	State-aim-scaling	Output-aim-scaling	-380.96	12.64	-380.96	12.63	-0.28	19.00	1.00	-0.00

S1 Table. Experiment 1 two-state model comparison statistics. Abbreviations are *std* is standard deviation, T is the t-statistic, *dof* is degrees of freedom, *p-corr* is the p-value corrected for multiple comparisons, and *hedges* is Hedges g.
