# Supplementary material for: Error-independent effect of sensory uncertainty on motor learning when both feedforward and feedback control processes are engaged
Source: PLoS Comput Biol. 2023 Sep 8;19(9):e1010526. doi: 10.1371/journal.pcbi.1010526 (PMC10522034; doi:10.1371/journal.pcbi.1010526)
Supplement: S2 Table — Abbreviations are std is standard deviation, T is the t-statistic, dof is degrees of freedom, p-corr is the p-value corrected for multiple comparisons, and hedges is Hedges g. (PDF) [file pcbi.1010526.s002.pdf]

row	A	B	mean(A)	std(A)	mean(B)	std(B)	T	dof	p-corr	hedges
0	Bias-scaling	Error-scaling	-371.98	13.38	-344.04	12.80	-9.05	19.00	1.00	-2.09
1	Bias-scaling	Retention-scaling	-371.98	13.38	-368.48	15.18	-0.92	19.00	1.00	-0.24
2	Bias-scaling	State-aim-scaling	-371.98	13.38	-389.01	12.81	11.43	19.00	0.00	1.27
3	Bias-scaling	Output-aim-scaling	-371.98	13.38	-388.88	12.64	12.76	19.00	0.00	1.27
4	Error-scaling	Retention-scaling	-344.04	12.80	-368.48	15.18	9.38	19.00	0.00	1.71
5	Error-scaling	State-aim-scaling	-344.04	12.80	-389.01	12.81	19.62	19.00	0.00	3.44
6	Error-scaling	Output-aim-scaling	-344.04	12.80	-388.88	12.64	16.66	19.00	0.00	3.46
7	Retention-scaling	State-aim-scaling	-368.48	15.18	-389.01	12.81	6.70	19.00	0.00	1.43
8	Retention-scaling	Output-aim-scaling	-368.48	15.18	-388.88	12.64	6.30	19.00	0.00	1.43
9	State-aim-scaling	Output-aim-scaling	-389.01	12.81	-388.88	12.64	-0.16	19.00	1.00	-0.01

S2 Table. Experiment 2 two-state model comparison statistics. Abbreviations are *std* is standard deviation, T is the t-statistic, *dof* is degrees of freedom, *p-corr* is the p-value corrected for multiple comparisons, and *hedges* is Hedges g.
