# Supplementary material for: Error-independent effect of sensory uncertainty on motor learning when both feedforward and feedback control processes are engaged
Source: PLoS Comput Biol. 2023 Sep 8;19(9):e1010526. doi: 10.1371/journal.pcbi.1010526 (PMC10522034; doi:10.1371/journal.pcbi.1010526)
Supplement: S3 Table — Abbreviations are std is standard deviation, T is the t-statistic, dof is degrees of freedom, p-corr is the p-value corrected for multiple comparisons, and hedges is Hedges g. (PDF) [file pcbi.1010526.s003.pdf]

row	A	B	mean(A)	std(A)	mean(B)	std(B)	T	dof	p-corr	hedges
0	Bias-scaling	Error-scaling	-451.14	14.42	-406.04	15.64	-14.96	19.00	1.00	-2.94
1	Bias-scaling	Retention-scaling	-451.14	14.42	-440.11	14.58	-4.07	19.00	1.00	-0.75
2	Bias-scaling	State-aim-scaling	-451.14	14.42	-444.09	14.49	-2.89	19.00	1.00	-0.48
3	Bias-scaling	Output-aim-scaling	-451.14	14.42	-444.46	13.95	-2.73	19.00	1.00	-0.46
4	Error-scaling	Retention-scaling	-406.04	15.64	-440.11	14.58	8.87	19.00	0.00	2.21
5	Error-scaling	State-aim-scaling	-406.04	15.64	-444.09	14.49	16.66	19.00	0.00	2.47
6	Error-scaling	Output-aim-scaling	-406.04	15.64	-444.46	13.95	17.44	19.00	0.00	2.54
7	Retention-scaling	State-aim-scaling	-440.11	14.58	-444.09	14.49	1.07	19.00	1.00	0.27
8	Retention-scaling	Output-aim-scaling	-440.11	14.58	-444.46	13.95	1.21	19.00	1.00	0.30
9	State-aim-scaling	Output-aim-scaling	-444.09	14.49	-444.46	13.95	0.85	19.00	1.00	0.03

S3 Table. Experiment 3 two-state model comparison statistics. Abbreviations are *std* is standard deviation, T is the t-statistic, *dof* is degrees of freedom, *p-corr* is the p-value corrected for multiple comparisons, and *hedges* is Hedges g.
