# Supplementary figures and images for: Error-independent effect of sensory uncertainty on motor learning when both feedforward and feedback control processes are engaged
Source: PLoS Comput Biol. 2023 Sep 8;19(9):e1010526. doi: 10.1371/journal.pcbi.1010526 (PMC10522034; doi:10.1371/journal.pcbi.1010526)

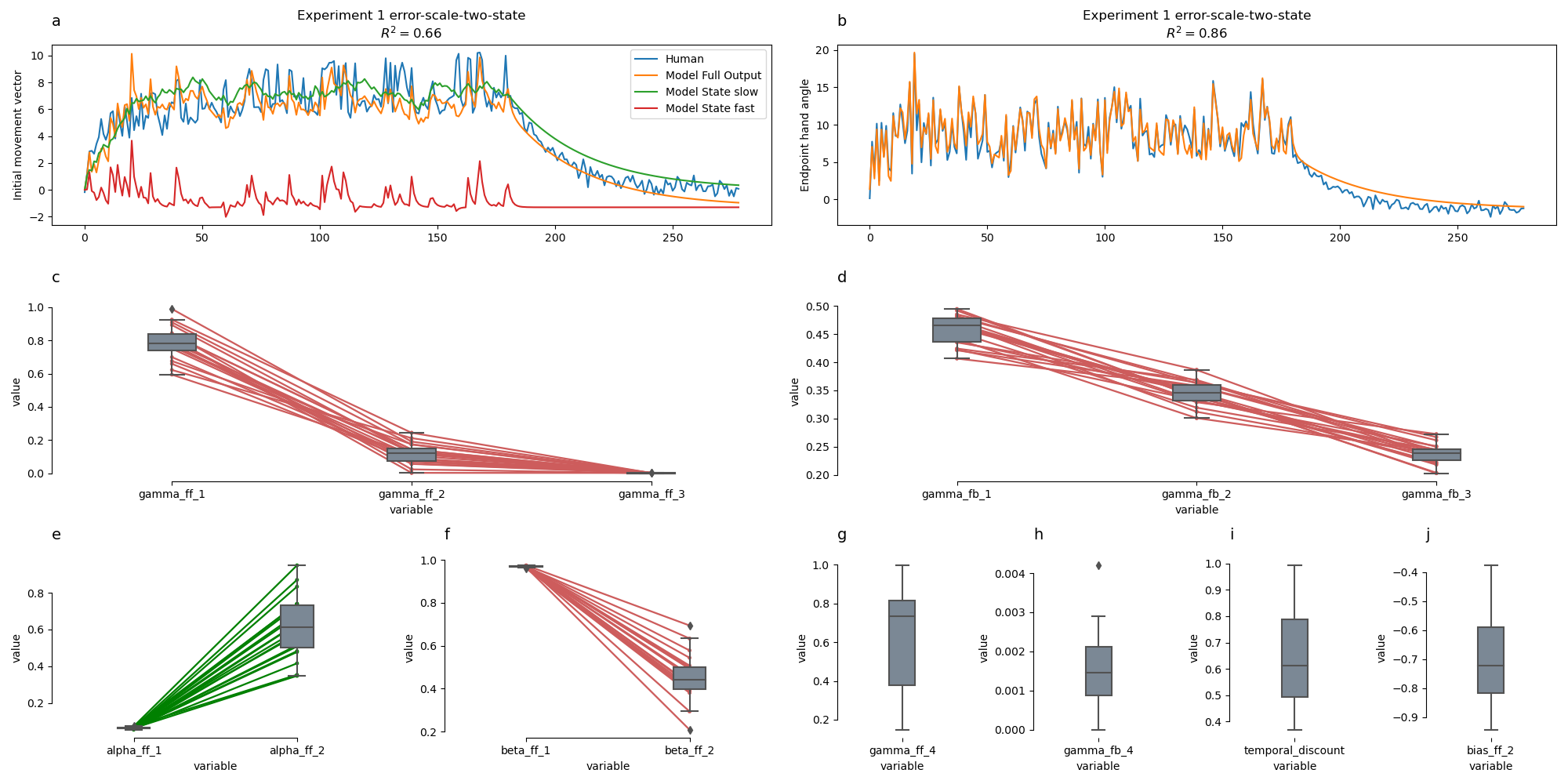

Supplement: S1 Fig — (TIF) [file pcbi.1010526.s004.tif]

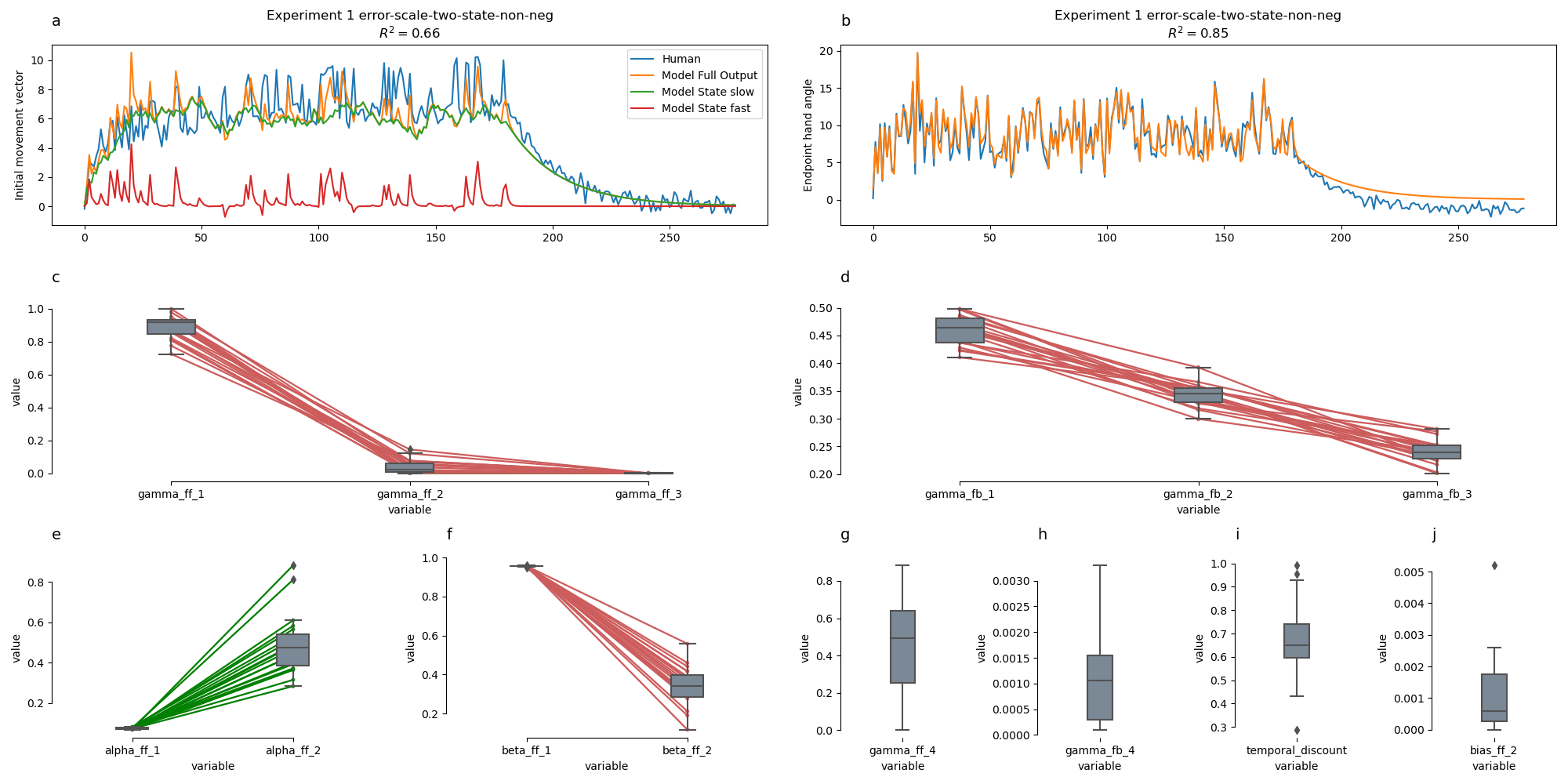

Supplement: S2 Fig — (TIF) [file pcbi.1010526.s005.tif]

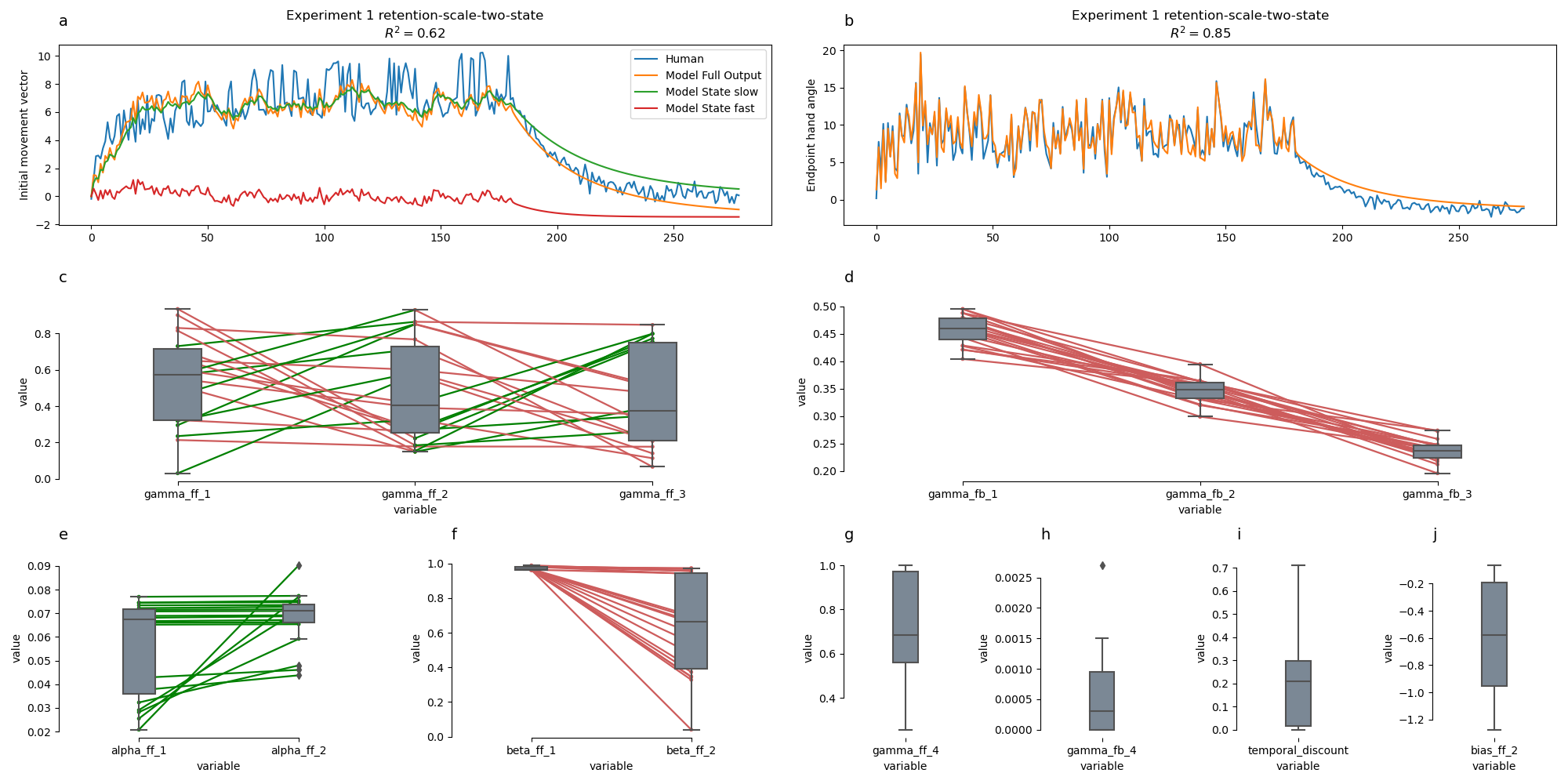

Supplement: S3 Fig — (TIF) [file pcbi.1010526.s006.tif]

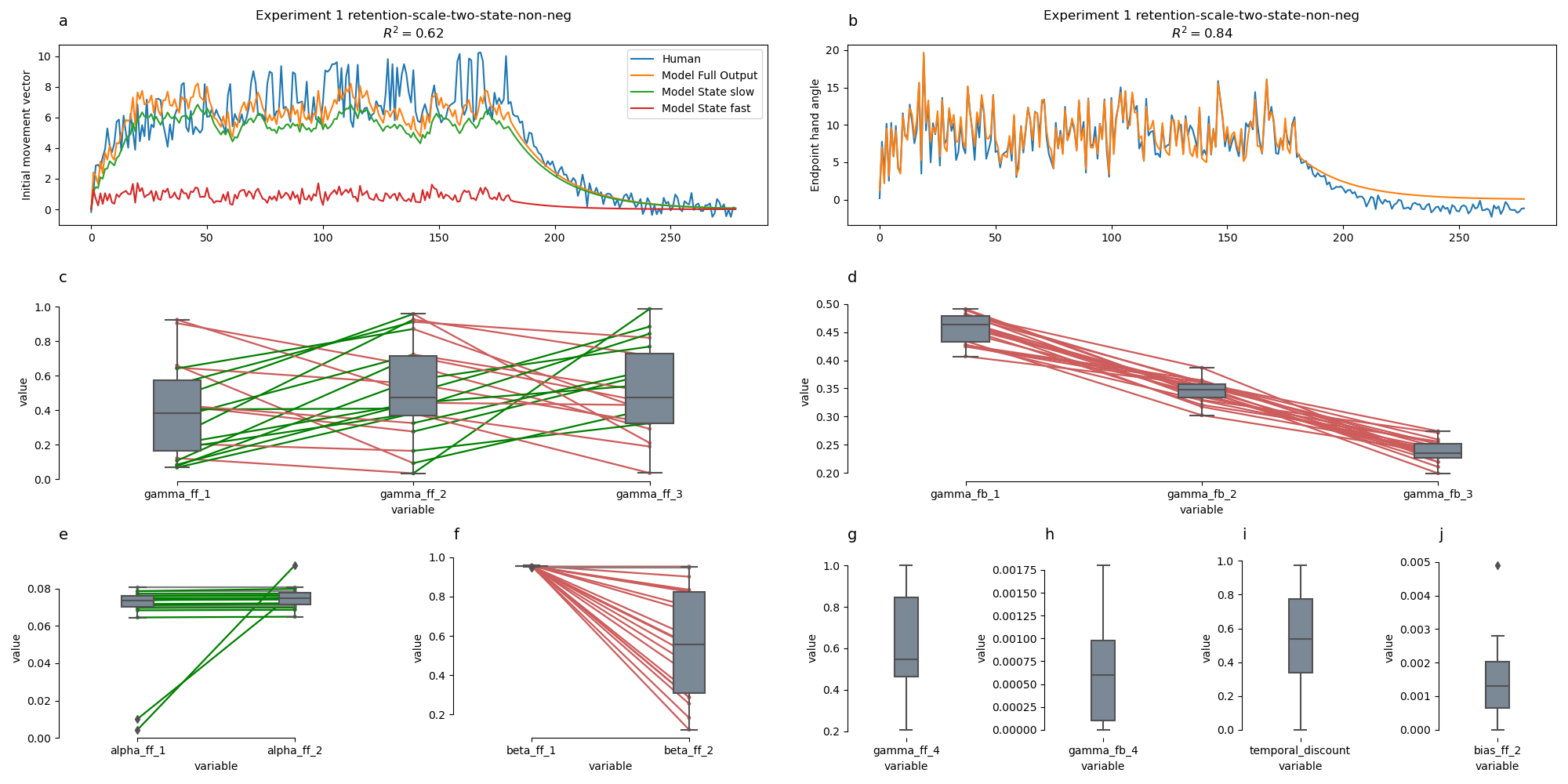

Supplement: S4 Fig — (TIF) [file pcbi.1010526.s007.tif]

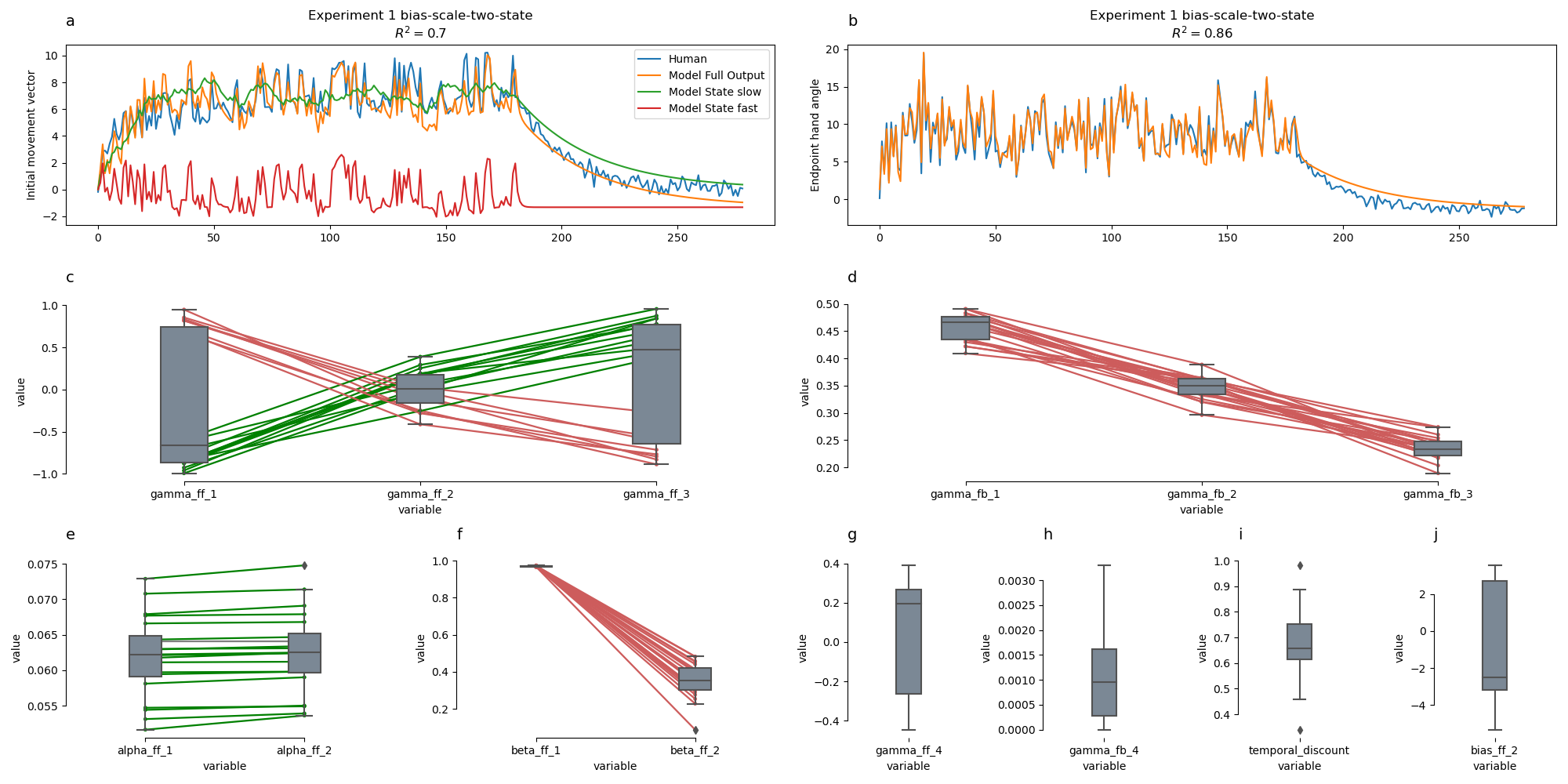

Supplement: S5 Fig — (TIF) [file pcbi.1010526.s008.tif]

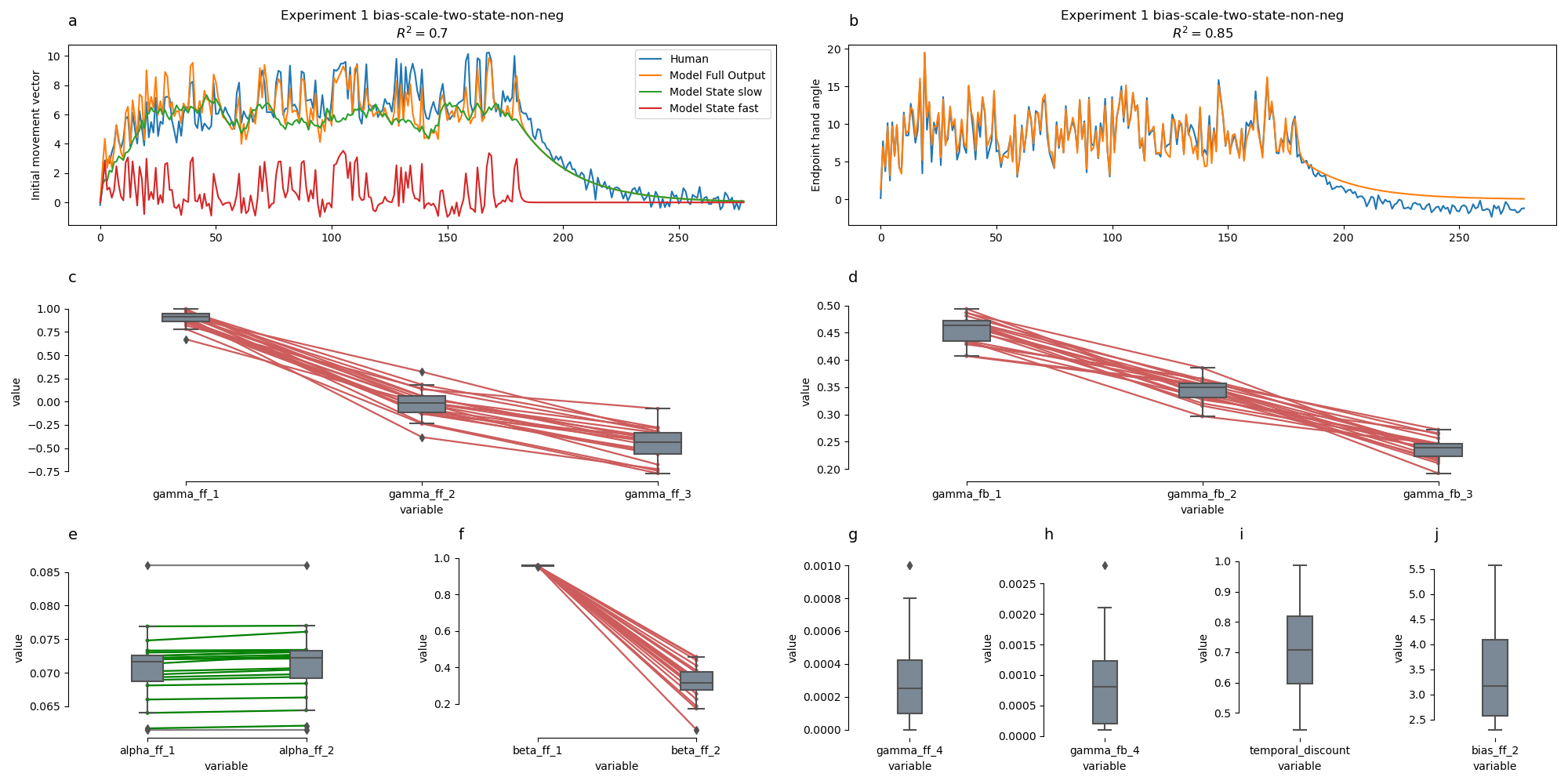

Supplement: S6 Fig — (TIF) [file pcbi.1010526.s009.tif]

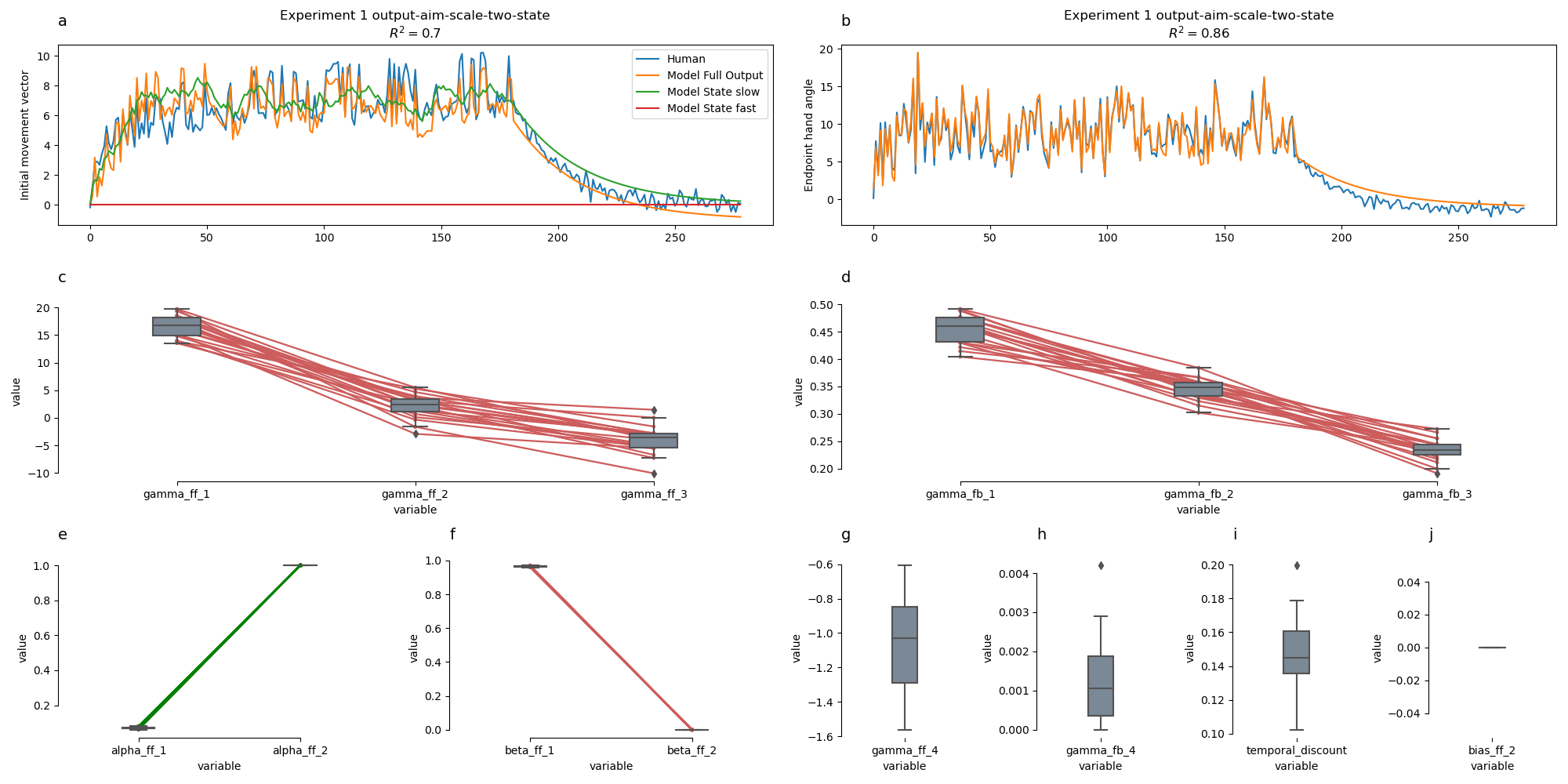

Supplement: S7 Fig — (TIF) [file pcbi.1010526.s010.tif]

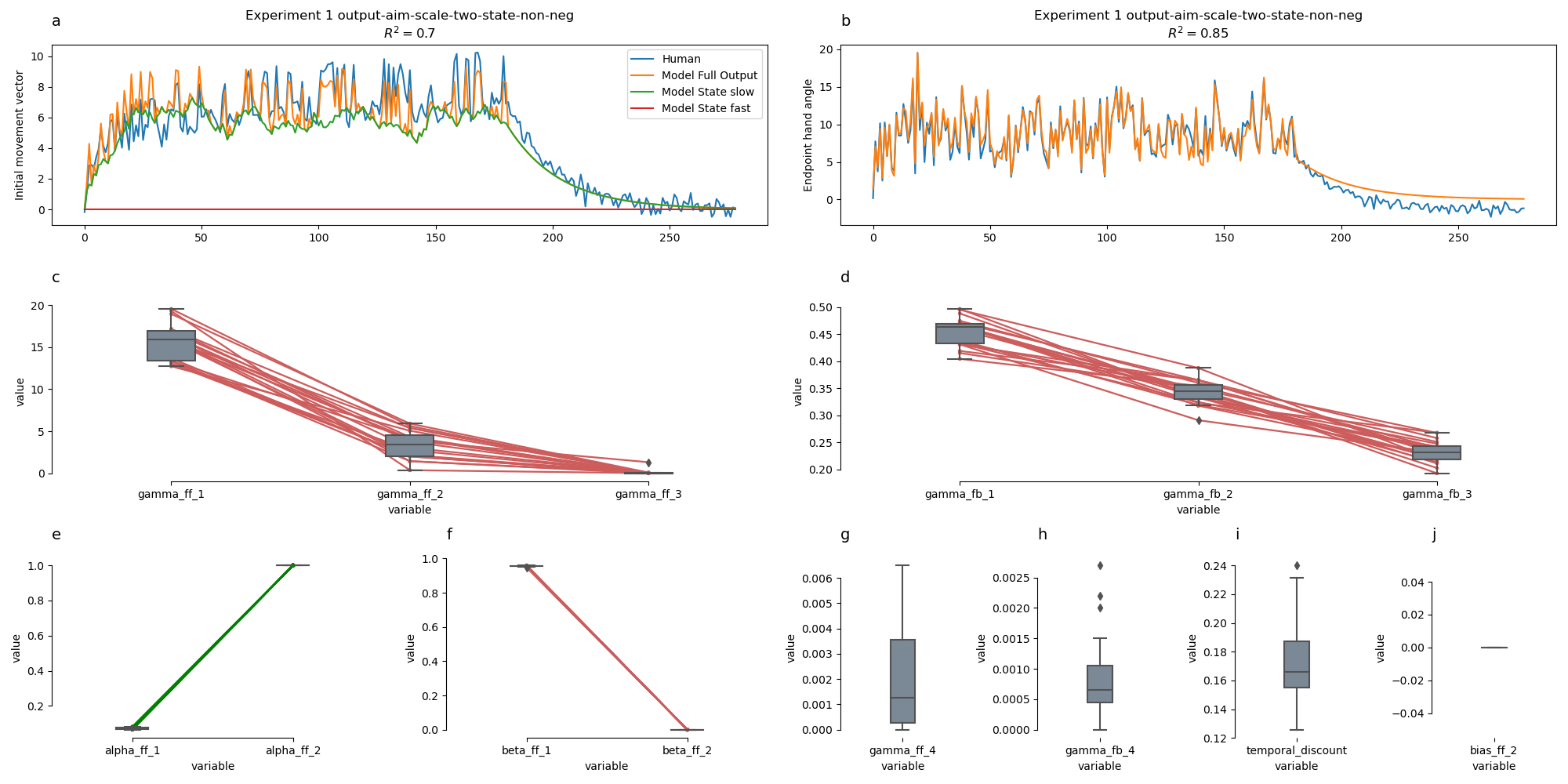

Supplement: S8 Fig — (TIF) [file pcbi.1010526.s011.tif]

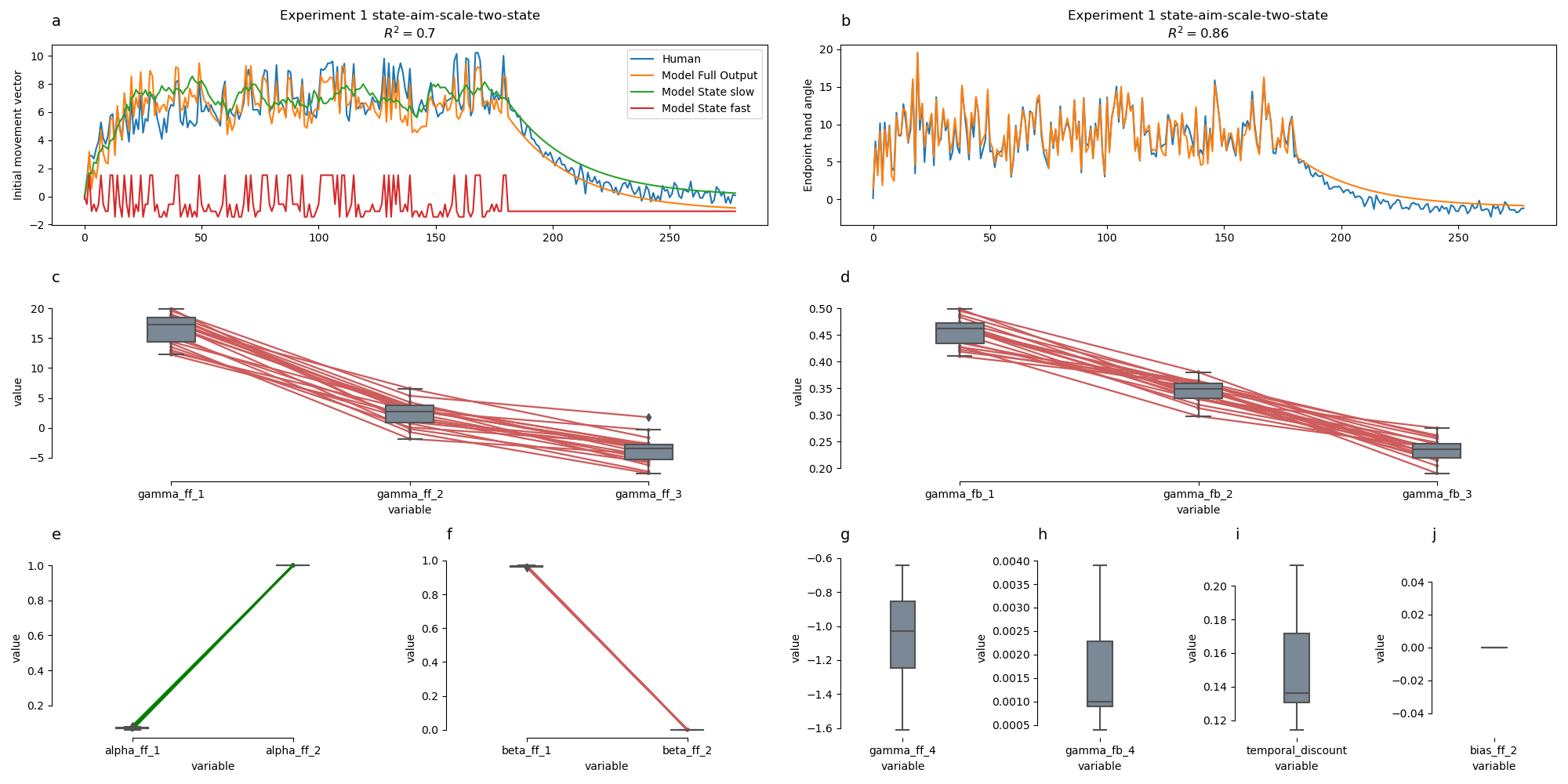

Supplement: S9 Fig — (TIF) [file pcbi.1010526.s012.tif]

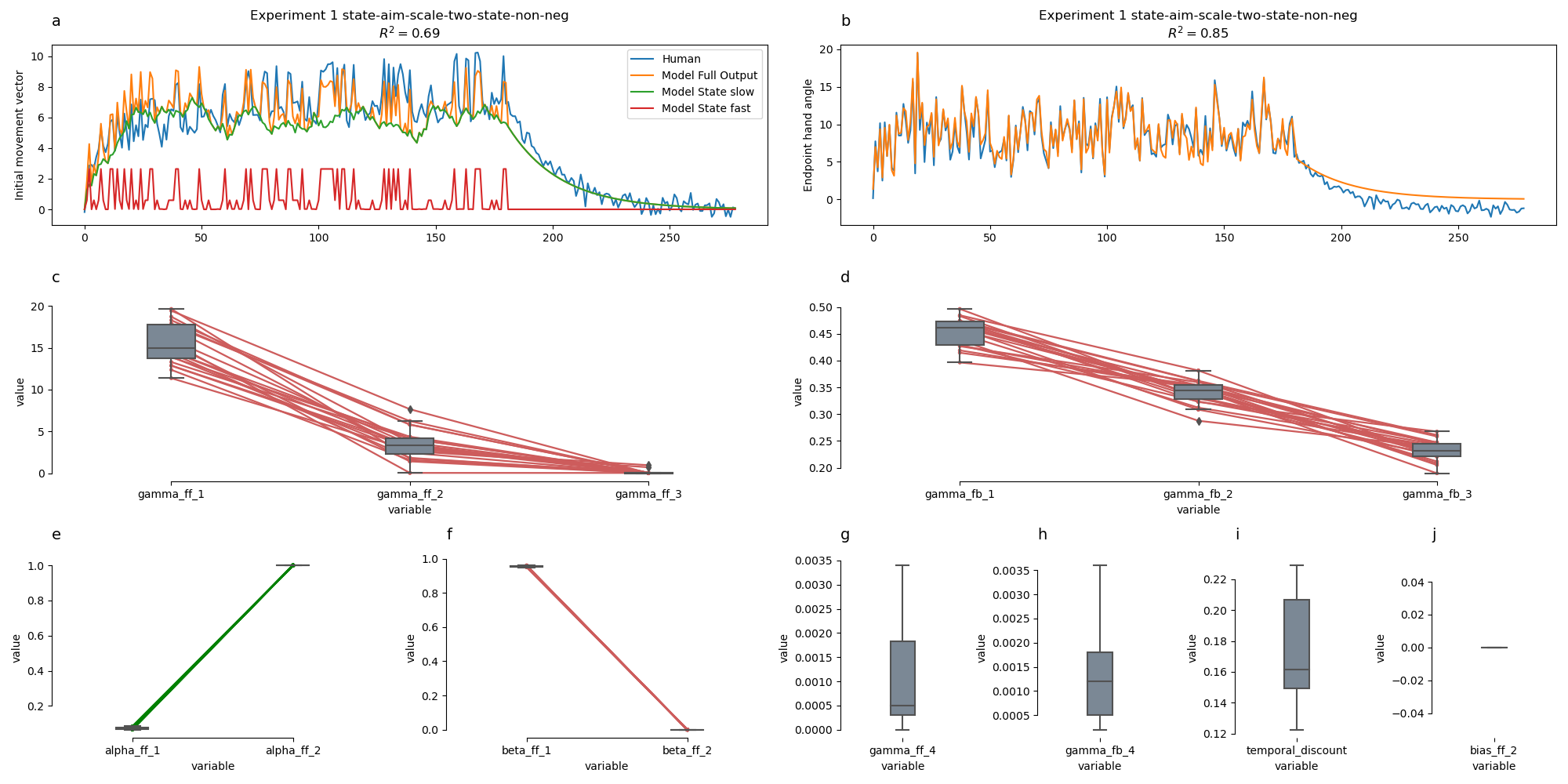

Supplement: S10 Fig — (TIF) [file pcbi.1010526.s013.tif]

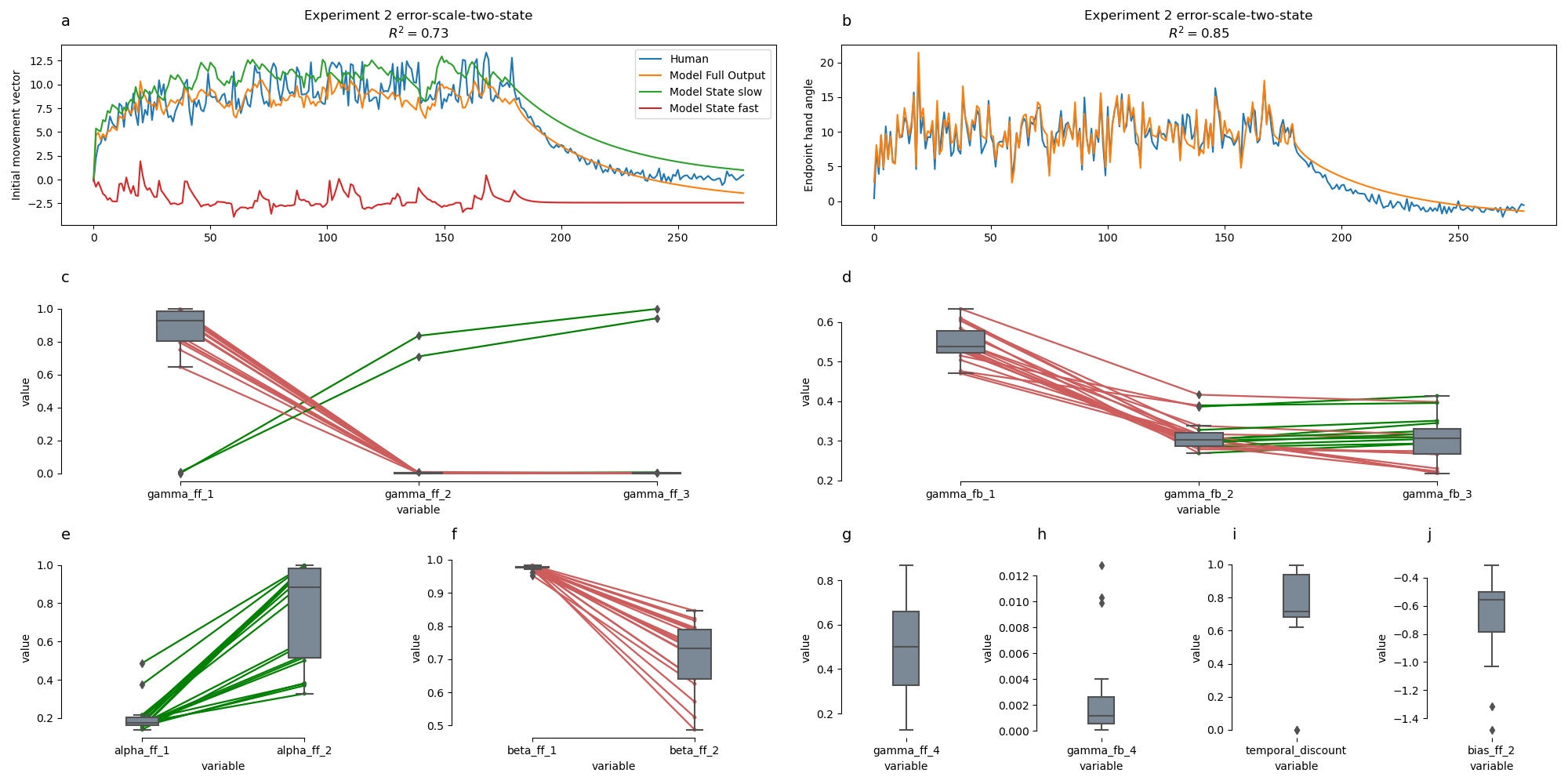

Supplement: S11 Fig — (TIF) [file pcbi.1010526.s014.tif]

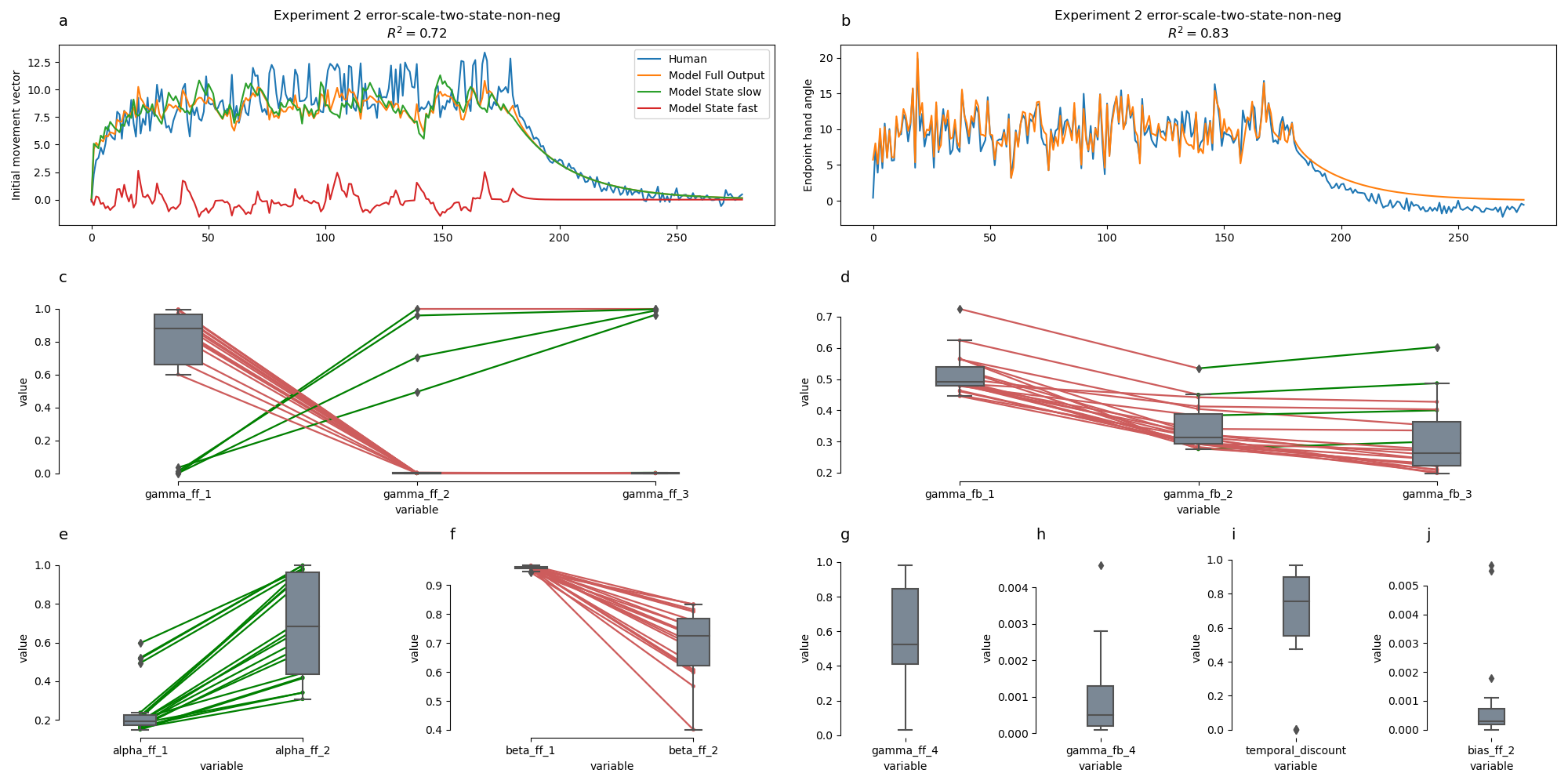

Supplement: S12 Fig — (TIF) [file pcbi.1010526.s015.tif]

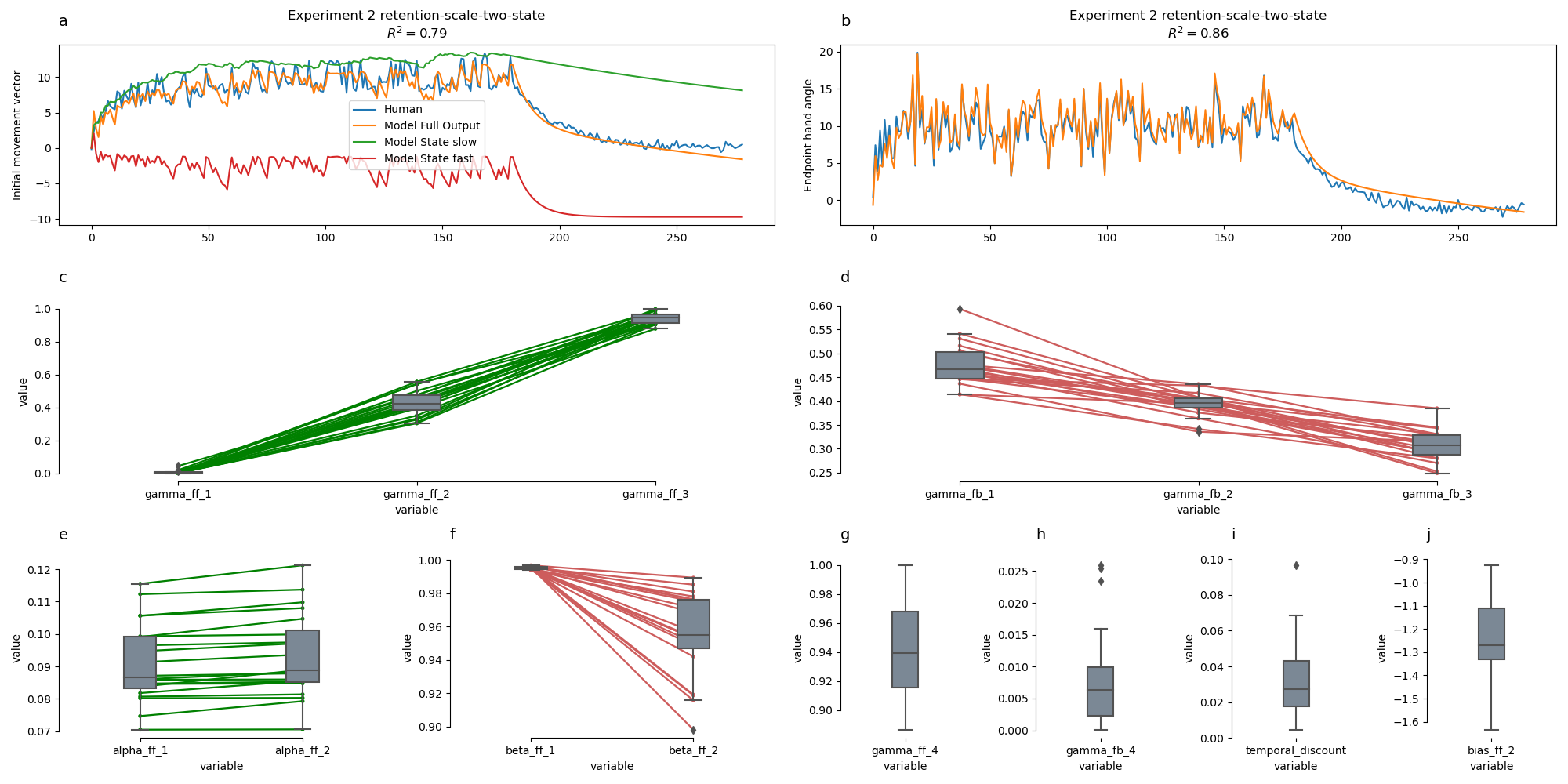

Supplement: S13 Fig — (TIF) [file pcbi.1010526.s016.tif]

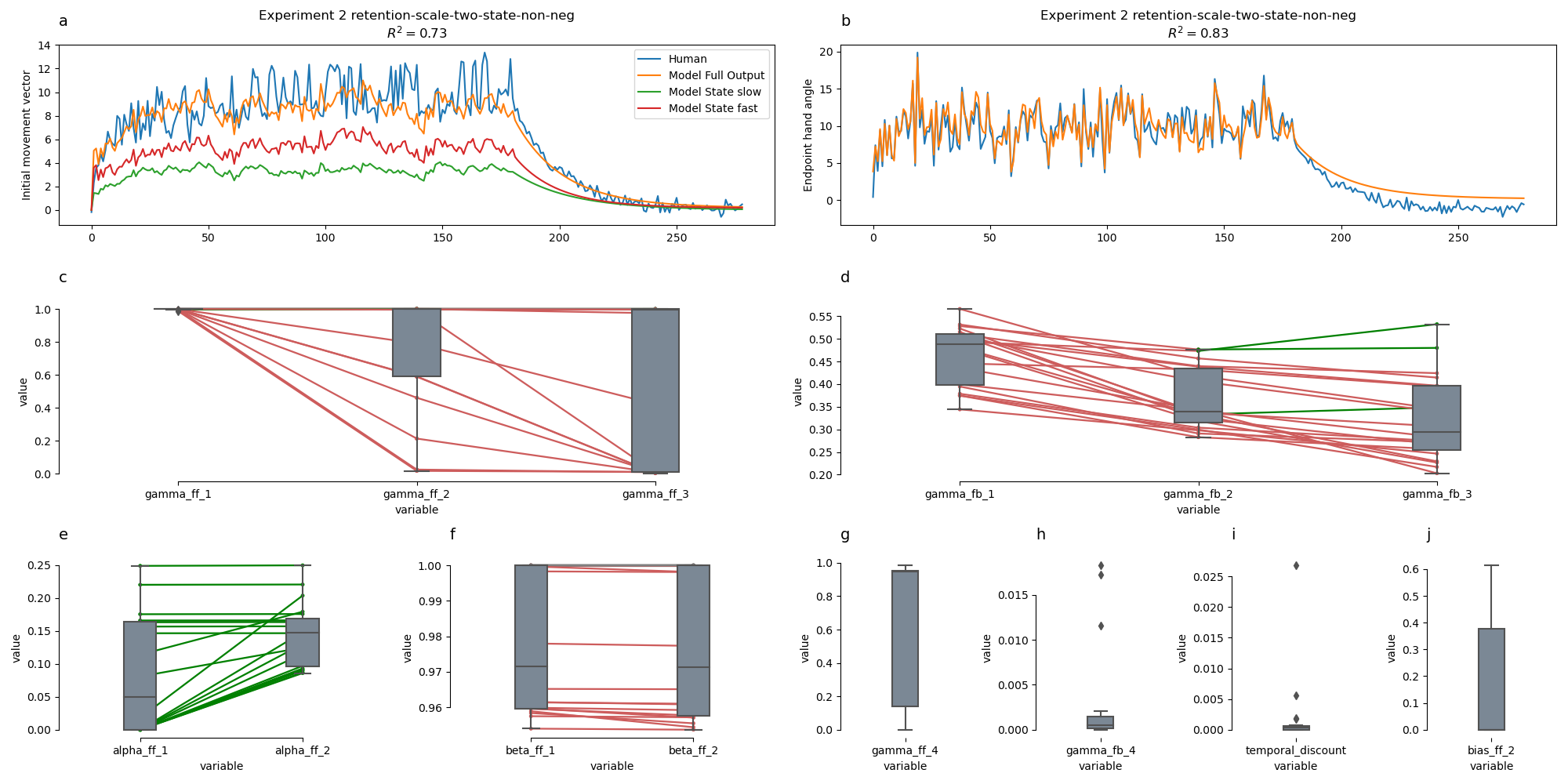

Supplement: S14 Fig — (TIF) [file pcbi.1010526.s017.tif]

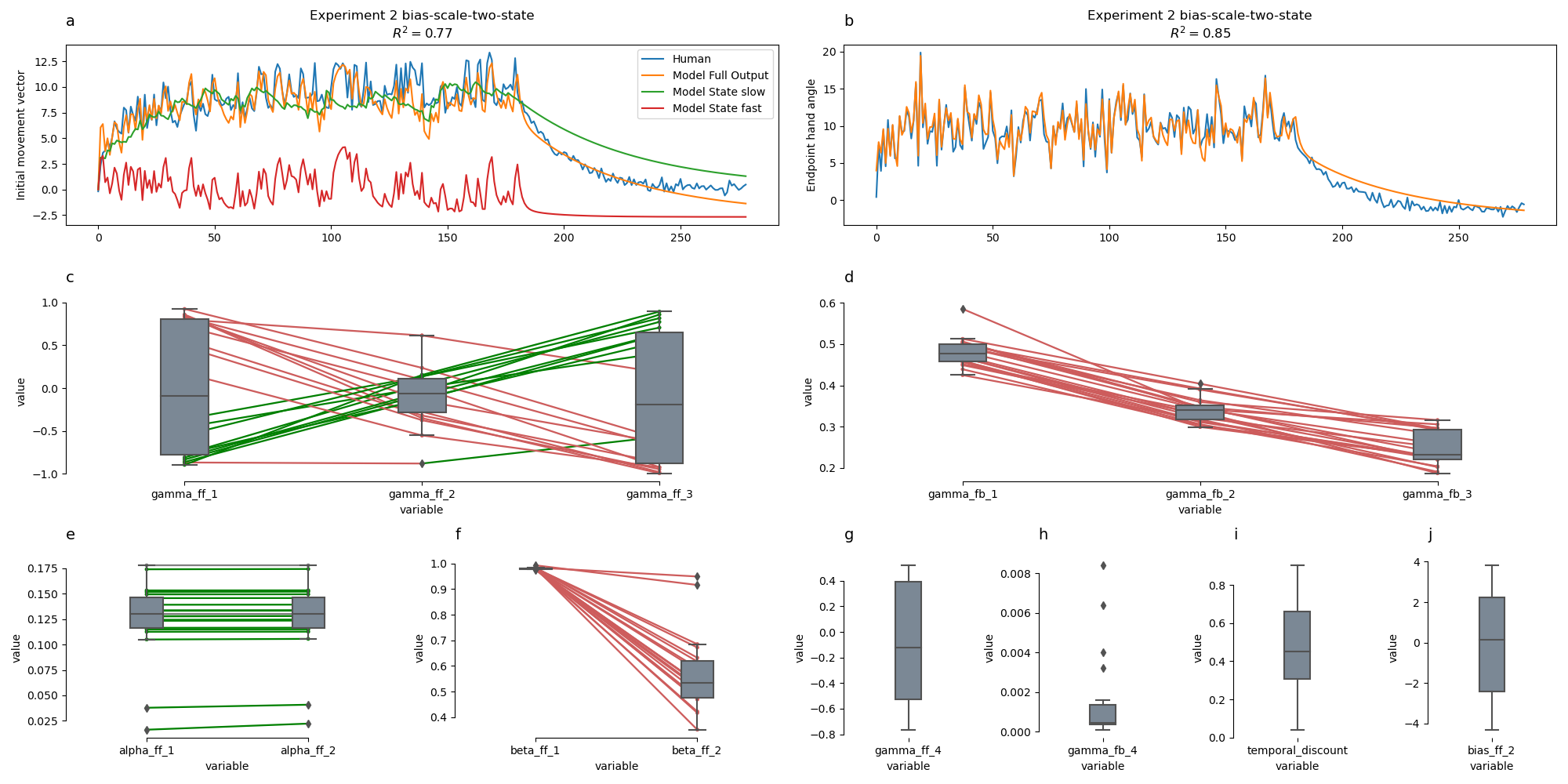

Supplement: S15 Fig — (TIF) [file pcbi.1010526.s018.tif]

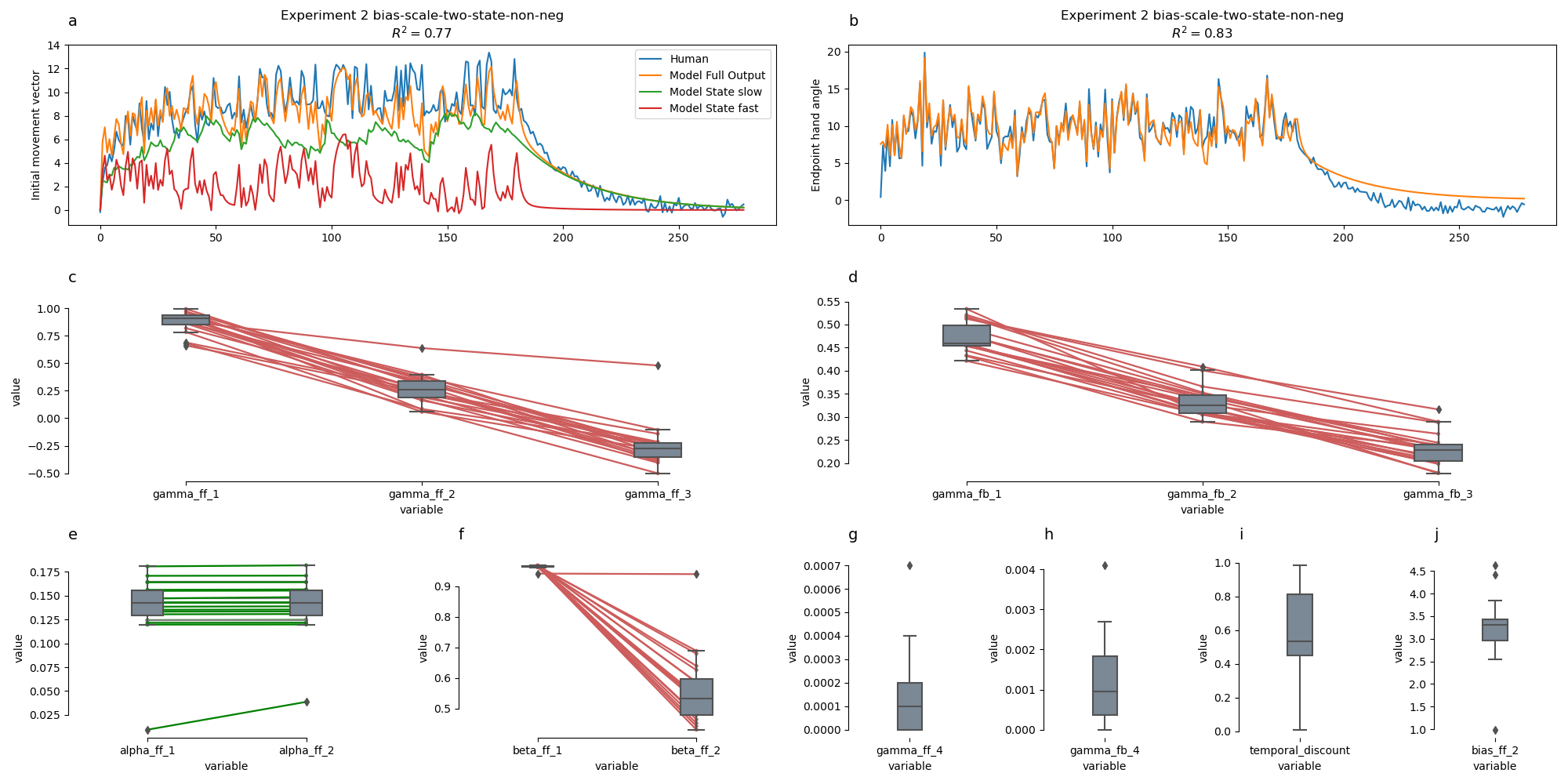

Supplement: S16 Fig — (TIF) [file pcbi.1010526.s019.tif]

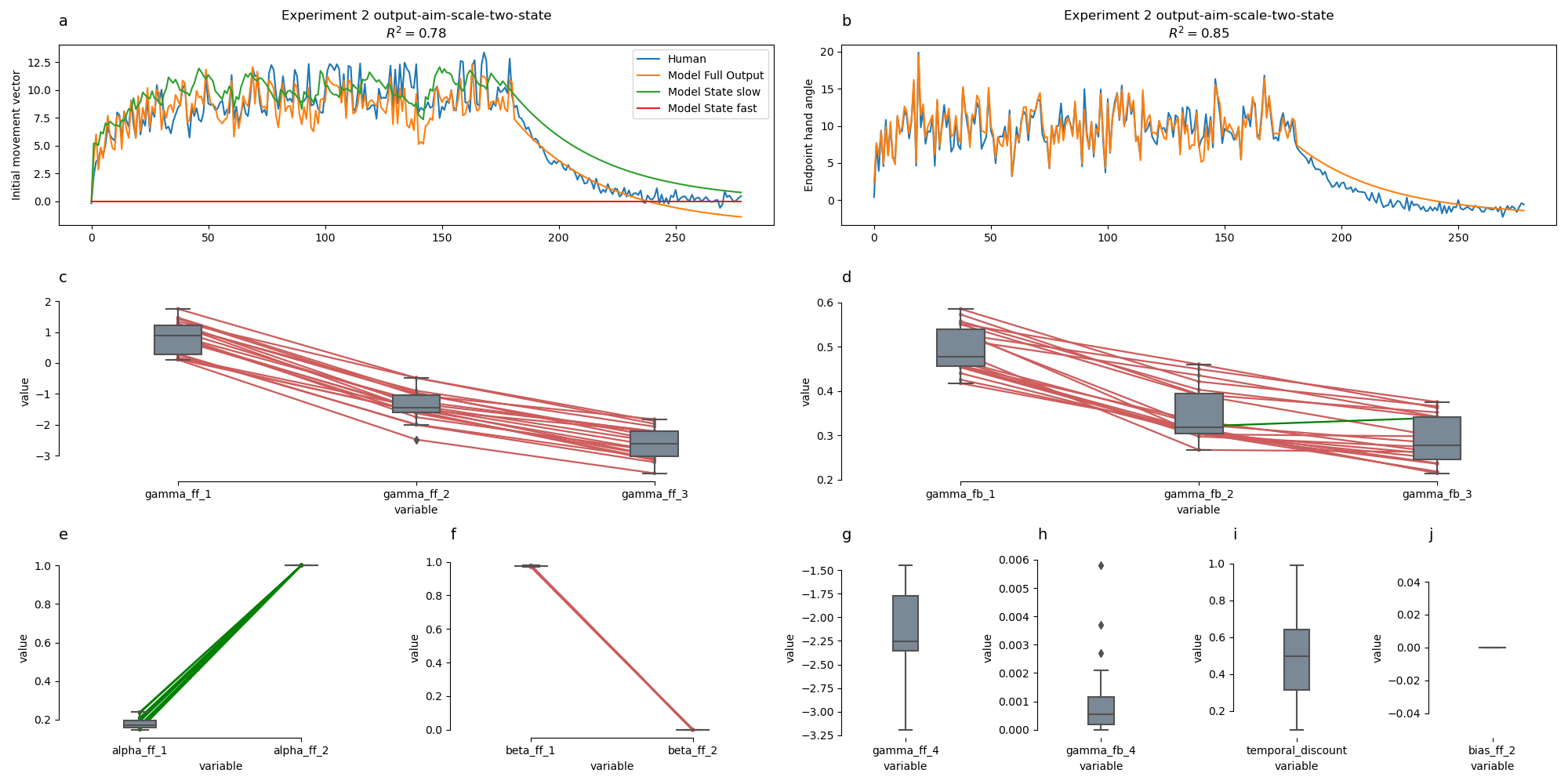

Supplement: S17 Fig — (TIF) [file pcbi.1010526.s020.tif]

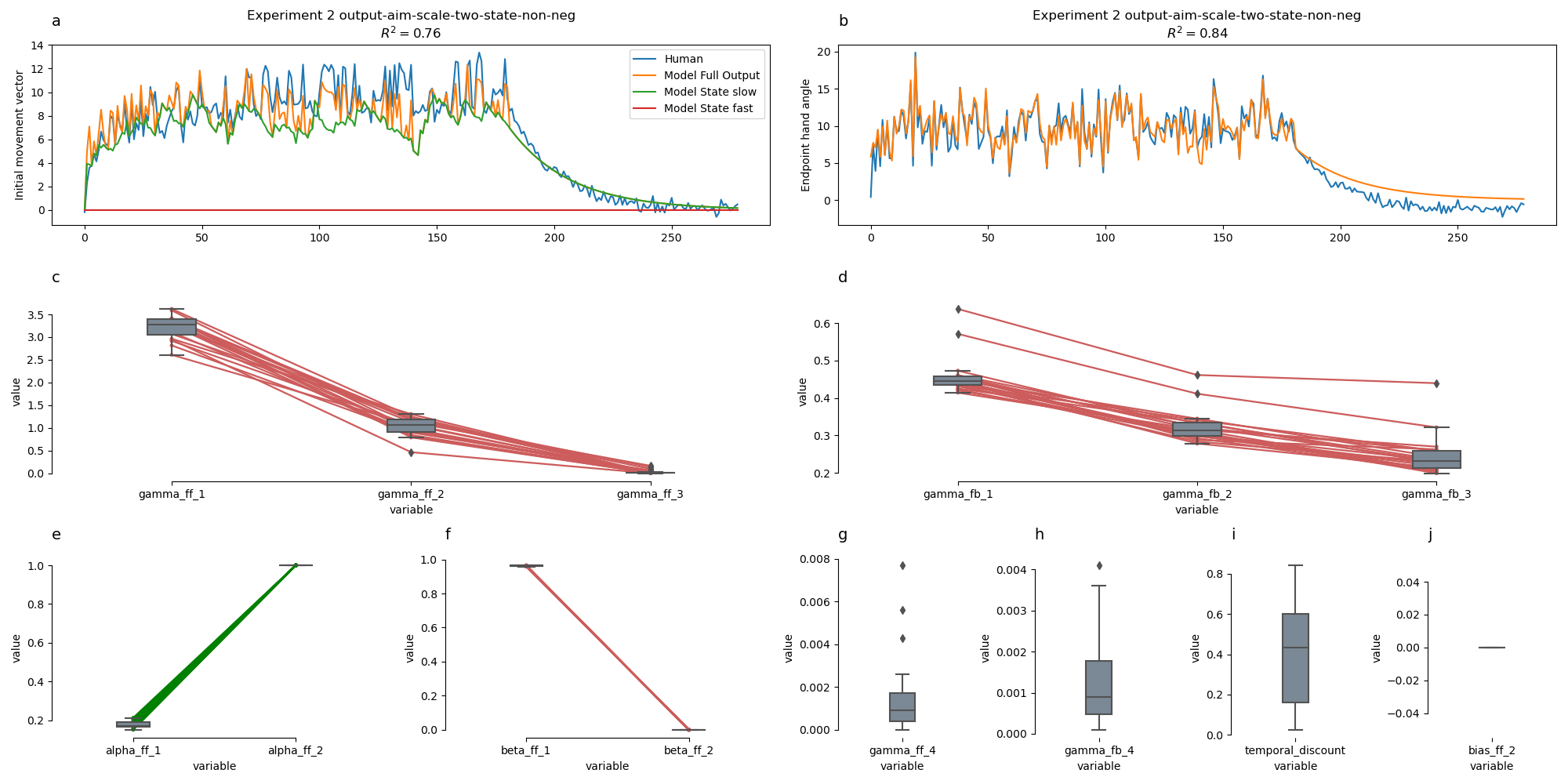

Supplement: S18 Fig — (TIF) [file pcbi.1010526.s021.tif]

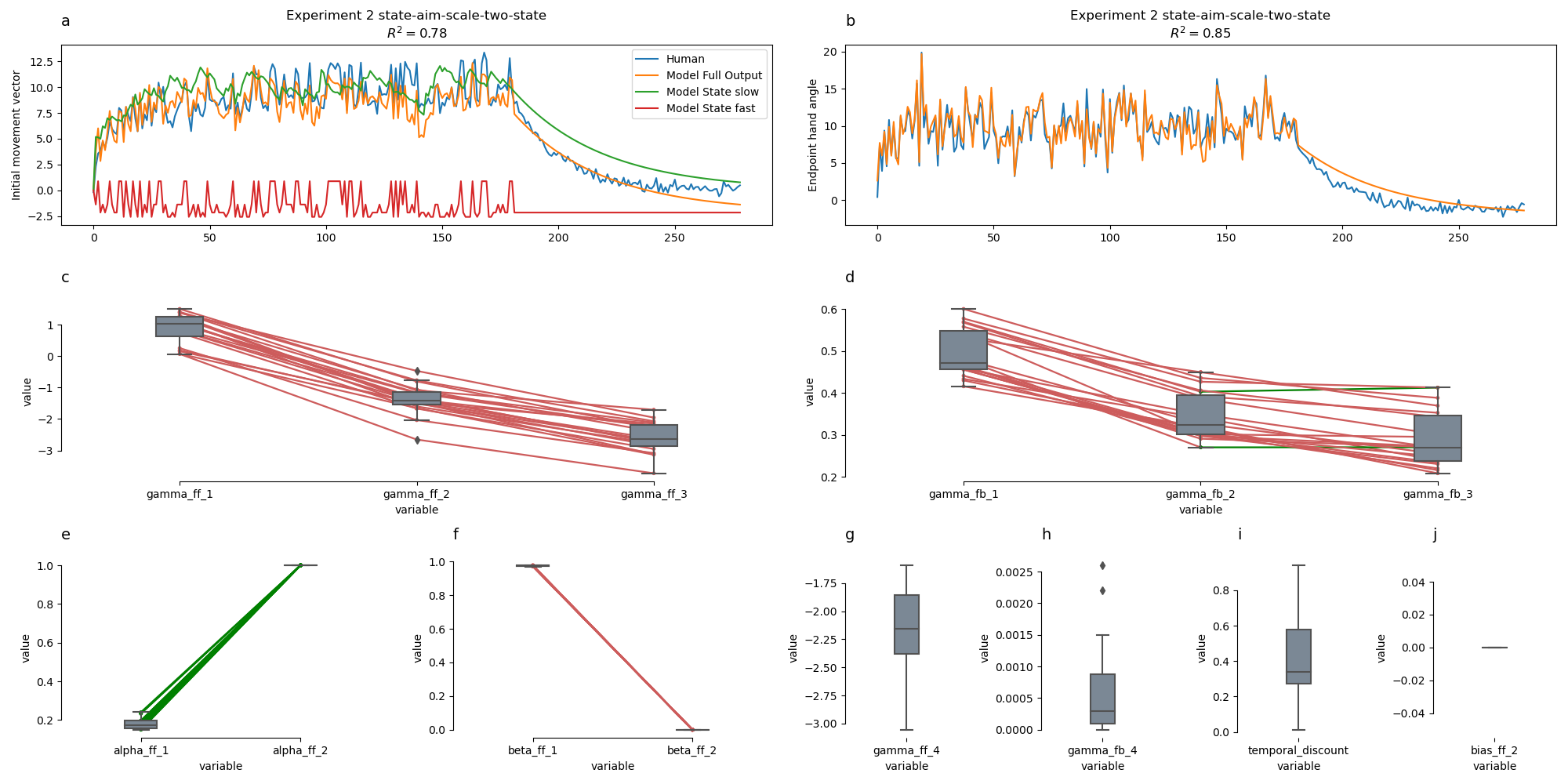

Supplement: S19 Fig — (TIF) [file pcbi.1010526.s022.tif]

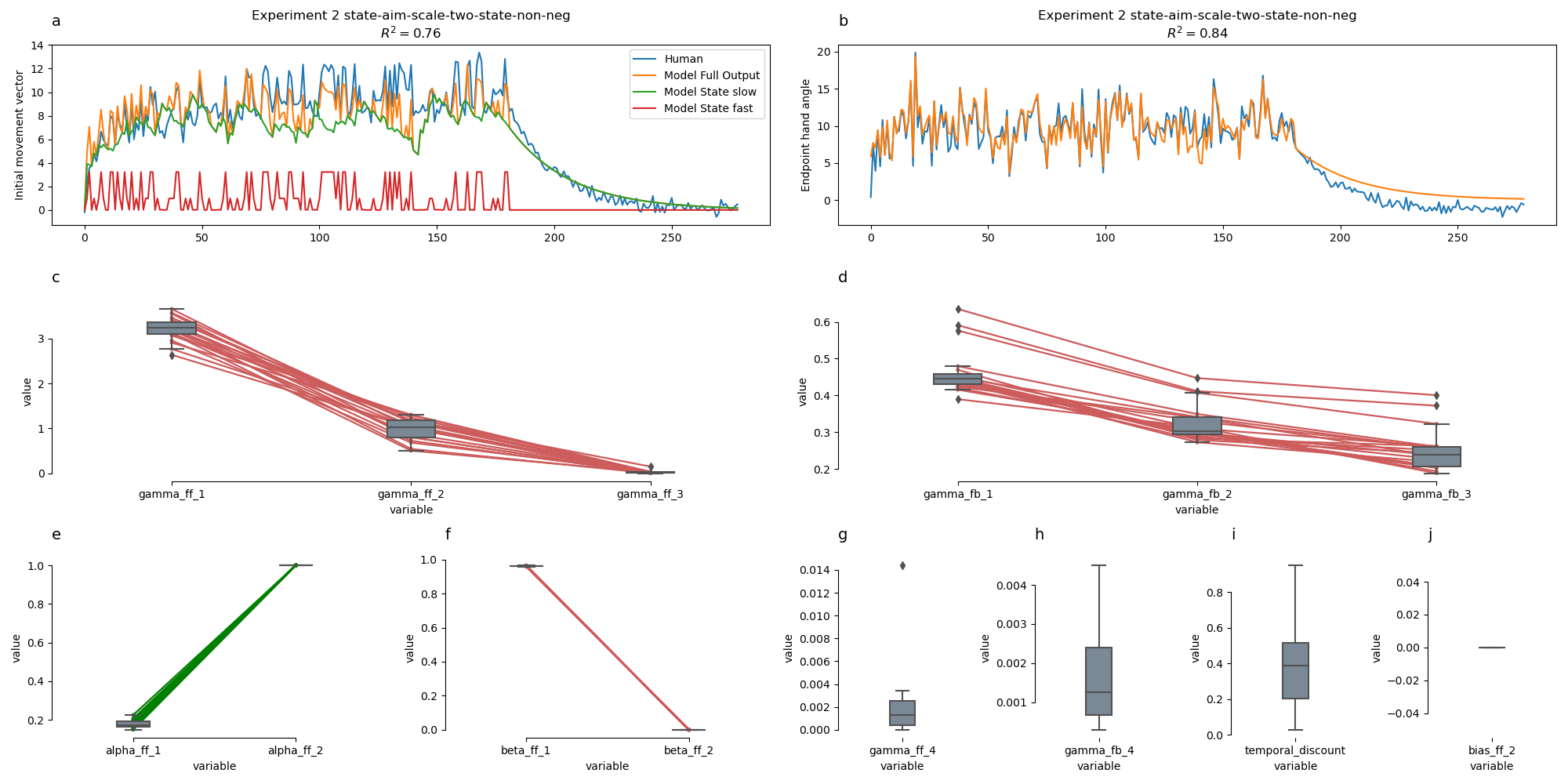

Supplement: S20 Fig — (TIF) [file pcbi.1010526.s023.tif]

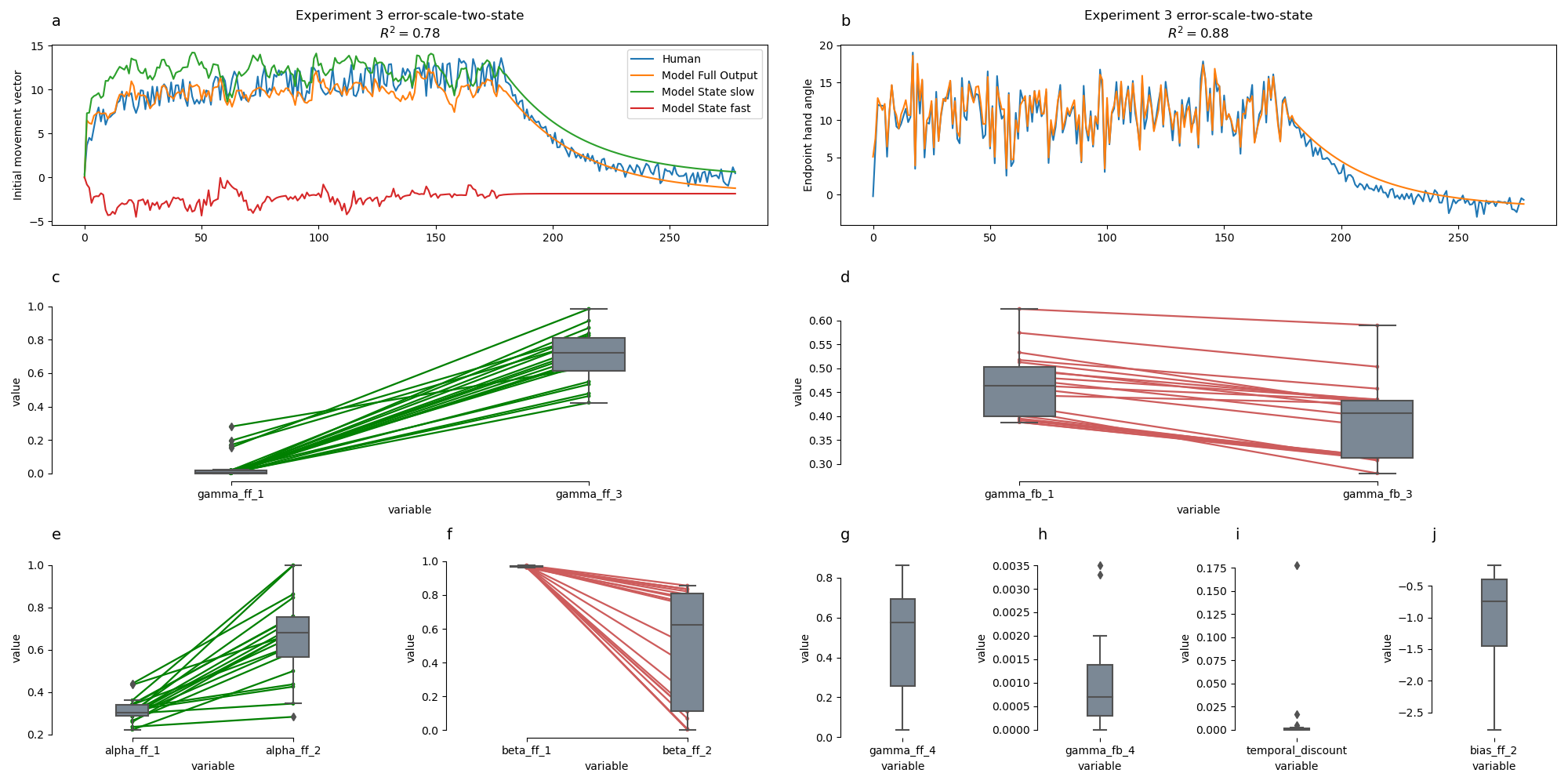

Supplement: S21 Fig — (TIF) [file pcbi.1010526.s024.tif]

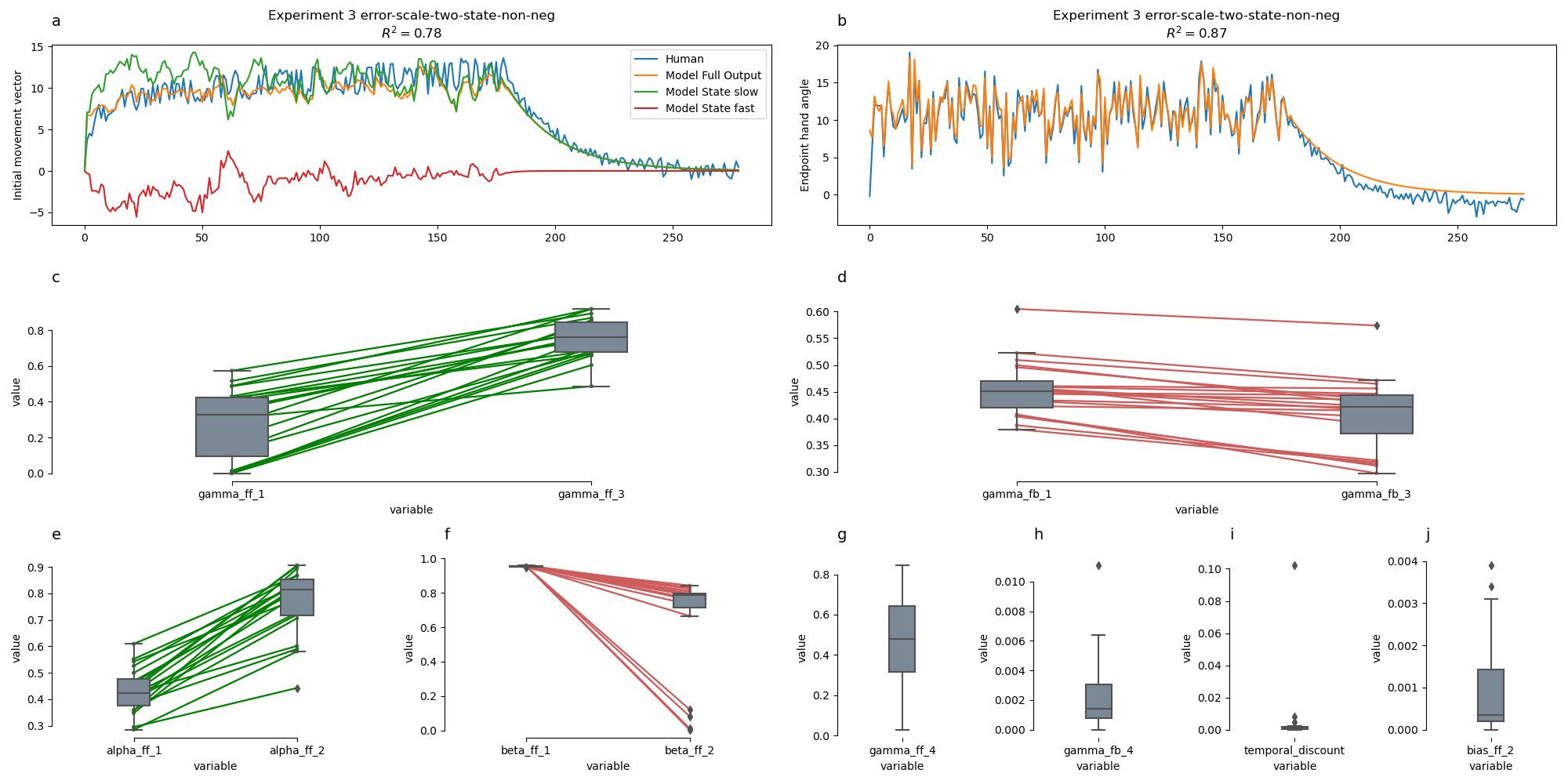

Supplement: S22 Fig — (TIF) [file pcbi.1010526.s025.tif]

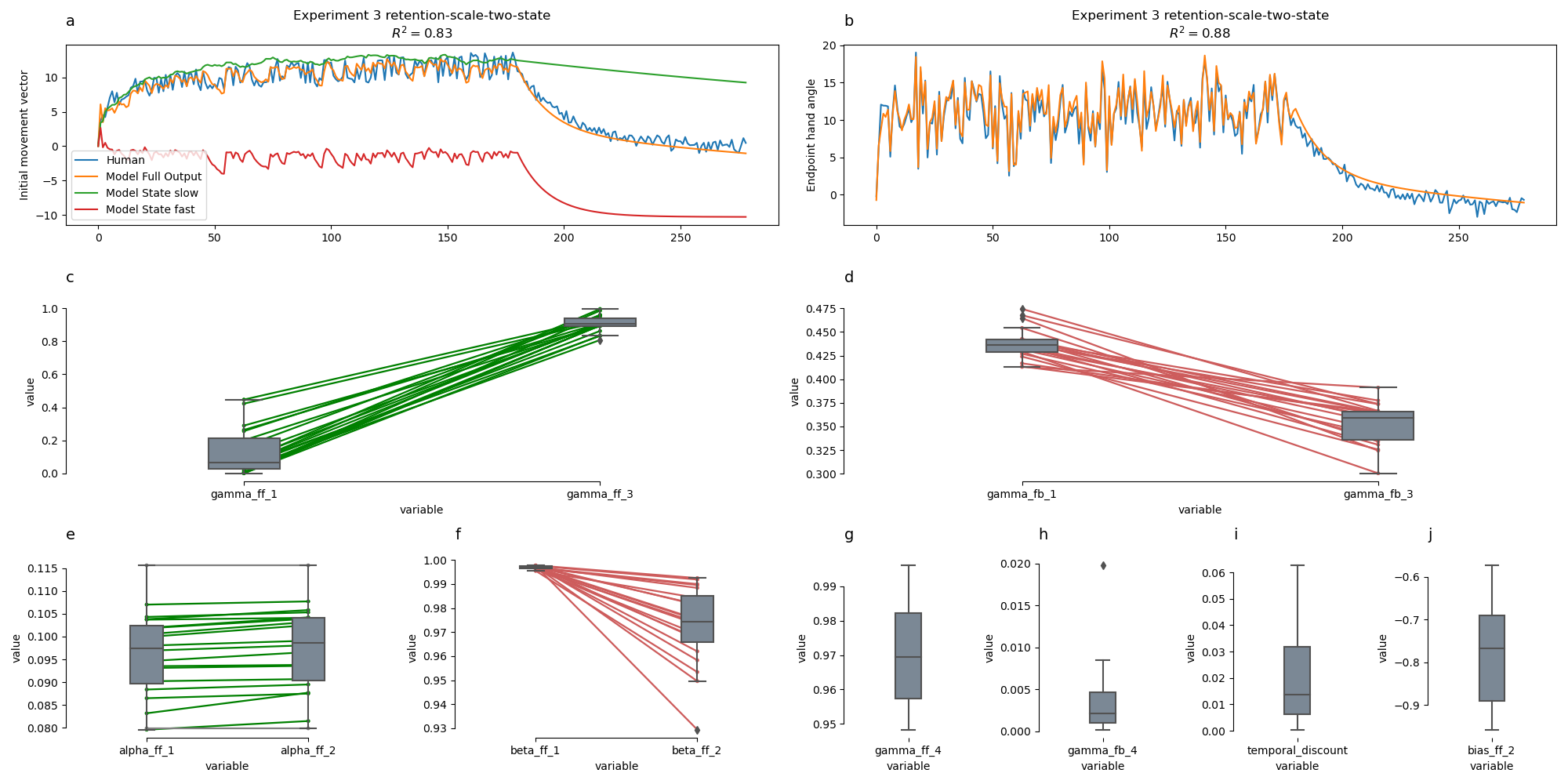

Supplement: S23 Fig — (TIF) [file pcbi.1010526.s026.tif]

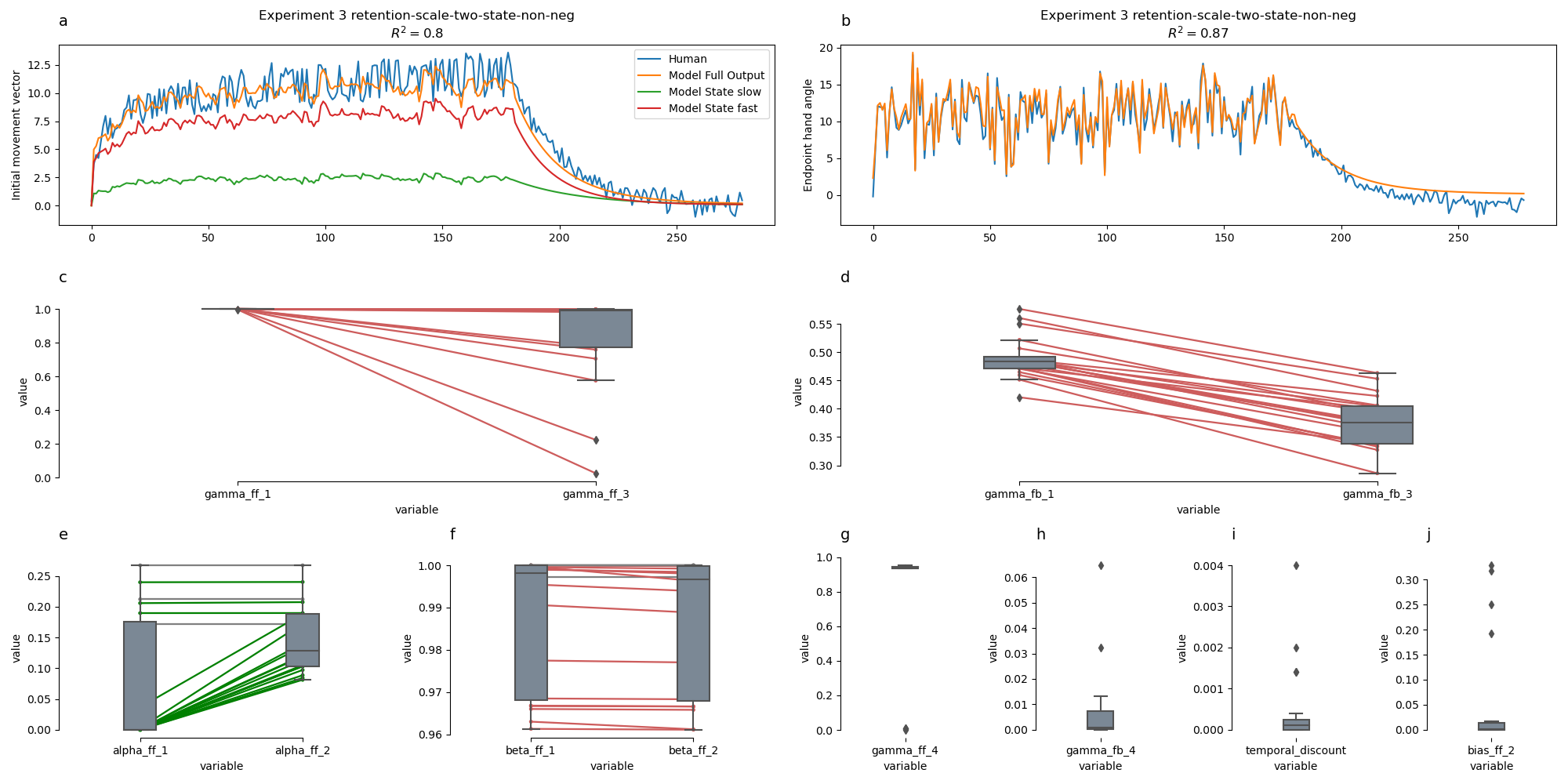

Supplement: S24 Fig — (TIF) [file pcbi.1010526.s027.tif]

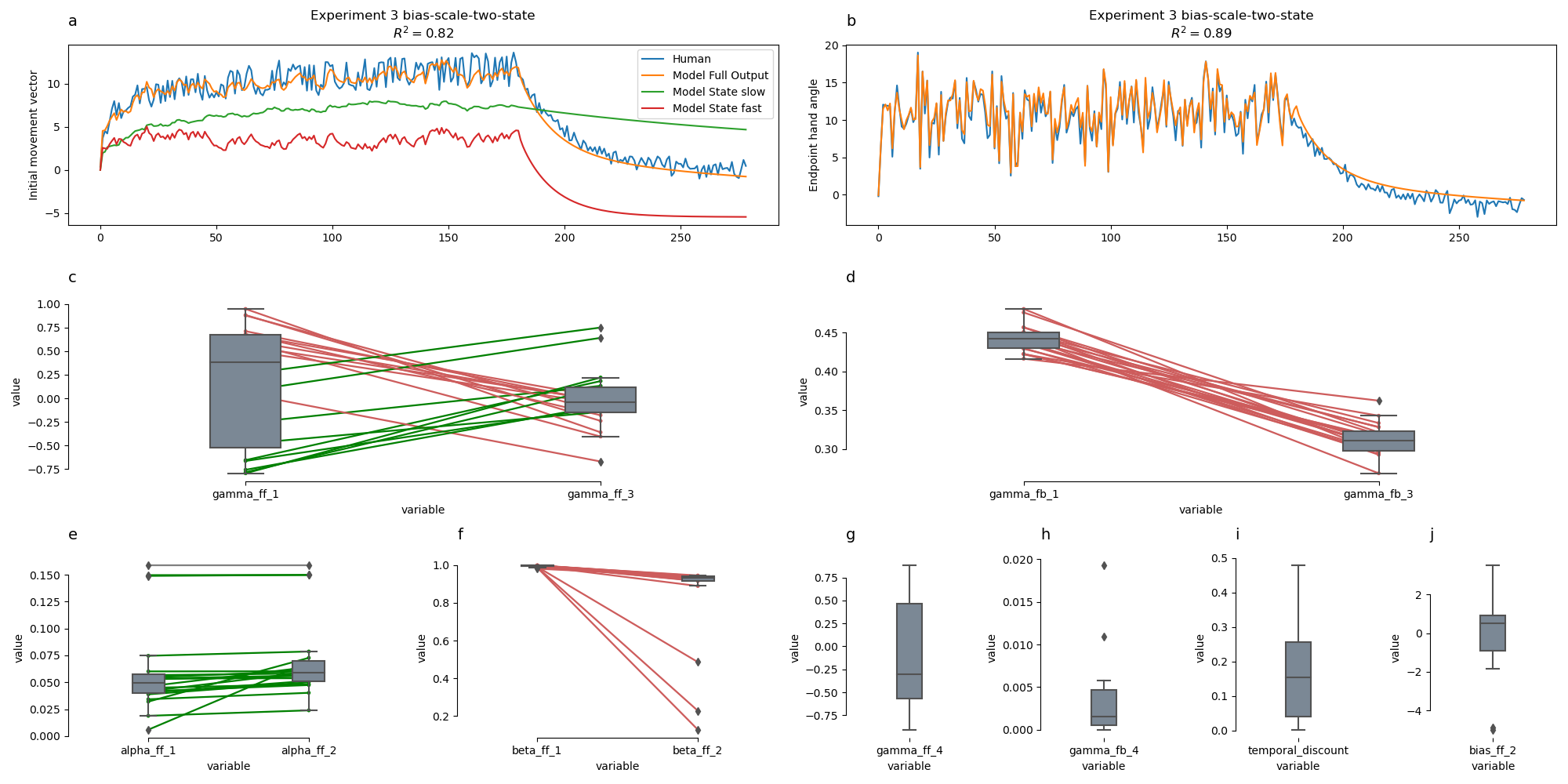

Supplement: S25 Fig — (TIF) [file pcbi.1010526.s028.tif]

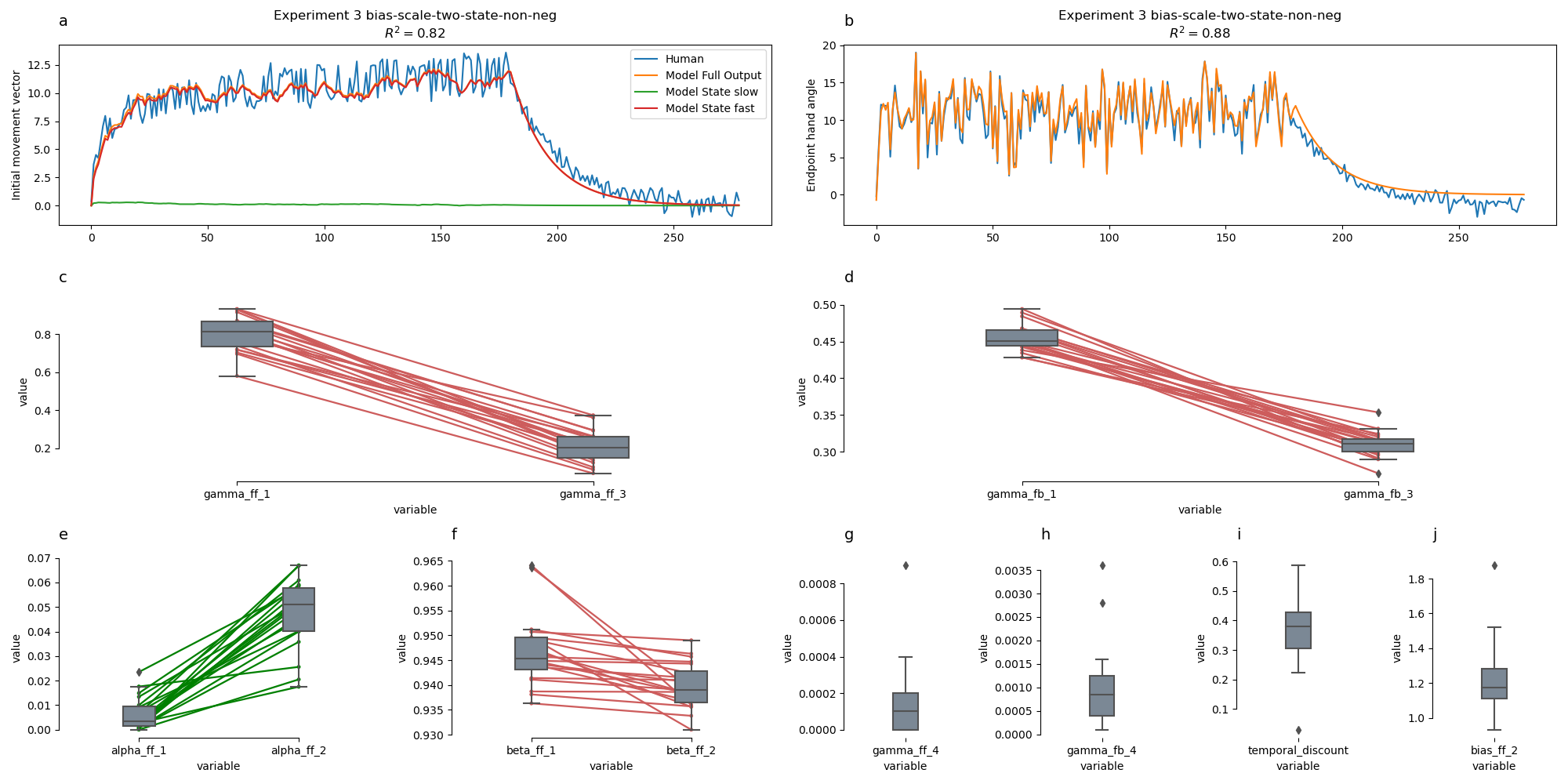

Supplement: S26 Fig — (TIF) [file pcbi.1010526.s029.tif]

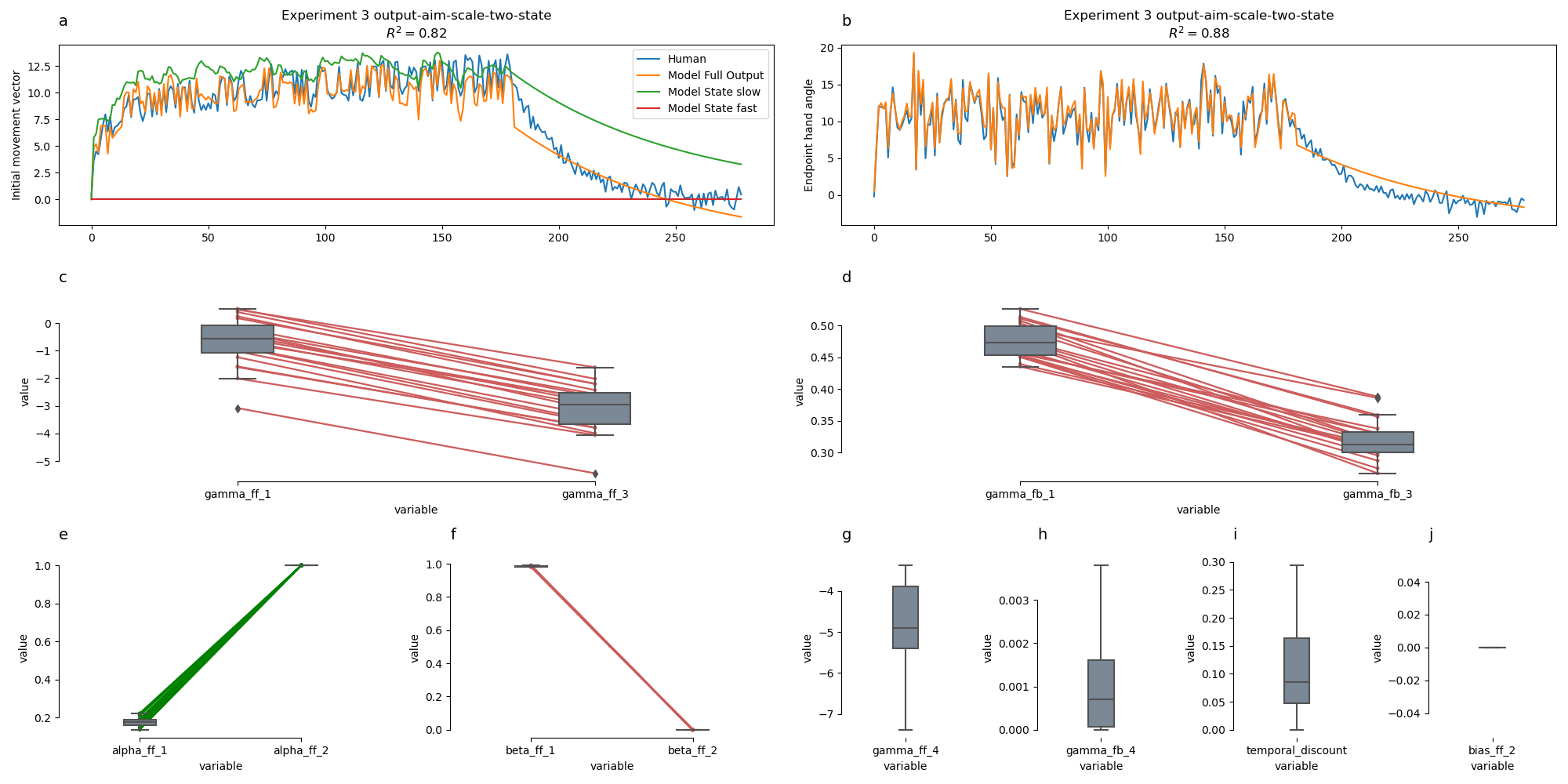

Supplement: S27 Fig — (TIF) [file pcbi.1010526.s030.tif]

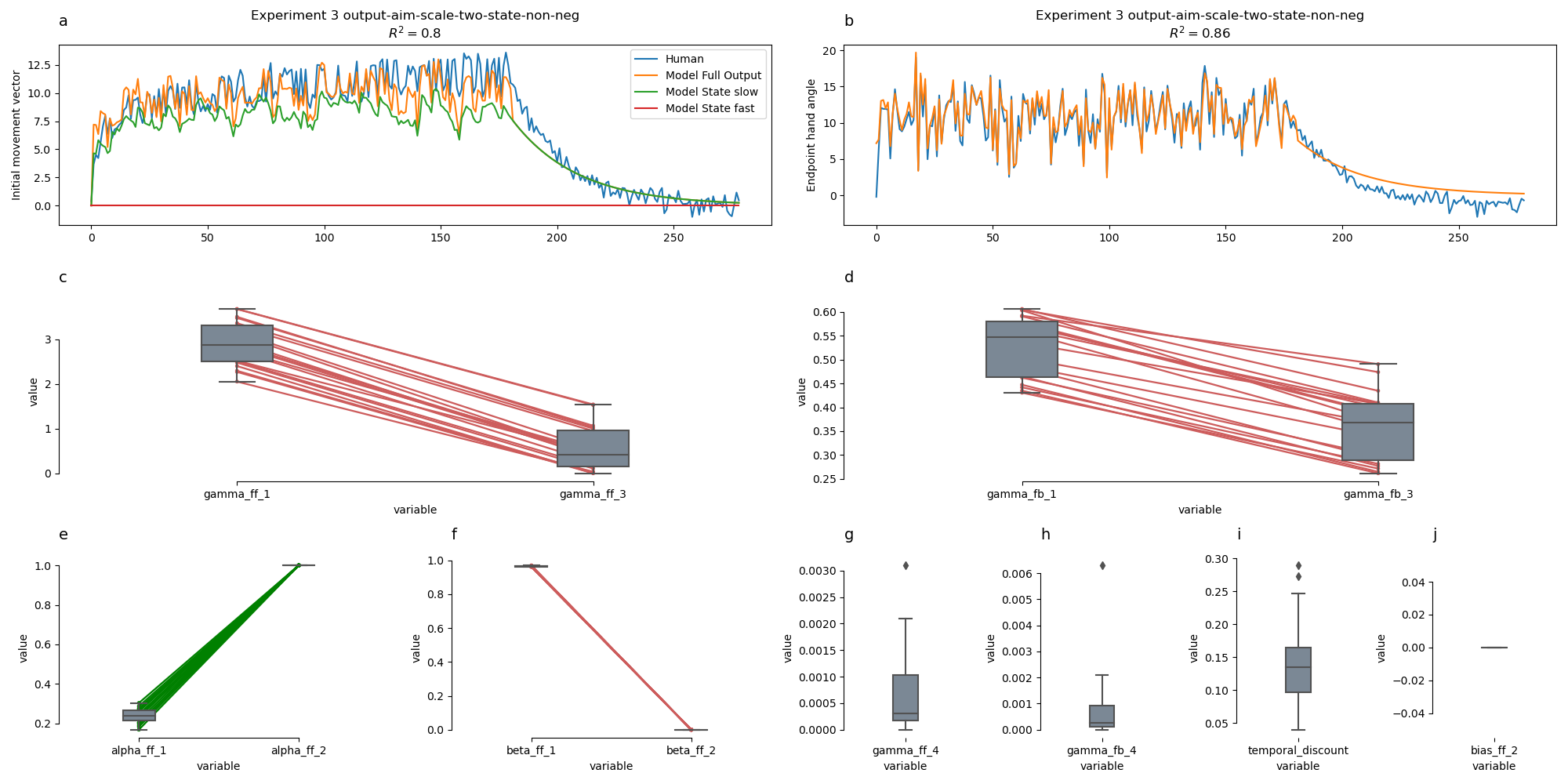

Supplement: S28 Fig — (TIF) [file pcbi.1010526.s031.tif]

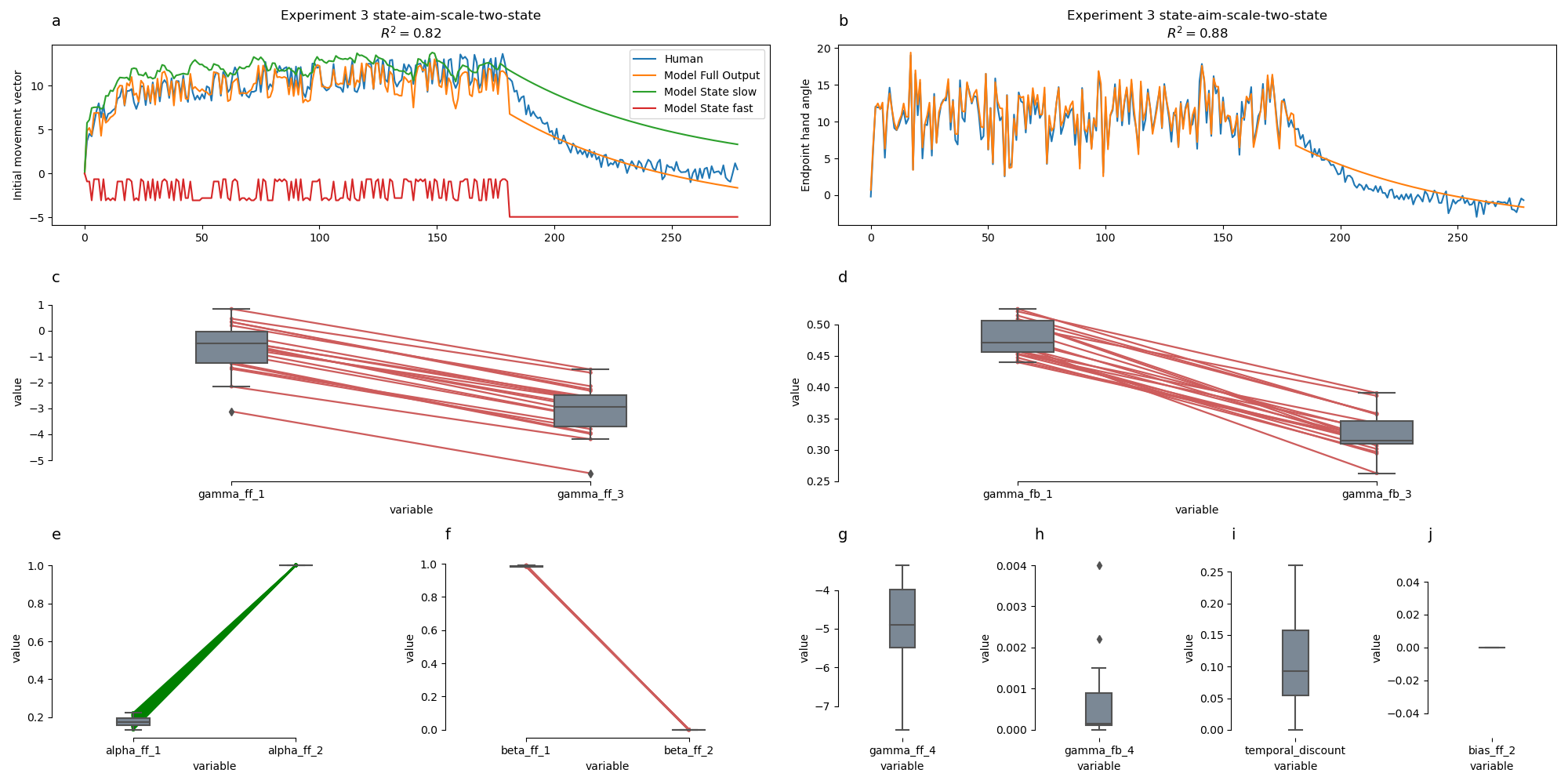

Supplement: S29 Fig — (TIF) [file pcbi.1010526.s032.tif]

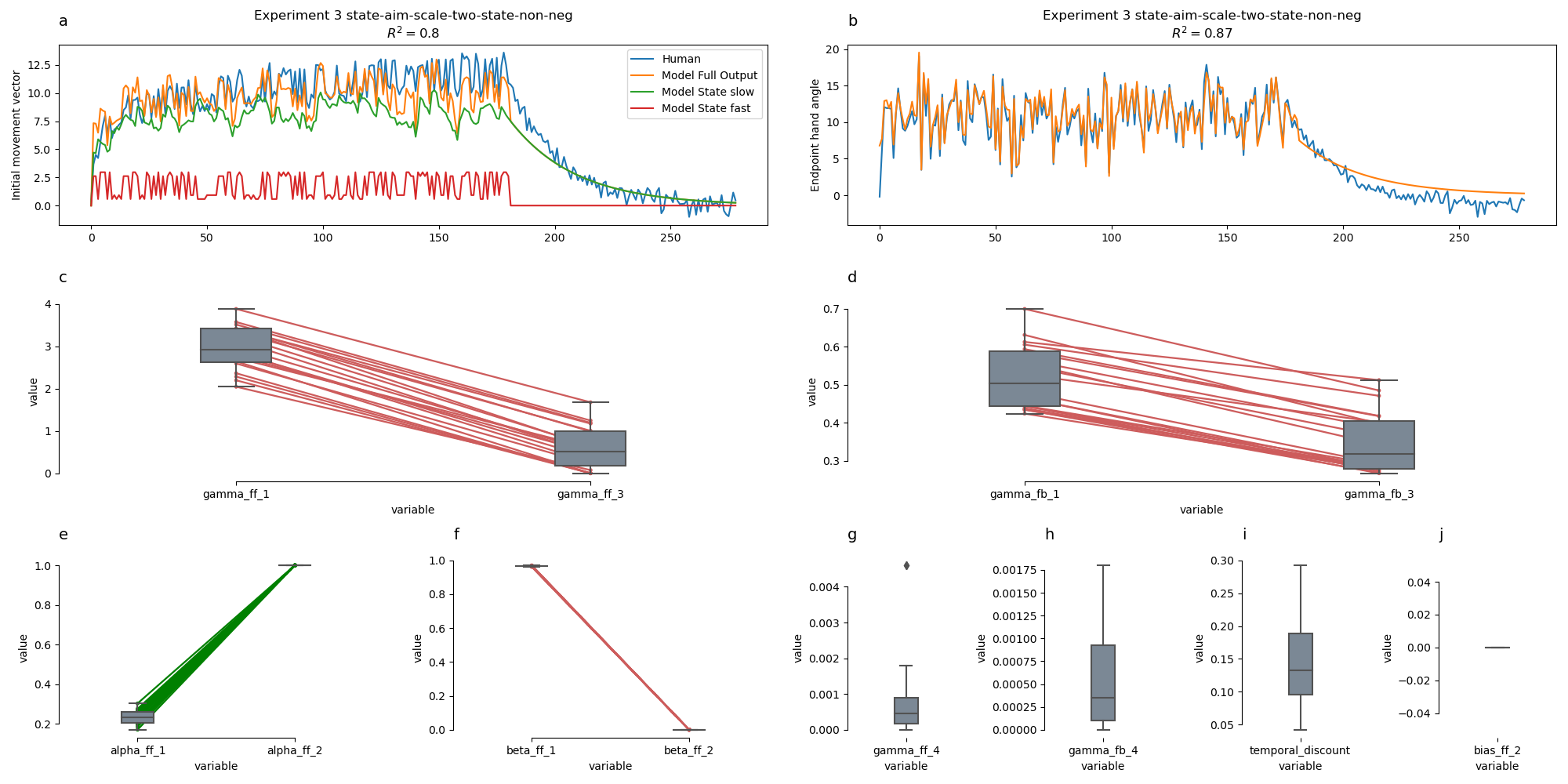

Supplement: S30 Fig — (TIF) [file pcbi.1010526.s033.tif]
