# Supplementary material for: Error-independent effect of sensory uncertainty on motor learning when both feedforward and feedback control processes are engaged
Source: PLoS Comput Biol. 2023 Sep 8;19(9):e1010526. doi: 10.1371/journal.pcbi.1010526 (PMC10522034; doi:10.1371/journal.pcbi.1010526)
Supplement: Attachment — Submitted filename: response_to_reviews_ploscompbio.pdf [file pcbi.1010526.s035.pdf]

We sincerely thank all reviewers for their insightful comments. We have made every attempt to address these comments and think this effort has lead to substantial improvements in our manuscript.

1 Reviewer 1

1.1 Reviewer comment

Here the authors test how uncertainty about feedback modulates feedforward adaptation (and also feedback commands). They conduct experiments where participants experience a visuomotor rotation, with visual feedback provided at the reach midpoint and/or endpoint. Their most striking finding (consistent across 3 experiments) is that when trials transition from low uncertainty to high uncertainty, there is a transient increase in adaptation, which is subsequently lost in an "anti-adaptive" fashion. This phenomenon could not be captured with a single-state model, suggesting that this behavior is due to the interplay between at least two states. The authors observe that two states models account for behavior with a gradually developing envelope captured by a slow learning state, and a fast state that has transient increases near low-uncertainty trials, but otherwise, remains close to zero. This behavior is achieved in the fast-state, via a context-dependent scaling in either error-based learning, retention, or state bias. The authors observed that the latter two scaling models best described their data.

Author response

This is an accurate summary of our results, and we thank the reviewer for their careful attention.

Major Concerns

1.2 Reviewer comment

Overall, I think this paper is very interesting. The effect of zero or low-uncertainty conditions transiently boosting adaptation seems very consistent across Experiments 1-3. However, there are various concerns I have about model behavior, model structure, overfitting, and interpretation. I think the authors could strengthen their paper by addressing these concerns.

Author response

We thank the reviewer for their generous evaluation of our paper. We also hope that our revisions detailed below satisfy their concerns.

1.3 Reviewer comment

Point 1: Why are no error bars provided in the primary figures? These should be added.

Author response

We have added error bars to all the relevant results figures.

1.4 Reviewer comment

Point 2: I am very confused about the "bias" term that is included in the model. The authors explain (Line 556) that this bias term was intended to "determine the baseline mapping that will be returned to in the absence of sensory input". But this does not appear to be the case. The bias term, λ, is placed in the state update equation:

$$x(n+1) = \beta x(n) + \alpha\delta(n) + \lambda$$

As such, λ, behaves as a bias not in the baseline mapping, but in the state-update process. I would be more inclined to describe this as drift. The equilibrium state of this model in the absence of feedback, is not λ. A proper model in which reach angle decays to λ, would have a bias perhaps in the process of motor production, not in the state update process:

$$y(n) = x(n) + \lambda + r(n)$$

Where $x(n) + \lambda$ is the overall motor command, and r is the perturbation. This model would indeed decay to $y(n) = \lambda$, as x decays to 0 in the absence of feedback (and perturbation).

As such, with this λ term functioning as a drift in the state-update process, I find it very difficult to interpret the behavior of these models. More importantly, including λ as a drift term in the state update process, would in many circumstances lead to unphysiological behavior. Let us suppose $\lambda = 2$ (what is intended to be a 2 deg bias in reach angle) and that $\beta = 0.99$, and $\alpha = 0.1$. Setting $y*$ to 0 and $r = 0$ (no rotation) yields the following behavior over 100 trials (note, in these simulations, the "scaling" models were not used, solely Eqs. (1)-(3) in the paper).

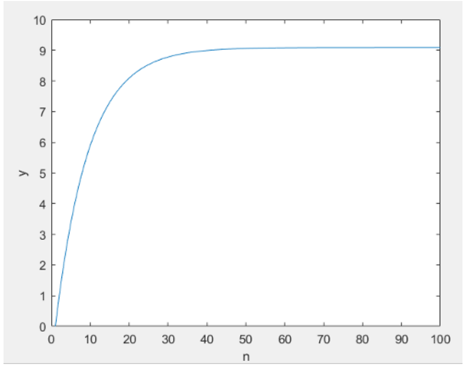

As you can see, even though there is no perturbation, and the intended goal is to reach to a $y*$ of 0, the model drifts to about 9 deg (rather than a bias of 2 deg). Thus, it is as if the participant is adapting to a rotation that does not exist. Similarly, let us re-run this simulation, but after trial 100, remove error entirely (so all that remains is $x(n+1) = \beta x(n) + \lambda$, because there is no error feedback (treating $\delta(n)$ as 0).

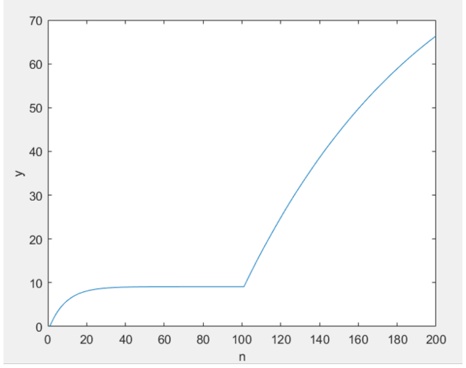

Here we can see even more strange model behavior. After the initial steady-state at 9 deg, removing error-feedback at trial 100 (setting the error term to 0, because it is not observed) causes an acceleration in the drift (because now there is no error-based learning process to counteract the drift). There is no physiological basis for these drifts.

At any rate, I hope these toy simulations are helpful in explaining why I think there is an error in the current computational models. This significantly impedes my ability to interpret model behavior and their comparison. While model parameters are not reported as far as I can see in the paper, I would imagine that the way the models avoid this pathologic behavior, is by estimating λ to be very small. This seems the only way that simulated behavior would not be plagued by the issues above.

The only exception to this rule, is cases where retention factors are 0. For example, in the single state fits in Experiment 1, the authors state that retention

parameters were estimated to be 0 (e.g., Figs. 4A-C). When the retention parameter is 0 (not physiological) then indeed, the bias term λ, will simply act to fix the internal state to an unchanging value. But in physiological scenarios where the retention parameter > 0, the bias term λ appearing in the state-update creates issues, as shown in the toy simulations above.

I think these bias terms are currently very problematic in the two-state model simulations, as they appear to lead to a strange behavior in which the fast state decays toward "negative" values (see Point 4 for more details on this). I do not think this unphysiological behavior should be allowed (instead of λ bounded between -10 and 10, it should be positive, if it is assumed the bias is acting on states, as opposed to the overall motor command). On a similar note, could the authors explain the foundation for these bias terms? While I am familiar with the idea that motor execution can be biased (i.e., sometimes a baseline bias in movement is subtracted from data to remove biases in motor commands) this does not seem to be a bias related to the adaptive processes, but rather to the production of movement even when no adaptation has taken place.

Author response

First off, thank you for taking the time to think so deeply about our paper and for offering your insights.

One thing you expressed concern about is the placement of a bias term in the state-update equation rather than on the motor output equation. Although we agree that placing the bias term here can seem counter-intuitive, one of the first, foundational papers to apply this sort of modelling to reaching behaviour provides a lengthy exposition on why doing so is preferred over placing the bias on the motor output [1]. One key thing to notice is that – while you are correct that the value of a bias term placed in the state-update equation will not be the observed bias in motor output – it will nonetheless ultimately *determine* what that bias will end up being. In essence, as you point out, the bias in the state-update will cause drift, but this drift induces errors that the system adapts to until all forces reach equilibrium. As such, we believe it is fair to refer to this term as a bias term, which again simply follows the seminal paper in the field [1].

Another concern is whether or not the behaviour of our two-state models is not "physiological" or is otherwise "pathological". First, we agree that having negative internal state variable values is quite unusual. However, we do not agree that this is necessarily a problem. First, this level of mathematical modelling is not intended to be directly or unambiguously tied to physiology, and so the model itself is by its very nature non-physiologic. We follow many others in the field in thinking state-space models of adaptation are descriptive or phenomenological models, not mechanistic models (See Haith & Krakauer 2013 [2], page 21, for further discussion). Without further evidence, the connection to physiology is relatively thin: at most it can be asserted that whatever the relevant brain

processes or circuits implement, they must drive a behavioral process similar to those encapsulated by our models. However, even with this caveat, we agree that it is worthwhile to consider the case where these models are not allowed to take negative internal state values. We now report the results from a class of models that satisfies this restriction.

Another element of your concern is that we do not provide adequate information for the reader to really figure out how the models are doing what they are doing. We now provide a lengthy supplemental materials section with figures that show the mechanics of each model with great detail.

1.5 Reviewer comment

Point 3: The R2 values, shown for example in Figs. 4D-F, for the two-state model fits are very high: higher than I would anticipate (other evidence for this would be the very high ω^2 reported with ANOVA statistics). This raises the concern of overfitting.

If I understand correctly, a two-state model currently has 2 retention parameters, 2 error sensitivity parameters, a bias term, 4 uncertainty parameters, a temporal discounting parameter, for a total of 10 parameters. This is a lot of free parameters, especially considering that the 4 uncertainty parameters, can exert their influence independently on a trial-by-trial basis (they never all act at the same time, given multiplication by the I matrix).

The possibility of overfitting is even stronger for the endpoint hand angle simulations, as seen for example in Figs. 4J-L. Here the R2 values are nearly 1. This seems unreasonably high to me and is highly suggestive that the high parameter count / model complexity is overfitting noise in the data.

On this note, while fits are provided to the average timecourse, trial-to-trial noise in movement execution/process noise, will still be present even at n=20. Given the random nature of these Gaussian noise sources, it should not be possible to attain R2 near 1, unless the data are overfit.

One way to check whether overfitting is indeed an issue, would be to fit models to individual participants and then calculate/report the R2 statistic. For individual participants, trial-to-trial noise should be a dominant feature in the data. R2 values for individual participants should be low. If the R2 values are high even at the level of fitting individual participants, this again would show that there is significant overfitting of the data.

While I am not sure, I suspect that a major contributor to overfitting is the nature of the model, with the 4 uncertainty terms. These terms will exert a strong trial-by-trial effect, that is independent for each of the 4 uncertainty trial types. I think this way of modeling uncertainty effects gives too much power to the models to fit unique “blips” in the data (be they positive or negative spikes) that can be significantly influenced by noise.

Author response

All models are fit to individual participants and used to generate predicted performance for each participant. These predictions are then averaged and overlaid with the mean human behaviour in all model prediction figures. The R^2 values reported in our original submission were very high because it was the value obtained by comparing just these two average curves. In our revised manuscript, we report the average of all individual R^2 values, which are indeed smaller.

Though the R^2 values are smaller, and likely agree more with your intuitions, there are further compelling reasons to believe that overfitting is not a problem for our analysis.

First, the model is being fit to the midpoint and endpoint observations across 280 trials. That means that the less than 20 model parameters are being asked to fit 560 data points. We were unable to find any resource on model diagnostics for which this amount of data was not considered much more than sufficient constraint for our situation.

Second, we don't think that fitting unique "blips" is a weakness of our approach, but rather we view it as a strength. That is, the insight should be how suprising it is that across 180 trials of adaptation only parameter numbers are needed to capture what would otherwise seem like wild volatility in performance.

We note here again that the models are fit to individual participants. This is what is so remarkable about the pattern in our data. Every participant across every trial responds to the sensory uncertainty in a very consistent and yet remarkably counter-intuitive way. This is the only reason why only 4 parameters can do so much work.

Finally, we performed an analysis in which we optimised the model parameters over only a random sample of adaptation trials and computed the model performance on the left out trials. Model performance was slightly worse than when optimised on all data but only negligibly. We have elected to leave this analysis out of the manuscript due to space concerns, but we are happy to add it if the editor feels it is worth the space.

1.6 Reviewer comment

Point 4: There appears to be something amiss in model behavior: the "negative" steady-state "equilibria" seen in the fast state in the 2-state model fits, despite perturbations having a "positive" mean value.

In the original two-state model paper by Smith et al., 2006, the fast state does indeed achieve "negative" values, but this is only in the case of a spontaneous recovery experiment, where the perturbation switches to a negative value, and the fast state rapidly approaches that value, thus becoming negative. No such

condition occurs in the present study, with the perturbations being on average "positive", and then entirely removed with no error during the washout period.

The idea that there is a fast state that attains a negative bias, despite the fact that the perturbation is "positive" and remains at this negative bias even during the washout phase seems to me highly suspect and unphysiological. This strange behavior is exemplified in Figs. 6F/8F. Here the slow state obtains a value that is larger than the overall behavior, and the fast state obtains a negative value, and ultimately a bias of about -10 deg during the washout phase. I have never seen this behavior reported in a two-state model, and this seems to me to be a function of unphysiological models overfitting the data.

I think this behavior is the symptom of at least two concerns, that I have noted: (1) that the data are likely being overfit, and (2) that the bias term λ, can produce unphysiological behavior when included in the state update term. Given the high model complexity I cannot be sure, but my guess is that these negative steady-states, are due to bias terms becoming negative. I would not consider these fast states becoming negative as a "feature" of the models used in this paper, but rather a "bug" that should be carefully examined / prevented.

Author response

Thank you again for your careful consideration of our data and methods. We hope you will agree that we have addressed the concerns expressed in this comment in our responses to your previous two comments.

1.7 Reviewer comment

Point 5: Model parameters should be reported somewhere, at the very least in a supplemental item.

Author response

Model parameters are now depicted in the Supplemental materials.

1.8 Reviewer comment

Point 6: The data provided in Fig. 3 is informative, and the text on Lines 133-144 is easy to follow. While it does appear visually that sensory uncertainty has a strong effect on feedforward adaptation, and perturbation size less clear, these claims should be validated quantitatively through some empirical approach. I would recommend calculating trial-by-trial change in initial movement vector. I would then compare these trial-by-trial changes across bins for each uncertainty level. Trial-by-trial change in angle could also be plotted against perturbation size in a continuous fashion.

Two notes here, however. First, it is not the case that perturbation size should drive differences in trial-by-trial adaptation (as implied in Fig. 3B). Rather, it is

error that drives adaptation. And so, I would suggest the authors calculate trial-by-trial error. They could then calculate trial-by-trial change in angle against error size. To depict this visually, they could bin this relationship based on error size.

The second note is that it seems these claims could be tested statistically. One issue is that comparing trial-by-trial changes in angle based on perturbation/error size, will be confounded by changes in sensory uncertainty (which clearly, at least in the 0 uncertainty condition, has a strong effect on behavior). Thus, I might suggest the authors perform some sort of multivariate regression, the outcome being trial-to-trial change in reach angle, and the independent variables being (1) error/perturbation size and (2) sensory uncertainty. They should look to see whether error/perturbation size, and/or sensory uncertainty have a statistically significant effect on trial-by-trial change in reach angle.

Similar to the point above, empirical analysis could also be done to support the claims made on Lines 145-155. Here the authors describe an anti-adaptive trend that happens when trial uncertainty increases between two trials. The authors can calculate the trial-by-trial change in reach angle, and bin it based on each transition in uncertainty level. Again, it seems that these responses could then be compared statistically.

The authors should also consider that this is not about a transition in uncertainty per se, but simply the previous trial's uncertainty. For example, in Fig. 3A, on approximately trial 80, there is a sequence of 3 trials that have zero-uncertainty, all which lie in the "higher" adaptation envelope. This seems to suggest that it is really only the last trial's uncertainty that leads to the upper/lower envelope, not a transition between uncertainty levels per se.

All in all, it would seem to me that there is additional empirical analysis of the data that the authors could conduct to better explore their data and clarify their model results. Wherever possible, conclusions about behavior should be drawn from an empirical analysis of the data, as opposed to simulations.

Author response

Thank you again for your careful consideration. We have completely revised our analysis approach to include both traditional statistical analysis and state-space modelling of our data. We have also revised our results section accordingly. In particular, we report regression analyses that are very much aligned with what you describe above. Given that this changes so much of the way the results section reads, we will not describe it further here. Please consult the manuscript for details.

1.9 Reviewer comment

Point 7: I think the discussion about implicit and explicit systems largely explaining the current results is relatively spot on (see Comment 8). I agree that

these experiments are not designed to really test implicit and explicit learning. But if there is a strong suspicion that an interplay between implicit and explicit learning is the root cause of central phenomena in the current data, it seems incumbent upon the authors to evaluate this possibility, with another experiment if need be.

Author response

While we agree with the reviewer that further experiments to probe the role of explicit strategies would be worthwhile, we think that the current paper contains sufficient experimental and analytical work to warrant publication without additional experiments. As it currently stands, our paper has 18 figures, 10 tables, and $\sim$ 12016 words (according to the latex code). We are deeply worried for our reader at the prospects of including even more experimental data and associated analysis and think that separate, follow-up publications would be a preferable approach. Furthermore, our results are completely novel and represent a significant jump in knowledge regardless of which – if either – hypothesis turns out to be correct. Second, there is simply no guarantee any study we run to resolve this mystery will turn out with a clear outcome. In our experience converging on useful and clear experiment designs is a slow and painstaking process. For these two reason we feel that bringing this mystery to light is a very important and noteworthy contribution to the field, and resolving the mystery will be best left to future research.

1.10 Reviewer comment

Observations that suggest an explicit process:

A. Consider the behaviors in Figs. 3A, 5A, and 7A. Here, no uncertainty trials (either in midpoint, or endpoint feedback) show a very strong transient boost in adaptation. I think this is highly suspicious (as acknowledged by the authors) for an explicit process. When feedback is unambiguous, participants engage a strategy on the next trial to counter the error. When feedback is uncertain, this explicit process is not used on the next trial. This to me seems a much more reasonable explanation for the “anti-adaptive” behavior.

Author response

Thank you very much for the detailed comments regarding the possible interplay between implicit and explicit processes. We agree that an explicit strategy may be at play here. The term “anti-adaptive” was used here as a descriptive term for the observed behavior. It was not intended as an explanation. However, we concede that this description brings with it some unwanted baggage. We have therefore removed all references to this term.

1.11 Reviewer comment

B. Note that sometimes no feedback trials produce the transient increase in learning in a sporadic manner (this can be seen with the "bumps" upward in Fig. 3A and 5A). While I do not have a clear explanation why this should be the case, it would appear that participants do occasionally choose large aiming angles after a no feedback trial. This transient increase after no error at all, seems to indicate the activity of an intentional process that can be turned on and off (on a side note, by analyzing their data further in an empirical fashion, they can detect how specific "jumps" to the higher envelope are to low-uncertainty conditions).

Author response

Thank you for pointing out this interesting feature. It turns out that all no-feedback trials that produce a large initial movement vector on the subsequent trial were themselves preceded by a low-uncertainty trial, suggesting that no-feedback trials may preserve the behavior from the previous trial. This is now briefly discussed in the results section from Experiment 1 and 2.

1.12 Reviewer comment

C. The authors state that during washout, participants were not given feedback, and were instructed to reach straight to the target. These are conditions that are used to assess implicit learning. Interestingly, in all 3 experiments, it appears that there is a discrete jump to the "lower adaptation envelope" at the start of each of these washout periods. This would seem to suggest that the lower adaptation envelope is capturing an implicit process that is revealed during the washout period (via instructions). The "jump" to the upper transient envelope that is abruptly "turned off" during washout, would be exactly consistent with an explicit, intentional process.

Author response

We now include statistics in the Results section comparing the hand angles during the last 10 trials of adaptation and the first 10 trials across the uncertainty conditions. In all three experiments, these statistics indicate that decay during no-feedback washout started from a state of high uncertainty. We also note in the associated text that such behaviour may be consistent with an explicit process but argue that such a link is not unambiguous.

1.13 Reviewer comment

D. The models roughly converge to the idea that the primary response to uncertainty is mediated by two qualitatively different states. The slow state accomplishes the lower adaptation envelope. The fast state accomplishes the transient "boosts" in the data to the higher envelope. With the interpretation that the

slow state largely captures implicit learning, and the fast state largely captures explicit processes (see McDougle et al., 2015), this again would be consistent with the idea that the apparent anti-adaptive behavior, is simply due to a fast explicit state that switches between two levels based on context.

I think that the possibility that the results are highly influenced by implicit/explicit processes is really critical, as it would completely change the authors' classification of low to high uncertainty trials as "anti-adaptive". Rather than the strange transient increases following zero-uncertainty trials be described as anti-adaptive, these could be ascribed to an intentional explicit process that responds in a context-dependent manner to uncertainty level. This would therefore not call for an overhaul of how error-based learning is typically modeled, but rather the inclusion of previous unknown context-dependent effects for the explicit system.

It seems to me that the authors really should attempt to rule out this implicit/explicit hypothesis with additional analysis, or ideally with an additional experiment. There are many that could be performed, but one simple control would be a repeat of Exp. 1, but with an instruction to reach straight to the target after some zero-uncertainty trials. In other words, the participants should be introduced to the idea about reaching straight to the target without feedback. On some trials following a zero-uncertainty trial, participants should be told not to aim, but to move straight to the target. On other trials, this should not be done. If there is a sensitivity to instruction in the response, this would indicate that explicit processes are at play in the current data. If indeed this is done, I might suggest to the authors to not simply do one aftereffect effect trial at a time, but 2 or 3, to make sure that participants do indeed listen to the instructions to stop aiming.

Author response

We agree that this interpretation of the data is highly plausible. Indeed, this was the original motivation for the "Adaptation versus aiming" subsection in the Discussion. However, regarding the inclusion of an additional experimental data set, while we agree that this is required to directly adjudicate between the implicit/explicit hypotheses, we do not agree that reporting such experiments in this paper ought to be necessary for publication. Please see our response to comment 1.13

1.14 Reviewer comment

Point 8: I really like the Discussion in the "Adaptation versus aiming" section and am inclined to mostly agree here (see Point 7 above). There is however, one statement I take some issue with, and this is the notion of a clear distinction between SPEs driving implicit learning, and performance errors driving explicit strategy. This indeed has been a strongly held belief in the field, but many authors now have observed that performance errors play a strong role in driving

both types of learning (see for example Miyamoto et al., 2020 Nat Neuro, Albert et al., 2022 eLife, Ranjan and Smith, 2020, Advances in Motor Learning and Motor Control).

I don't follow the idea that an SPE (which is midpoint or endpoint) that drives implicit learning, and a performance error (endpoint) that drives explicit learning, could account for their results. Experiments 1-3 are similar in overall subject behavioral trends, but in Experiment 1, only midpoint feedback is given. Here, the implicit and explicit systems would have to learn from the same error source (the midpoint error). Thus, I'm not sure this is about implicit/explicit adapting to different error sources, but rather the system responding to the same error source in qualitatively different ways.

In any case, it should be noted that SPE versus performance errors are generally not distinguishable. There are only 2 studies which really clearly disentangled SPEs and performance error: Mazzoni and Krakauer, 2006 [3] and Taylor and Ivry, 2011 [4]. The latter study clarifies the former and shows that visual target errors (which would not be considered an SPE per se) strongly modify the implicit response, again casting significant doubt that performance/target related phenomena do not modulate implicit learning.

All in all, I do not see why the assumption that implicit and explicit learning from separate error sources is needed to explain the data. On a general level, the notion that the slow envelope is implicit, and the fast and fleeting uncertainty-tuned corrections are explicit, does not seem to rely on these assumptions.

Author response

We agree with this assessment and have removed the unclear text regarding SPE vs performance errors in our discussion.

1.15 Reviewer comment

Point 9: One thing I wonder is whether the authors should include different classes of models. Here, uncertainty effects on feedforward adaptation are achieved by a context-dependent scaling of either state-update bias, error-based learning, or state retention. I think the authors should add other model types, that achieve uncertainty effects in a different way: e.g., an additive process. For example, a model could look something like:

$$x_s(n+1) = \beta_s x_s(n) + \alpha_s \delta(n)$$
$$x_f(n+1) = \beta_f x_f(n) + \alpha_f \delta(n) + \epsilon(n)$$

Here ϵ is a context-dependent term that assumes different values depending on uncertainty. Even simpler models than this could be included in the model

comparison, one in which the fast state behaves like an intentional explicit system, and simply turns on/off to 1 of 3 values depending on uncertainty:

$$x_f(n+1) = \epsilon(n)$$

Here x_f will simply be set to 1 of 3 values, presumably 0 when trial n is no feedback or high uncertainty (no explicit process is used after this), a medium value for a low-uncertainty trial, and a high value for a zero-uncertainty trial.

I think trying a model like this will reduce model complexity (and thus overfitting), while potentially capturing the main features of the data. My secondary hope is that additive models (in which the issue in the bias term noted in Point 2 is fixed) will not show the pathologic "negative" fast state behavior noted in Point 4. Overall, I think the authors could try ways to reduce their model complexity, while still capturing the main features in the data.

Author response

Thank you very much for the useful suggestions. First, please note that as far as we can tell, the model you wrote out at the top of this comment is equivalent to a bias-scaling model (or state-bias-scaling model in your terms), so that model class was already represented in our model-based analyses. However, your suggestion to use even simpler models (e.g., without retention or error terms) was very useful. We fit two such models (see the state-aim-scaling model and the output-aim-scaling model from the main text). In fact, these simpler models provide the most parsimonious explanation of our data by our new analyses.

Minor Concerns

1.16 Reviewer comment

Point 10: Be careful with language on Line 148-149. Yes, on average trial-by-trial changes should be in the positive direction. But even if uncertainty level never changed, there is motor/process noise that would cause the adaptation level to not increase monotonically on each trial. Secondly, when participants reach asymptotic behavior (steady-state), then on average there should be an equal number of trials where reach angle increases and reach angle decreases.

Author response

We have included a comment in this section stating that while motor noise may account for some of these "anti-adaptive" movements, the correlation between the level of uncertainty and movement direction on a given trial is statistically consistent across all subjects.

1.17 Reviewer comment

Point 11: Be careful with language on Lines 193-194. Yes, it is the case that the state and bias scaling models provide a better fit to the data than the error-scaling model. But that does not necessarily mean that feedforward adaptation is not well captured by the error-scaling model. Note again that the $R^2 2$ values, e.g., $R^2 = 0.84$ in Fig. 4D, are exceptionally high, and indicate that each model provides an excellent fit. It is really more a function here of choosing between 3 models that provide extraordinarily good fits to behavior.

Author response

We completely agree with this comment and have revised all aspects of the manuscript dealing with model comparison to call readers' attention to this important point.

1.18 Reviewer comment

Point 12: I'm not exactly sure where the conclusion is coming from in Lines 206-208, that feedback uncertainty does not inversely scale the magnitude of error-driven feedforward update. Is this related to the comment on Lines 193-194, that the error-scaling model does not do as well at fitting the data? If so, again I would be very cautious here, because all the models provide excellent fits to behavior. Because the error-scaling model isn't as good as the other models, should not be taken in a strong form to mean that Exp. 1 disproves the idea that error uncertainty does not inversely scale trial-by-trial error-based learning magnitudes. It seems this claim could be evaluated empirically in the data (see Point 6 above).

Author response

In our original submission, this claim was indeed intended to be supported by our modelling results, but we agree that doing so was not as compelling a source of evidence as it could have been. Our revised manuscript augments our previous model-based analysis with more traditional statistical analysis. Together, these complementary analysis approaches put our main conclusions on much firmer ground. In essence, we can now base this claim on the finding that in a regression model the interaction terms between sensory uncertainty and error are either non-significant or else are not significant in a pattern that is consistent with error scaling. Please see the updated methods and results for details.

1.19 Reviewer comment

Point 13: I'm not following the narrative on Lines 213-216, where the authors bring up "temporal proximity of the sensory feedback signal to movement offset". My confusion arises because the authors bring this up as what appears to be an alternate hypothesis for their results in Exp. 1. But all participants and

all trials had feedback only at movement midpoint, and the effect of temporal proximity of feedback to movement offset was not tested yet. So, I am a little confused where this line of thought/exposition is coming from.

Author response

As subjects were instructed to reach to the end target on each trial, we interpret the end of this movement as "movement offset", which is temporally dissociated from the midpoint feedback. Accordingly, we raise the possibility that the disparity in feedback timing (at midpoint) and movement offset may effect adaptation, relative to a context in which feedback is synchronously provided with movement offset. Hence we raise the possibility of a temporal effect, which requires Exp2/3 to compare.

1.20 Reviewer comment

Point 14: Typo on Line 393: the experiment number is not indicated here.

Author response

Line 393: Corrected. Thank you.

1.21 Reviewer comment

Point 15: Low uncertainty (orange) trials seem to cause a moderate increase in adapted reach angle on the next trial (see Figs. 3A and 5A). This may have two causes. A true intermediate boost in adaptation. Or a large, but improbable boost in adaptation. To explain what I mean, it could be that on no-uncertainty trials, adaptation boosts by 3 degrees on the next trial only, 100% of the time. But on low-uncertainty trials, adaptation boosts by 3 degrees on the next trial, but only 20% of the time. The rest of the time there is little to no boost. Alternatively, low-uncertainty trials might boost adaptation by 0.6 degrees, 100% of the time. I would be interested in the authors examining the histogram of the trial-by-trial change in reach angle on the low-uncertainty and zero-uncertainty trials, to arbitrate between these possibilities.

Author response

See figures 5, 9 and 13 that depict violin plots – which contain the requested histograms – of change in initial movement vector across uncertainty conditions. To our eyes, these plots are more consistent with the latter option (small difference in change 100% of the time). In the interest of brevity, we do not comment on this possibility in the paper, but we are happy to do so if the editor feels that it is important.

2 Reviewer 2

2.1 Reviewer comment

The study investigates how visual uncertainty of movement feedback affects motor adaptation when the feedback is provided during and after the movement. Traditional motor adaptation studies on sensory uncertainty focus on how adaptation evolves between trials in a feedforward fashion. Instead, the paper studies a combined case when in-movement sensory integration and between-trial adaptation co-occur. With three behavioral experiments, the authors found that randomly presented high-uncertainty trials (implemented by modifying visual feedback during and after movements) lead to abrupt changes in feedforward adaptation, which are dependent on uncertainty level but not on movement error size. Model comparisons revealed that scaling the retention or trial-by-trial bias instead of the error adaptation rate can better explain the data. These findings are drastically different from those in the literature, where sensory uncertainty has been repetitively shown to affect the adaptation rate.

The study is original, with a strong focus on probing its study aim. It is also novel in combining online movement feedback integration and offline motor adaptation. The technical part of modeling is also sound. However, the seemingly surprising results and their explanations invite scrutiny as the task design probably invokes unwanted effects, making their conclusions thus far ungrounded.

Author response

We thank the reviewer for their thorough summary and greatly appreciate their suggested corrections.

Major Concerns

2.2 Reviewer comment

First, some important behavioral patterns and modeling results have not been fully analyzed or explained, preventing our thorough understanding of the interesting phenomena:

1. The main selling point is that the mid-movement feedback enables a feedback integration component in addition to the feedforward adaptation. However, the paper only analyzed the initial movement direction to study feedforward adaptation. Is there any proof that feedback integration happens during the movement? Do we see a curved movement trajectory or a change in movement endpoint? How does this potential behavioral correction relate to the model-fitting results? It is well possible that the so-called feedback integration is negligible. And the influence of mid-movement feedback should be considered as part of feedforward adaptation, which only affects the next trial. This is a vital question before we try to understand what these peculiar findings mean.

Author response

We thank the reviewer for this comment and opportunity to clarify our position. Although we do carefully analyze the impact of sensory uncertainty on feedback integration (see Figures 6, 10 and 14 and sections *Feedback Integration* in *Results*), it is certainly true that we emphasize the findings related to feedforward adaptation over those pertaining feedback integration. This is primarily because these results have been established for some time, especially with the seminal work of Kording & Wolpert (2004) [5]. Also see Hewitson et al. (2018) [6].

The revised manuscript now benefits from an overhauled and expanded results section. Please see Figures 6, 10, 14 which directly show feedback integration as a function of sensory uncertainty. This information is also visible in Figures 16, 17 and 18 in which initial movement vectors are shown in the left column and endpoint hand angles are shown in the right column. Attending to the y-axis scale make clear that feedback-integration does indeed occur.

2.3 Reviewer comment

2. A cross-experiment comparison is lacking. It is apparent that exp2 and 3 have larger adaptations than exp1. Do the three experiments have the same decay in the washout phase? How do the experiment differences inform the modeling? I see the slow state during the decay from all models is negative in all three experiments, up to -10 in exp3. This is rather surprising given the current understanding of the slow state, which probably reflects the implicit adaptation or the true re-calibration of the internal model. But it would never be negative. Note the decay portion of the data largely determines the model fitting.

Author response

In the revised manuscript, we have tried to even more explicitly signpost how the 3 experiments are related to one another and how they relate logically. Please see the beginnings of each experiment description in the results section for this signposting. We hope this will make it clear what our aims were in running this set of experiments rather than others. Although we do now explicitly draw comparisons in the text between the results of the 3 experiments, it was not our aim to provide an extensive cross-experiment analysis. Please see the revised manuscript for details.

2.4 Reviewer comment

3. The quick transitions between low and high uncertainty trials should be plotted better using other means, such as a scatter plot of uncertainty vs. movement direction (at the adaptation plateau). What do the adaptation changes look like

for each kind of transition? Currently, this part of vital results is not even visible.

Author response

These figures have been completely revised along with much of the rest of the manuscript. We feel that the new figures do precisely what the reviewer requests. See figs 3,7 and 11 in the new manuscript.

2.5 Reviewer comment

4. What does the transition from the last adaptation trial to the first washout trial look like? This is another vital piece of missing information to unpack what constitutes the adaptation here. If the authors give clear instructions to drop the re-aiming strategy at the beginning of the washout, we should expect a sudden decrease in the movement direction. And, then the second hypothesis mentioned in the Discussion (strategic learning leads to fast changes during adaptation) can be examined.

Author response

We have updated the methods section to clearly state what instructions were provided. In summary, the washout phase was not explicitly signalled to the participant. Nor was there any break between the baseline, adaptation or washout phase. Subjects were instructed to move their hand straight through the target as accurately as possible using whatever visual feedback they may or may not be provided on any given trial.

We have also included statistics in the Results section comparing the hand-angles during the last 10 trials of adaptation and the first 10 trials across the uncertainty conditions. In all three Experiments, these statistics indicate that decay during no-feedback washout started from a state of high uncertainty.

2.6 Reviewer comment

5. Why does the state-scaling model win over the bias-scaling model in exp2 and 3 while we see the opposite in exp1? Note that the overall learning patterns are similar across experiments. Is it possible that the retention term and the bias term co-vary to generate the effect?

Author response

This is an interesting question and it does seem possible that these terms covary in the manner suggested. Another plausible explanation is that all models fit the data overall pretty well, so our analysis might fall victim to local minima in the optimisation. In any case, the expanded modelling results should go some way to addressing this comment. But to summarize our general finding,

state-aim-scaling and output-aim-scaling almost always fit best. Importantly, the error-scaling model never fits best.

2.7 Reviewer comment

Second, though some of the behavioral data were not shown clearly in the paper, I think the task design has introduced some unwanted effects, which are not possibly modeled by the error-based model variants here.

The perturbations are random rotation angles, $\sim N(12, 16)$ degrees. The gaussian point clouds are specified in cm (the paper should convert one or another to make a clear description of what the perturbation would look like).

Author response

We have added arc-angle conversions for the various feedback types in the General Experimental Procedure section.

2.8 Reviewer comment

By my calculation, the mid-movement perturbation is, on average, centered at 1.06cm away from the desired straight line, and the endpoint perturbation is, on average, 2.12cm. The high-uncertainty dot cloud (with a SD of 1cm) would "touch" the straight line, thus giving people the impression that there is no perturbation (zero error). The medium-uncertainty dot cloud (with a SD of 0.5cm) would also touch the straight line if presented at the mid-movement. This is probably the reason that these trials lead to fast trial-by-trial changes, riding on top of a slow time-scale learning curve. These interleaved "target-hit" trials would affect strategic re-aiming and implicit adaptation. The former is driven by performance error (for the target hit, it is zero). The latter is also expected since, say, Richard Ivry group (Kim et al., 2019) have shown that touching the target, even barely without a direct hit, would effectively reduce the implicit adaptation. The mechanism for the damped implicit learning by this zero or reduced performance error (not sensory prediction error modeled here) is not clear, though reinforcement learning or motivational factors have been suggested.

In other words, the study started off by implementing cloud dots to manipulate sensory uncertainty (precision) but triggered unwanted learning mechanisms that depend on performance error (bias). This target hit would be more severe for mid-movement feedback, which is the study subject of the paper. Thus, it is not fair, as claimed by the paper, that the sensory uncertainty at the midpoint, as opposed to the perturbation size, determines the feedforward adaptation here. Or, as it claims in the title, "sensory uncertainty punctuates motor learning independently of movement error" It is the manipulation of the sensory uncertainty that accidentally nullifies the perceived error and thus leads to a temporary withholding of the adaptation process (note all these cloud-dot

trials are interleaved with the small-uncertainty trials, which I believe is clearly perceived off the straight line).

In this light, the so-called anti-adaptive effect, seen in angle changes between two cloud-dot trials (σ_L to σ_M, or σ_M to σ_H), is totally expected. Note the reduction of learning amounts to ¿ 30% after single mid-movement feedback. This kind of fast change is indeed not expected by any model incorporating the effect of sensory uncertainty but is expected with a combination of a fast strategic re-aiming and damped implicit learning. Given these extremely volatile changes, the single-state models will not work, and the error-scaling model will not work either, given the normal range of learning rate.

Author response

This is an interesting point but after careful consideration and analysis we do not believe it is a viable explanation for our data.

In particular, by our analysis for every experiment, every medium to high uncertainty trial is a hit and low uncertainty trials are mixed hit / miss. The reviewer's idea suggests that all the hits on the higher uncertainty trials cause diminished adaptation, and this is why they are several degrees lower than the low uncertainty trials. This explanation works well enough to explain why the first low uncertainty trial jumps above the others, but as soon as the adaptive state reaches that higher level, it offers no explanation for why it would decay so suddenly (i.e., on a single trial) back to the lower envelope. The key here is to notice that diminished adaptation due to a target hit is not the same thing as a reduction in adaptation. Whatever adaptation may occur – diminished as it may be – will always go against the error and should therefore increase hand angle, not decrease it.

A second thing suggested by this reviewer's comment is that the hit/miss mechanics aren't driving adaptation, but rather are driving an explicit strategy. But for this to work, we would have to contend that following hit trials, the participant deems their performance adequate and resolves to maintain whatever strategy they just employed. This explains why the there would be little to no stratification between the higher uncertainty trials, but falls apart when we consider the low uncertainty trials. About half of these trials are hit and half are miss, yet 100% of them are several degrees above the rest. So this clearly does not line up. Furthermore, there is no rationale here as to why – once a new strategy has been adopted owing to the experience of a miss trial – that strategy would be abandoned simply because of a hit trial on the next higher uncertainty trial.

In short, the idea that our results are driven by hit/miss mechanics leads to substantially more convoluted and perhaps a fundamentally unworkable account of our results than those we have put forward.

Minor Concerns

2.9 Reviewer comment

I don't understand why we can simply assume that the uncertainty only affects the fast state in the two-state variants of the models. I know that fast, abrupt adaptation changes are prominent in this data set (possibly due to the reasons I give above). But in the domain of motor adaptation, the slow process has been shown to be dependent on sensory uncertainty (e.g., an implicit adaptation that is supposedly governed by slow processes is affected by proprioceptive uncertainty). Theoretically, it is explicit learning (one of the fast processes) that is less affected by sensory uncertainty (precision) since the mean performance error (bias) matters more for strategical learning. Anyhow, in this sense, the results strongly suggest that the fast changes, which are uncertainty-dependent, reflect the explicit learning component.

Author response

Our assumptions were grounded on the visual observation of a slow envelope marked by fast uncertainty-dependent deviations. If we are reading this comment correctly, then we agree with the reviewer that this assumption was a reasonable one given our data.

2.10 Reviewer comment

Figures 3, 5, and 7 can use a different color coding to distinguish the perturbation size better while making the zero perturbation a completely different color off the color-coding scale.

Author response

We have rebuilt nearly all the figures and feel this issue is well addressed.

2.11 Reviewer comment

Line 614: $x_{fb}(n)$ denotes the so-called feedback gain, but why feedback gain follows a state-space model as specified by eq.15? Eq14: $y(n, t_M P)$ is unspecified. Note how this variable relates to eq.13 is also not given.

Author response

Evidence that feedback gain follows a state-space model can be found in the literature [7, 8, 9, 10, 11].

2.12 Reviewer comment

As it currently stands, the model is poorly described. It is better to give a graphic illustration of how the modeled variables are related in this movement paradigm. Simply providing a list of equations would not give the readers a gist of what is modeled, especially when the model consists of two time scales, two time points of updating, and two learning mechanisms (feedback and feedforward). To simplify the modeling part, I suggest only presenting the two-state model variants since they are well accepted as the default model for motor adaptation and also fit the data well. If possible, put the single-state model results in the supplementary and focus on comparing different scalings in the main text.

Author response

We have tried hard to write the methods as clearly as possible, and respectfully disagree that a graphical illustration is intrinsically better. The model is only as complicated as the behavioural paradigm demands. Given that our paradigm combines feedback forward adaptation and feedback integration, we feel that carefully reading through the equations and accompanying text is more than reasonable to ask of the technical readership of this journal.

2.13 Reviewer comment

Line632-635: eq.2, not 3?

Author response

The methods section has been heavily revised and we believe this is no longer an issue.

2.14 Reviewer comment

eq.17: Is the gamma parameter necessary? Why do we need to assume that the mid-point feedback and the endpoint feedback compete?

Author response

We do not not assume that midpoint and endpoint feedback compete. If they do not compete, then this parameter will be 0.5. If they do, then it will be otherwise. In essence, by including this parameter, we let the data determine whether or not they compete.

2.15 Reviewer comment

Instead of giving out ANOVA results (Line185) to show significance, the author should provide condition means to give us a full picture of how this effect changes across uncertainty conditions.

Author response

The results section has been completely overhauled to include basic empirical analyses including regression models. Here we also include condition means and SD as requested.

2.16 Reviewer comment

Figures fonts are too small to read.

Author response

We have remade nearly all figures and have included this change.

2.17 Reviewer comment

Line349: unclear to me what the authors are trying to convey here. “Two uncertainty conditions” instead of “two high uncertainty conditions”?

Line393: Experiment 3

Author response

Correction made: “Two uncertainty conditions” has been replaced with “two high uncertainty conditions”

Line393: Correction made: changed reference to “Experiment 3”

3 Reviewer 3

3.1 Reviewer comment

In this paper, Hewitson and colleagues investigate the effect of visual uncertainty on feedback and feedforward processes during motor adaptation. These influences had been studied separately for feedforward and feedback processes and they do it at the same time. The authors found that sensory uncertainty had the expected effect on feedback process (less correction when more uncertainty) but it had an unexpected effect on feedforward process (increasing uncertainty decreased adaptation). I found the results intriguing and interesting. It is a pity that the authors did not investigate further whether the observed pattern could be due to the explicit component of adaptation. I am very skeptical of the modelling approach and there is little rationale for the different modelling choices. These and other comments are developed below.

Author response

We thank the reviewer for their careful attention and hope to address their comments below.

Major Concerns

3.2 Reviewer comment

1. The authors discuss the idea that the changes due to sensory uncertainty might be due to the explicit component of adaptation. They left it opened without real conclusion because they did not actually test it. This is in my opinion detrimental to the paper because it left it without a real conclusion. The results now are not really interpretable because we don't know what the nature of the rapid changes is. I believe that this paper should test whether the explicit component of adaptation is responsible for the rapid shift or not. If it is due to explicit strategies, then it is not comparable to what happened to the feedback process which is likely implicit. If it is due to the explicit component of adaptation, then the model does not really make sense as explicit adaptation is driven by task performance error and not by sensory prediction error like the implicit component is. Yet, in current models in the paper, both states are driven by sensory prediction error.

Author response

We agree that additional experiments are required to adjudicate between implicit adaption versus explicit strategies in interpreting our data. However, we do not agree that completely resolving this mystery should be expected of any single paper, and we feel that our work stands on its own as a substantial and important contribution to the field. Please see our response to comment 1.9 for further details.

3.3 Reviewer comment

2. I have several problems with the model. The model seems to be ad-hoc and not really supported by previous research or experimental observations:

a. The authors seem to confound task performance error and sensory prediction error. Sensory prediction error drives the implicit adaptation while the task performance error (midpoint or endpoint) drives the explicit component of adaptation and feedback responses. The model mixes the two continuously.

Author response

Unfortunately, we are having trouble interpreting this comment. For instance, our models don't include the term "task performance error" anywhere. It only uses sensed error at midpoint and sensed error at endpoint. If the reviewer is referring to the passage from our discussion section that speculated about sensory prediction error and task performance error, then we hope the reviewer will be pleased that we have removed this passage.

3.4 Reviewer comment

b. The authors model the feedback gains as following a state-space model (Eq.15). Where is the evidence for such a model? Feedback gains can be changed within a trial (work of Fred Crevecoeur on small and large targets) and are tuned continuously in function of the sensory feedback. I don't think that the model would be able to capture that.

Author response

Evidence that feedback gain follows a state-space model can be found in the literature [7, 8, 9, 10, 11]. Also, thank you for the interesting note regarding how continuously tuned feedback gain can be. Ultimately, the work cited in this comment is simply not in the current scope of the model, so we cannot say with confidence whether it would or would not capture the results mentioned. Furthermore, we contend that it is largely irrelevant if it does or doesn't, because the major point of the model is to explicate some possible ways that feedback integration might alter how feedforward adaptation responds to uncertainty.

3.5 Reviewer comment

c. The model contains the idea that endpoint feedback is used to tune the gains on the next trial. Where is the evidence for that statement (Eq.16)? Eq.16 does not make sense to me. If it is a state-space, you need to update it once for midpoint and once for endpoint. Updating it based on two errors is weird.

Author response

By updating all state variables (feedforward and feedback) after movement completion, we are assuming that any update that may be initiated at movement midpoint takes much longer to be instantiated in neural circuits than the time required to finish the reach. This seems like a very plausible assumption to us.

3.6 Reviewer comment

d. Eq.16 also suggests that the sensory prediction error takes the feedback response into account. What is the evidence that this is actually the case?

Author response

This is a common assumption in models that join feedforward and feedback control [7, 8, 9, 10].

3.7 Reviewer comment

e. Eq.17 suggests that sensory feedback from midpoint and endpoint is weighted to compute a weighted average error. What is the evidence that this weighting

exists? In addition, I don't see how this would generalize to a condition where visual feedback is continuously available.

Author response

We are unaware of any such evidence for or against our weighted average approach. This is likely due to the fact that our paper is highly novel, so we are among the first to have a need to make such assumptions.

Generalising the model to a condition where visual feedback is continuously available may indeed be an interesting challenge, but doing so simply is not in the scope of our results and therefore not a critical objective for us to demonstrate in this paper.

3.8 Reviewer comment

f. I wonder where the parameter Lambda comes from. This bias-parameter is absent from previous versions of the two-state models and there is not real rationale to add it to the model. In addition, the authors decided to bound it [-10,10] without any further explanation.

Author response

Both the presence of a bias parameter and it's position in the state-update equations as opposed to the motor output equations comes from Cheng and Sabes (2006) [1], which is one of the seminal papers on using state-space models to model sensorimotor adaptation. Please see our response 1.4 for further details.

3.9 Reviewer comment

g. In Eq.18 and 19, the authors use a weighted some of sensory uncertainties (on retention rate and lambda). This seems weird to me and I wonder what the rationale for that would be. Do the authors think that the influence of sensory uncertainty on the retention rate from midpoint and endpoint feedback is simply the average of their sensory uncertainty? That does not make sense.

Author response

The rationale for this choice was simply that it is unclear what the net uncertainty of sensory feedback should be when midpoint and endpoint feedback are both present but with different sensory uncertainties. The weighted sum approach that we took seemed to be the most straight forward way to answer this question.

3.10 Reviewer comment

In other words, I don't think that the models are useful because they seem to have been built to specifically fit the results of the current experiments and will not be generalizable to any other experimental results.

Author response

Our models were built to model how feedforward adaptation and feedback integration respond to sensory uncertainty when they co-occur. As such they will naturally generate predictions in any experiment that examines adaptation with feedback at movement midpoint and/or endpoint. Standard state-space models in the field – from which ours inherit – cannot do this (i.e., they do not generalise to our data).

3.11 Reviewer comment

3. There is a lack of information about the quality of the fit for individual participants. If Fig.4,6 and 8, R2 are given for all participants together but the quality of the fit for individual participants are never given. Coltman and Gribble (https://pubmed.ncbi.nlm.nih.gov/30840553/) showed that fits of two-state models are not very reliable for single participants while they were at the group level. Could the authors provide more information about the quality of the fit for the individual participants and a sensitivity analysis of the obtained parameters?

Author response

All models are fit to individual participants and used to generate predicted performance for each participant. These predictions are then averaged and overlaid with the mean human behaviour in all model prediction figures. The R^2 values reported in our original submission were very high because it was the value obtained by comparing just these two average curves. In our revised manuscript, we report the average of all individual R^2 values, which are indeed smaller.

We also performed an analysis in which we optimised the model parameters over only a random sample of adaptation trials and computed the model performance on the left out trials. Model performance was slightly worse than when optimised on all data but only negligibly. We have elected to leave this analysis out of the manuscript due to space concerns, but we are happy to add it if the editor feels it is worth the space.

3.12 Reviewer comment

4. I found the title weird. There is no evidence in the paper that the sensory uncertainty does not punctuate motor learning. The slow system is not

interrupted by the sensory uncertainty. None of the parameters become zero or so. The fast system is simply very volatile but this is added on top of a slow component that is still learning.

Author response

We think the title is an accurate description of our main finding. In particular, we found that participants gradually adjust their reaching movements from trial-to-trial in a manner that is well characterised by a slow and consistent envelope of error reduction. Riding on top of this slow envelope, participants exhibit large and abrupt changes in their initial movement vectors that clearly correlate with the degree of sensory uncertainty present on the previous trial. However, these abrupt changes appear to "punctuate" or interrupt the underlying pattern of gradual error reduction, in the sense that they are insensitive to the magnitude and direction of the sensed movement error and can even sometimes serve to increase the error experienced from trial to trial. None-the-less, we have opted to change the title to hopefully more directly portray the main finding of the study.

Minor Concerns

3.13 Reviewer comment

1. Instructions delivered during the washout phase needs to be specified. It is written that the participants were asked to reach straight to the target but, despite this instruction, the pattern of after-effect looks very different than what others have reported with similar instructions (e.g. work of Taylor). It is also unclear how the participant made the difference between no-vision trials during the adaptation and during the washout. In other words, how was the washout period signaled to the participant.

Author response

We have updated the methods section to clearly state what instructions were provided. In summary, the washout phase was not explicitly signalled to the participant. Nor was there any break between the baseline, adaptation or washout phase. Subjects were instructed to move their hand straight through the target as accurately as possible using whatever visual feedback they may or may not be provided on any given trial.

We have also included statistics in the Results section comparing the hand-angles during the last 10 trials of adaptation and the first 10 trials across the uncertainty conditions. In all three Experiments, these statistics indicate that decay during no-feedback washout started from a state of high uncertainty.

3.14 Reviewer comment

2. There are several problems with the report of the statistics. There are many p "¿" 0.001, which should be "¡". Some statistics are weird. For instance, line 190-192, the statistics is wrongly reported. The p-value is p¡0.001 and not p=0.99. This effect size is actually huge. Please, check all the statistics of the paper. Maybe use StatCheck?

Author response

We apologise for these errors. The analyses and results have been completely revamped, and we have made every effort to ensure that no additional errors of this sort have been introduced.

3.15 Reviewer comment

3. The simulation with single state-space models is useless. From the experimental results, it is pretty clear that a model with a single state will never be able to reproduce such pattern.

Author response

We agree that this may be obvious to some readers, but also suspect that readers less familiar with state-space modelling as applied to motor learning may appreciate the slower and more complete discourse.

References

1 Cheng, S., & Sabes, P. N. (2006). Modeling Sensorimotor Learning with Linear Dynamical Systems. *Neural Computation*, *18*, 760–793.

2 Haith, A. M., & Krakauer, J. W. (2013). Theoretical models of motor control and motor learning. In *Routledge handbook of motor control and motor learning* (pp. 16–37). Routledge.

3 Mazzoni, P. (2006). An Implicit Plan Overrides an Explicit Strategy during Visuomotor Adaptation. *Journal of Neuroscience*, *26*(14), 3642–3645. https://doi.org/10.1523/JNEUROSCI.5317-05.2006

4 Taylor, J. A., & Ivry, R. B. (2011). Flexible Cognitive Strategies during Motor Learning (J. Diedrichsen, Ed.). *PLoS Computational Biology*, *7*(3), e1001096. https://doi.org/10.1371/journal.pcbi.1001096

5 Körding, K. P., & Wolpert, D. M. (2004). Bayesian integration in sensorimotor learning. *Nature*, *427*(6971), 244–247. https://doi.org/10.1038/nature02169

6 Hewitson, C. L., Sowman, P. F., & Kaplan, D. M. (2018). Interlimb Generalization of Learned Bayesian Visuomotor Prior Occurs in Extrinsic Coordinates. *Eneuro*, *5*(4)
Publisher: Society for Neuroscience.

7 Kawato, M. (1999). Internal models for motor control and trajectory planning. *Current Opinion in Neurobiology*, *9*(6), 718–727. https://doi.org/10.1016/S0959-4388(99)00028-8

8 Thoroughman, K. A., & Shadmehr, R. (1999). Electromyographic Correlates of Learning an Internal Model of Reaching Movements. *The Journal of Neuroscience*, *19*(19), 8573–8588. https://doi.org/10.1523/JNEUROSCI.19-19-08573.1999

9 White, O., & Diedrichsen, J. (2010). Responsibility assignment in redundant systems. *Current Biology*, *20*(14), 1290–1295
Publisher: Elsevier.

10 Ito, M. (2013). Error detection and representation in the olivo-cerebellar system. *Frontiers in neural circuits*, *7*, 1
Publisher: Frontiers.

11 Franklin, S., Wolpert, D. M., & Franklin, D. W. (2012). Visuomotor feedback gains upregulate during the learning of novel dynamics. *Journal of neurophysiology*, *108*(2), 467–478.
