# Supplementary material for: Error-independent effect of sensory uncertainty on motor learning when both feedforward and feedback control processes are engaged
Source: PLoS Comput Biol. 2023 Sep 8;19(9):e1010526. doi: 10.1371/journal.pcbi.1010526 (PMC10522034; doi:10.1371/journal.pcbi.1010526)
Supplement: Attachment — Submitted filename: response_to_reviews_ploscomp_bio_june_2023.pdf [file pcbi.1010526.s037.pdf]

We thank the reviewers for their continued patience and thoughtful feedback in the latest round. We address each reviewer's comments point by point below.

1 Reviewer 1

1.1 Reviewer comment

I really appreciate the extensive changes the authors made to the manuscript. I think they have vastly improved the clarity and strength of the results. There are some remaining concerns I have, and hope these will be helpful to the authors. Please note that all Line numbers here refer to the tracked changes copy of the manuscript.

Author response

Thank you very much for your continued engagement with our paper.

Major Comments

1.2 Reviewer comment

1. The new analysis is much appreciated, but I found the narrative here confusing. A couple points:

It is unclear how the authors reach the current conclusion that "These tests indicate that initial movement vectors start their decay back to baseline from the current state somewhere between moderate and high uncertainty trials." All that is reported are three t-tests between washout and the 3 conditions (which are all significant). It would be helpful to walk the reader through the reasoning here more explicitly.

At least visually it seems that the washout period begins at the level of the "medium" and "high" uncertainty conditions. 10 trials to average the aftereffect causes contamination from forgetting and likely under approximates initial washout state. I would suggest repeating these analyses with smaller numbers of trials: e.g., 1, 2, or 3 for each period, etc. Finally, rather than conducting 3 paired t-tests here, it seems better to run a repeated-measures ANOVA and report on the post-hoc tests (Dunnett's can be used with washout treated as the "control" condition). My suspicion is that using fewer trials for averaging during washout and then correcting for multiple comparisons will show that initial washout is not different from "medium" and "high" uncertainty conditions but is for "low" uncertainty. This may aid in the authors' arguments.

Note that these comments apply for the relevant analyses in Experiments 2 and 3 as well.

Author response

We have made substantial revisions to the reporting of the washout phase results in all three experiments and we feel these changes have helped to further increase the clarity and importance of our findings. These revisions include new phrasing as well as new anlayses. In particular, we now include the suggested repeated-measures ANOVA. However, rather than performing Dunnett's test we have elected to report all pairwise comparisons using paired t-tests corrected for multiple comparisons using the Bonferroni method. We elected to take this approach because we not only care about comparing each uncertainty trial type to washout, but rather we care about how the difference between adaptation and washout differs between uncertainty conditions. That is, we find value and insight in all the pairwise comparisons reported. These pairwise comparisons are now reported in Tables 1, 5, and 9.

1.3 Reviewer comment

2. The new regression analyses help to quantitatively argue that sensory uncertainty makes incremental changes to initial movement vectors (L ¿ M ¿ H ¿ total). This result is convincing now. However, I have confusion around the role that error plays in trial-to-trial learning.

In their regression analyses the authors focus on the effect of uncertainty on learning, and its interaction with error, but not the independent effect of error itself on learning. For this reason, the results are confusing at various places:

(A) The authors discuss at length that the uncertainty terms are significant in their regression model. But they do not mention that in Table 2, both midpoint and endpoint error are statistically significant as well. In fact, the narrative would almost suggest otherwise. For example, on Lines 327-330, the authors state that the results of their regression model that considers change in initial movement vector "echo the results of the regression model built with initial movement vector...". But in the earlier regression, including the log(Trial) factor made the error terms not significant. When this log(Trial) term is removed, and trial-to-trial change is measured, the authors find in Table 2 that both midpoint and endpoint errors are statistically significant. This does not appear to be noted and the narrative confuses on this point a little.

Author response

We have revised our reporting in these sections to provide a more balanced and more complete view of our results. In particular, each of these sections now explicitly addresses whether or not the various error terms are significant and their relative importance to the overall regression. Each of these sections is now summarised with a passage that emphasises that (1) both error and sensory uncertainty influence feedforward adaptation, but (2) they exert this influence independently of each other, and it is this latter finding that contradicts the

classic view that sensory uncertainty scales the error-dependent response.

1.4 Reviewer comment

(B) Along these lines, every plot provided for trial-to-trial change versus error size seems to show a negative relationship within an uncertainty condition across all the experiments. This is why the regression coefficients relating midpoint error and learning tend to be negative across the different experiments (because of these within-condition negative trends). Note that intuitively, we tend to think of larger errors causing more learning, so I might recommend the authors to simply sign-flip the errors in these plots as they will be more intuitive to the reader.

Author response

We thank the reviewer for this suggestion, but feel that this is merely a personal preference and we prefer to keep our results as they are. We are happy to take guidance from the journal editor on this.

1.5 Reviewer comment

(C) I think the authors need to clarify these points in their text, as it is currently either confusing or absent. The lack of significant interactions between uncertainty and error size, but the significant marginal uncertainty and error size factors simply suggest that both uncertainty and error alter learning, but exert independent effects on behavior. I know this is the point the authors are going for, but again, this is somewhat confusing in the way the Results section currently reads. I would suggest that the authors modify this accordingly.

Author response

We are glad that our main point was evident, but agree that our reporting should have been more complete and clearer. As mentioned above, we have revised our reporting in exactly this way.

1.6 Reviewer comment

(D) Finally, I really appreciate the extensive changes the authors have made to their manuscript with regards to their regression models. But I think including both endpoint error and midpoint error in each regression model yields some counter-intuitive results. In particular, in many cases there is a clear negative relationship between trial-to-trial learning and endpoint error for each uncertainty level, but the regression models fail to detect these (e.g., compare Fig. 5c to the positive coefficients in Table 2 for endpoint error; compare Fig. 9c to the positive coefficient for endpoint error in Table 5; and again Fig. 13c with the position coefficient for endpoint error in Table 8). I would recommend that

the authors revisit their decision to include both error terms. At the very least in Exp. 1 (when there is no visual endpoint error), I would recommend running the model solely for midpoint error and removing endpoint error and its interaction terms. For the other experiments, I might also run two separate regression models, one with midpoint errors and the other with endpoint. While I can see an argument for including both terms in a single regression model, separating them would be more consistent with past literature which uses a single error in a state-space model to drive learning, even when feedback is provided continuously. I think this will help to clear up some of these regression results and might also give the regressions more power to detect interactions that may be significant.

Author response

We removed EP and EP interaction terms from experiment 1 and revised the corresponding text, figures, and figure captions (i.e., we removed Fig. 5 panel C). We also added a sentence to the "Statistical modelling" section that reads:

> We omitted the terms for error experienced at endpoint and the corresponding interaction terms in our analysis of Experiment 1 because no endpoint feedback was provided on any trial in this experiment.

We have elected not to report MP-only and EP-only regression models because we feel that our existing approach is (1) more statistically conservative and (2) creates a simpler reading experience. However, we have added text to the captions and associated text describing Figures 5, 9, and 13 to resolve potential confusion in comparing these figures to the statistics tables. In particular, the regression lines in these figures are simple linear regression lines – not derived from the multiple regression reported in the text and tables – and are included only as a convenient visual to aid the perception of trends. This is now more explicitly and clearly stated in the manuscript.

1.7 Reviewer comment

(E) Finally, related to the last point, the authors at many points make the statement that uncertainty exerts an error-independent effect on feedforward adaptation. They explain that low-uncertainty should have lines with the steepest slope in Figs. 5, 9, and 13. While I agree this never appears to be the case for midpoint errors, for endpoint errors in both Experiment 1 and 2 (Fig. 5c and 9c) it does appear that the low-uncertainty condition has a much steeper slope than other conditions. I do wonder whether this might come out in the regression analyses if midpoint and endpoint regressions are done separately. Now, what is interesting here, is that if indeed it is the case that low-uncertainty has the highest slope for endpoint errors, I'm not sure this necessarily means that the response to error is scaled by uncertainty. The reason is that endpoint error = midpoint error + feedback integration, and the authors show that uncertainty impacts feedback integration. So, any interactions between uncertainty and end-

point error size in Figs. 5c or 9c, might actually be due to a feedback integration effect, rather than a true scaling in the error response based on uncertainty. I think the authors may want to touch on these points.

Author response

We thank the reviewer for these insights and we agree with the reasoning behind them. However, we do not feel that these observations are deserving of further treatment in our already very lengthy manuscript. Our position here is related to our decision to not perform separate regressions for midpoint and endpoint feedback.

1.8 Reviewer comment

3. I appreciate very much the authors' taking my suggestions to heart, concerning simpler models that add a context-dependent bias to reaching output. While I do think this is a step in the correct direction, there are some follow-up points I think the authors should consider:

As Reviewer 3 states, the modeling efforts at the moment feel a bit post-hoc. It is not clear what the motivation is for each of the models applied in the paper. With that said, in light of their new results, I have a suggestion. The retention, error, and bias scaling models all seem to me to be in line with the idea that sensory uncertainty may interact with the updating of an adaptive learning process (the authors seem to hint at this in their Discussion). That is, the trial-to-trial change in adapted state depends on forgetting (which would be modulated by the retention-scaling model), error sensitivity (which would be modulated by the error-scaling model), and bias in the state-update process (again recall that while the bias term will ultimately produce a stable bias, in its time-varying state it acts to cause a time-varying trial-to-trial change in the underlying adapted states). Thus, in a sense, these 3 model classes seem to me to embody the possibility that uncertainty will somehow change the trial-to-trial error-based learning process. Therefore, the result that these 3 model classes perform the poorest, is very much in line with the authors' empirical analyses that observe an independence between error-based learning and sensory uncertainty.

The state and output-aim scaling models, however, embody a very different possibility – that sensory uncertainty does not change the way we learn trial-to-trial, but instead differentially activates an additional aiming process with no memory. This would seem to be a class of models that attempt to treat traditional error-based learning processes as independent from the "boosts" in motor output activated by sensory uncertainty. The improved performance of these state-space models seems therefore to be in line with the regression models, which find independence between error-based learning and sensory uncertainty.

I state all this because as it stands, the authors provide no context for the 5

model classes they tested (some is included in Discussion). And coming full circle, this makes the whole modeling effort seem post-hoc and unmotivated. I don't think it needs to be this way, and I think the authors can more clearly delineate very important sub-classes in their models. Namely, that the error, retention, and bias-scaling models all postulate that the states which contribute to the washout aftereffect are modulated trial-to-trial by sensory uncertainty. These are qualitatively similar to the notion that sensory uncertainty should modulate the way we change our behavior in response to an error, as tested in the regression models. On the other hand, the state and output-scaling models, postulate that a memory-free state (i.e., it does not retain its value from one trial to the next) is activated differentially by different uncertainty contexts. In other words, the process of error-based learning is not altered by uncertainty, but rather uncertainty can transiently activate additional memory-free processes that modulate overall behavior. I apologize for the wordiness of these past 3 paragraphs, but to me, I think this could be very important context to provide the reader, and a helpful way to interpret the differential performance between the 5 model classes (ultimately, the state and output-scaling models are in line with the idea that uncertainty transiently activates an explicit aiming state that can be turned on and off). Whether or not the authors agree with the interpretation above, ultimately, I hope these comments help to explain the need for the authors to better contextualize and motivate their state-space modeling efforts in their Results (and then further discuss them in Discussion in more detail).

Author response

We thank the reviewer for this helpful insight and suggestion. We have elected to reframe the models as suggested and we are optimistic that these changes have improved the motivation for and clarity of our modelling.

1.9 Reviewer comment

Now, on to some criticisms and suggestions for improvement in the model:

(A) While I appreciate the authors approach to compare the model evidence via t-tests on BIC, I am not sure this is correct. A better way to compare these models is to for each participant, rank the models from most to least likely (based on BIC for each model). So, for example, in a study with 3 participants and 3 models. Say for Participant 1, the order of models from most to least likely in 1, 2, 3. For Participant 2 it is 1, 3, 2, and for Participant 3, it is 2, 1, 3. What we can then say is that Model 1 is best for 2 participants, and second-best for 1. Model 2 is best for 1 participant, second-best for 1 participant, and worst for 1 participant. Finally, Model 3 is second-based for 1 participant, and worst for 2 participants. The clear ranking emerges that Model 1 explains subject performance for the majority of subjects. For two references that employ this approach to model comparison see both Forano & Franklin (2020, PLoS Comp

Biol), and Orozco et al. (2021, PLoS Comp Biol). The Forano paper is more helpful in the present case, and could be used to construct the heat maps shown for model comparison in their paper. The authors should replace their paired t-test approach with this preferred method.

Author response

We performed the suggested rank-based analysis and agree that it is a nice way to summarise the model-based results. We have elected to leave the t-tests in the manuscript since we feel that it is helpful to have some formal statistics and the reviewer did not outline a reason why we should remove them.

1.10 Reviewer comment

(B) Given the high complexity of the models (nearly 20 parameters in some cases), I would recommend that the authors perform a model recovery analysis. Both the Forano and Orozco papers above show how this can be done. Essentially, "fake" data are simulated with a given model, and then all 5 models are fit to the simulated data as if real participants performed the task. You then calculate the probability that the "true" model is selected by the BIC comparison. Ideally, if Model X is used to simulate the data, then Model X will be recovered by the BIC analysis 100 percent of the time (though this will certainly not be true in practice). If the authors find that for example, a model recovery analysis shows that data simulated by a state aim-scaling model are equally likely to be recovered by a state aim-scaling model and an output aim-scaling model, this would help to explain why these models perform similarly well in their model comparisons (along these lines, if these simulations are performed for Exp. 3, they might find that the aim-scaling models are often recovered by bias-scaling models, which might help to explain the pattern in Fig. 15). It would also suggest that the current experiments are not sufficient to tease apart these two model classes, and this point should be listed as a limitation.

Author response

We thank the reviewer for this suggestion, but since the new framing of our paper no longer relies on parameter estimation from these models to make our main statistical points, we have elected to keep our paper shorter and simpler by not performing the suggested model recovery analysis. We do agree that doing so could be an interesting avenue for future research.

1.11 Reviewer comment

(C) While I appreciate that the authors added models that constrain the model parameters to be "positive", there are a couple things that could be improved. First, it is not meaningful for the authors to compare "complete parameter

space" models to "non-negative constrained" models as they do in their Results. Their result that the complete parameter space model will perform best is necessarily true, as these models are a superset of the non-negative models. That is, for any particular participant, a non-negative parameter model can only do just as well as the complete-parameter model or worse: it can never do better (provided the least-mean squared fitting process truly identifies the global minimum error). Therefore, these analyses should be removed from the Results.

Author response

We have removed these statistics from the manuscript.

1.12 Reviewer comment

(D) Along the lines of my past point, I think the authors can simplify their paper (and reduce supplemental figure count) by doing away with the "full parameter set" models. Again, models with the "negative" parameter sets can produce negative steady-states in the underlying states that are not physiologically meaningful. It does not seem good practice to fit a model to the data whose underlying states cannot be a realistic interpretation of biology. I do not agree with the authors that the two-state model was intended to be completely phenomenological. When this model was proposed by Smith et al. (2006), its primary use was to account for the phenomenon of spontaneous recovery: the idea that multiple states can track a perturbation at different rates, thus giving rise to complicated motor outcomes. The notion that one state would completely fail to correctly track a perturbation's sign (as can occur without non-negative constraints), goes entirely counter to the ethos of what a two-state model is intended to do: produce two states with differential forgetting that track a perturbation's state with different rates. Thus, while I appreciate past discussion (Haith and Krakauer) about the phenomenological nature of the two-state model (i.e., we do not have specific neural correlates for the states), this model is indeed intended to be composed at its core of two states that track a perturbation (not one that tracks a perturbation, and the other that goes off to a unphysiological value). Unless the authors have reason to suspect that an adaptive state that could contribute to learning in this task would reasonably go counter to the rotation's sign, I do not think it correct to permit this behavior. I do not see why it matters to include both types of models, given that the authors state their conclusions are analogous either way (their Results and Figure counts could be simplified).

Author response

We thank the reviewer for the opportunity to consider this issue further. First, we do not think that this is a simple matter with a clear and unanimous consensus. second, one possible criticism of excluding the "full parameter set" models

is that it may leave readers that do not share this reviewers opinion concerned that if the models had been allowed to express themselves over a broader set of parameter values then the results would have changed. As such, we think it is ultimately best to leave both sets of models in the manuscript and let the reader decide whether or not they view one set as physiologically implausible. That said, we are happy to take guidance from the editor on this point.

1.13 Reviewer comment

(E) Finally, I wish to again re-visit the way bias is incorporated in the authors' models. I appreciate the authors' reference to the Cheng and Sabes (2006) paper. But I do not necessarily agree with the notion that placing the bias in the state-update is "preferred over placing the bias on the motor output". Yes, Cheng and Sabes do show that placing the bias on the motor output can cause this bias to be adapted away. But the only time this occurs (see their derivation and exposition) is a case where A (the retention matrix) is the Identity matrix. In other words, the notion that placing bias on the motor output is less preferred that the state update comes only in the case of assuming that retention factors (a) are equal to 1. This, however, is not true, as memory systems exhibit forgetting. Thus, both formulations produce sensorimotor biases for realistic parameter sets. With that said, Cheng and Sabes appear to miss a qualitative structural difference for how these different model formulations will create biases. See the toy simulation below. These are single state models. At left, a bias is placed on the motor output equation (my suggestion last review); at right, a bias is placed on the state-update equation (current paper).

Note that both formulations produce stable and consistent biases such that reaching behaviors are offset from the "0" baseline both prior to adaptation and after washout. Both formulations also have a time-varying period at the start of experiment, over which steady-state bias is reached. But there is a critical qualitative difference between the two. When biases are placed on motor output (left), the system acts to reduce this bias through error-based learning, and so it decreases. When biases are placed on the state-update equation, behavior starts at "0", but then grows into its stable bias. Thus, the magnitude of bias increases to its steady-state for the state-update formulation; and the magnitude of bias decreases (is partially adapted away) in the motor output formulation.

I think the authors need to answer a couple questions to inform their modeling decision:

1. Is there any appreciable bias in reaching behavior? When the authors analyze behavior during their baseline period, or during the washout period, do participant behaviors reach steady-states that are significantly different from 0? If this is not prevalent in many participants, then why include a bias in the model at all?

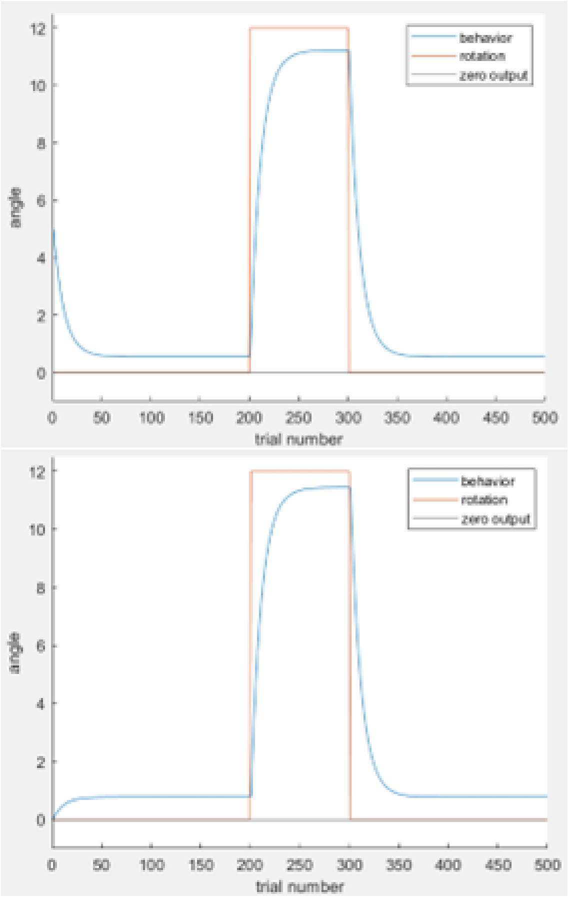

Author response

We thank the reviewer for their detailed comments. The bias-scaling models in our manuscript are not intended to provide a model of bias in baseline reaching behaviour, but rather are intended to capture the idea that uncertainty-dependent scaling may affect a non-error term in the state update. This is captured very eloquently by one of this reviewers previous comments.

> That is, the trial-to-trial change in adapted state depends on... bias in the state-update process (again recall that while the bias term will ultimately produce a stable bias, in its time-varying state it acts to cause a time-varying trial-to-trial change in the underlying adapted states).

This observation is precisely the point of including the bias-scaling model. That is, it is similar to the aim-scaling models, but unlike the aim-scaling models it does not demand that the fast state-update is error-free and zero-retention.

Finally, as is clearly seen by the rank analysis also suggested by this reviewer the bias-scaling model performs third best. For all of these reasons, we think that including the bias-scaling models is justified and strengthens the paper.

1.14 Reviewer comment

2. The authors should analyze their baseline behaviors. Do biases start with a large magnitude (either positive or negative) and then move towards "0". In this case, the motor output formulation should be used. Do biases appear to grow during the baseline period? If so, their current state-update formulation should be used.

Author response

Please see our reasoning for including the bias-scaling model above.

1.15 Reviewer comment

3. Are biases in the data transient? In other words, can motor biases be present during baseline because the way participants handle the KINARM? Might they make structural changes to their arm posture, such that biases are eliminated, and then during the washout period, they would no longer decay to the bias exhibited at baseline? In other words, the bias should be consistent prior to adaptation, and then after adaptation. Otherwise, the models will incorrectly incorporate a bias throughout the entire adaptation process that is actually only transient.

Author response

Please see our reasoning for including the bias-scaling model above.

1.16 Reviewer comment

4. Ultimately, if there is no strong evidence for these biases, I recommend the authors simplify their models and remove the bias process altogether. The current distinction between their model classes (that uncertainty acts on the process of learning, or that uncertainty activates a transient and separate aiming state), can be made by comparison of the retention/error-scaling models to the state/output aim-scaling models, without any need for this tricky business of bias.

Author response

Please see our reasoning for including the bias-scaling model above.

1.17 Reviewer comment

Especially in their "full parameter set" models, I remain concerned that these bias terms can produce unphysiological over-fitting of the data. I think it is really important to get this "bias" question correct, or to remove it entirely, because to my knowledge, two-state modeling work has not used bias terms in the field (excepting the Cheng and Sabes paper from 2006), and the authors' model formulation may set a precedent for the field that needs corroboration.

Author response

We previously addressed this reviewers concerns regarding overfitting, and no new specific points are raised here explaining why our treatment of that problem were unsatisfactory to them. As such, we have not done more than previously described to assess the possibility of overfitting. Here is what we wrote previously:

> All models are fit to individual participants and used to generate predicted performance for each participant. These predictions are then averaged and overlaid with the mean human behaviour in all model prediction figures. The R^2 values reported in our original submission were very high because it was the value obtained by comparing just these two average curves. In our revised manuscript, we report the average of all individual R^2 values, which are indeed smaller.
>
> Though the R^2 values are smaller, and likely agree more with your intuitions, there are further compelling reasons to believe that overfitting is not a problem for our analysis.
>
> First, the model is being fit to the midpoint and endpoint observations across 280 trials. That means that the less than 20 model parameters are being asked to fit 560 data points. We were unable to find any resource on model diagnostics for which this amount of data was not considered much more than sufficient constraint for our situation.
>
> Second, we don't think that fitting unique "blips" is a weakness of our approach, but rather we view it as a strength. That is, the insight should be how suprising it is that across 180 trials of adaptation only 4 parameters are needed to capture what would otherwise seem like wild volatility in performance.
>
> We note here again that the models are fit to individual participants. This is what is so remarkable about the pattern in our data. Every participant across every trial responds to the sensory uncertainty in a very consistent and yet remarkably counter-intuitive way. This is the only reason why only 4 parameters can do so much work.

> Finally, we performed an analysis in which we optimised the model parameters over only a random sample of adaptation trials and computed the model performance on the left out trials. Model performance was slightly worse than when optimised on all data but only negligibly. We have elected to leave this analysis out of the manuscript due to space concerns, but we are happy to add it if the editor feels it is worth the space.

1.18 Minor comments

1.19 Reviewer comment

Lines 192-209 in tracked changes

I'd recommend that where it says "...washout phase (purple dots in Fig. 3)..." that this is changed to "Fig. 3a" because purple appears in the other sub-plots as well.

Author response

Corrected.

1.20 Reviewer comment

The statements "...adaptation estimated from the high uncertainty trials...", or "...adaptation estimated from low uncertainty trials..." is a little ambiguous here. I thought that the colors in Fig. 3a are not for a particular trial type, but for the preceding trial type.

Author response

Updated to:

"aligned with the adaptation estimated by initial movement vectors preceded by medium, high, and infinite uncertainty trials (orange, green, and red dots in Fig. **??**) than it does with the adaptation estimated by initial movement vectors preceded by low uncertainty trials (blue dots in Fig. **??**)."

1.21 Reviewer comment

The authors cite (Line 945) two previous studies that sampled perturbations from a normal distribution. There are at least two more studies that used such a paradigm (Fernandes et al., PLoS One, 2012; Albert et al., Nat Hum Behav, 2021).

Author response

We have since clarified that only two studies have combined trial-by-trial variations in perturbation

1.22 Reviewer comment

Typos:

Line 344: "...integrations as a function or error at midpoint...". Should be "of".

Line 370: "...these results are consistent [with] prior studies...".

Fig. 12: should the factors have subscripts "LL", "LH", etc., instead of solely "L", "M", and "H"?

Type: Line 764: Table "??".

Author response

All typos corrected

2 Reviewer 2

2.1 Reviewer comment

The revision answers some of my previous concerns and, more importantly, provides more experimental and modeling findings. Interestingly, all the reviewers pointed to one most probable explanation of the seemingly surprising data: the abrupt changes in initial movement direction are probably caused by rapid explicit strategy changes. The revision provides supporting evidence that the explicit learning, indirectly shown as the abrupt drop at the beginning of the washout, was predominant for the low-uncertainty condition. This condition showed the surprising abrupt "adaptation" on top of the slow adaptation with other uncertainty conditions. Furthermore, the modeling results also support the role of explicit strategy since the models with abrupt biasing terms (bias scale, state aim, and output aim models) outperform other models. It seems convincing that the main findings can be accounted for by the explicit error correction triggered by the low-uncertainty feedback. However, the authors still interpret the findings as supporting that the presence of feedback integration (aka., the midpoint feedback as opposed to the endpoint feedback that was used in previous studies) alters the effect of uncertainty on feedforward adaptation. I have concerns over the study rationale, the interpretation of the findings, and the way how the study relates to existing literature.

Author response

Many thanks for your continued efforts and patience with this paper.

2.2 Reviewer comment

Major Comments

About the relation between the current study and existing studies. The paper claims it is the first to examine sensory uncertainty when feedforward adaptation and feedback integration co-occur. This is an overstatement. Körding & Wolpert 2004 paper, the seminal paper the current study is based on, arguably combined these two processes. They made people adapt to a lateral shift (adaptation) and, on top of that, used midpoint feedback to probe the "feedback integration" to test within-trial response, just like the current paper did. Furthermore, it is not fair to say that the midpoint feedback is for feedback processes and the endpoint feedback is for feedforward processes. I appreciate that the authors give their definitions of feedback integration and feedforward adaptation in the text, but I respectfully disagree with these terminologies as it creates confusion. Endpoint feedback IS involved in feedback processes and feedback integration; it is just that its effect can only be seen in the next trial for reaching paradigms. Redefining the term will unnecessarily obscure the message of the paper. The authors can call the behavioral measures within-trial correction and cross-trial correction.

Author response

We thank the reviewer for highlighting some potentially confusing aspects in our presentation of previous work. We acknowledge that both feedforward and feedback processes were present in Körding & Wolpert's 2004 paradigm. We have now provided a clearer description of what their study did and did not demonstrate in the discussion section "Key paradigm differences". This section now opens with the following:

> Our study closely resembles the design of Körding and Wolpert (2004) [1], which is one of the seminal studies in establishing error-scaling as a model of sensory uncertainty on feedforward adaptation. However, a crucial distinction lies in our specific focus on the first 180 trials of adaptation, while their study examined behavior after 2000 trials of adaptation had already taken place. This difference in design reflects the divergence in research questions between our two studies. Körding and Wolpert aimed to investigate if participants had learned a prior distribution of a perturbation and whether they would incorporate new information into that prior in a Bayesian manner. Therefore, exposing participants to thousands of trials of the perturbation served as a practical way to impose a prior onto their subjects. They used the interplay between sensory uncertainty

and feedback integration as a readout of what participants believed about the perturbation and how they updated these beliefs. In contrast, our study emphasizes understanding the interplay of feedback integration and feedforward adaptation as participants encounter the perturbation for the first time (e.g., when errors are likely to be relatively large and frequent [2, 3]). In principle, we could have directly compared our results to the early trials in Körding and Wolpert's study. However, regrettably, they did not report this data, leaving us unable to make a direct comparison between our feedforward adaptation results and their findings.

Further, we agree that midpoint feedback is not applied exclusively to feedback processes or that endpoint feedback is applied exclusively to feedforward processes, and we have tried hard to make this point clearly in our manuscript. For example, all state-space models assume that (1) within-trial feedback-integration is driven by the processing of midpoint feedback by the feedback controller and (2) between-trial updates to the feedforward controller are driven by both midpoint and endpoint feedback (see the "State space modelling" section of the methods).

We have also carefully considered the suggestion to revise our terminology and respectfully disagree. This terminological distinction is not unique to us, and is consistent with the broader literature (e.g., Wagner, Mark J., and Maurice A. Smith. 2008. "Shared Internal Models for Feedforward and Feedback Control." The Journal of Neuroscience 28 (42): 10663–73). Our choice of 'feedback integration' instead of the more common 'feedback control' is to intentionally highlight the sensory integration aspect of feedback control, which is central to our study. We considered the more cumbersome 'online sensory integration', but thought it was preferable to use the more compact term. We opted for 'feedforward adaptation' instead of 'feedforward motor adaptation' for similar reasons.

2.3 Reviewer comment

The paper starts with the recognition that sensory uncertainty is believed to slow down adaptation. In the abstract, they mentioned, "Both the degree to which sensory feedback is integrated into an ongoing movement and the degree to which movement errors drive adaptive changes in feedforward motor plans have been shown to scale inversely with sensory uncertainty." However, references 15-22 (quoted on L65 in the Introduction) are not just for showing that sensory uncertainty slows down adaptation. Some quoted studies manipulated the variability of perturbation/error size, targeting uncertainty about perturbation size (or mapping uncertainty in Burge2008's terminology, or environmental/perturbation consistency in Herzfeld2014's terminology) but not about sensory uncertainty. The Kording2004 study showed the inverse scaling for midpoint feedback, which was replicated by the current study (on a side note: this replication by itself runs against the title of the paper, i.e., the error-

independent effect of sensory uncertainty on motor learning. The within-trial response is also part of motor learning; Kording2004 study was based on this idea as they quantified learning by the within-trial response to perturbations). Other quoted studies (Burge2008, Wei2010, Tsay2021) are the ones that manipulated the endpoint sensory uncertainty, and they indeed showed that sensory uncertainty slows adaptation.

Author response

We thank the reviewer for the opportunity to tighten our references to the existing literature. We have removed references to Baddeley et al. (2003), Versteyen et al. (2011) and Fernandes et al. (2012) in the Introduction because their paradigms differ from ours in important ways.

We also thank the reviewer for the opportunity to reflect on our title and definition of motor learning. As we addressed in response to comment 2.2, we believe that our abstract, introduction, and carefully defined terms leave little room for confusion about what we mean by 'motor learning'. While we acknowledge that 'motor learning' is a broad term encompassing various features, including adaptation, we prefer this more general term in the title. We believe it is more accessible to a broader audience compared to the potentially more esoteric term 'feedforward motor adaptation'. Our Introduction aims to ensure any ambiguities related to terminology are effectively resolved.

Finally, we acknowledge that the use of 'motor learning' in our title acts as a placeholder for the myriad features that include adaptation. We consider this approach reasonable and common, and importantly, the main text eliminates any potential ambiguity present in the title.

2.4 Reviewer comment

Thus, the current study only challenges the three papers claiming an inverse scaling of adaptation and endpoint sensory uncertainty. However, as I put above, the experimental findings here appear intriguing, but they can be accounted for by simple or even trivial explanations that are not adequately discussed in the current manuscript. The initial movement direction, aka the feedforward adaptation, exhibited an abrupt increase in learning following low-uncertainty trials (a 4 5 degree increase). The paper repetitively emphasizes that the effect of sensory uncertainty is independent of movement error. Even with the recognition that explicit aiming strategies might underlie these abrupt changes (and I guess we all agree so), the discussion of relevant findings departs from the current theorization of visuomotor rotation adaptation. For example, the observed initial movement vectors are suggested by the authors to reflect "true adapted state of the motor system". However, it is more or less a consensus in the field that the VMR adaptation to large perturbations consists of implicit and explicit processes. I guess the authors are referring to the former as the "true adapted state." If so, please state it clearly, as readers would not understand what the

true adaptation means. Critically, the initial movement vector reflects the sum of implicit and explicit learning; this is the foundation to attribute the abrupt changes following low-uncertainty trials to explicit error correction.

Author response

Thank you for bringing this matter to our attention in a clear and concise manner. We agree that IMVs may be influenced by explicit aiming strategies and therefore may not offer an unbiased or previse estimate of the "true adapted state of the motor system". In the "Adaptation versus aiming" subsection of the Discussion, we used this concept merely to present two rival interpretations of our main results. It was not our intention to endorse this interpretation as correct. We have now revised that section to provide more clarity on our reasoning and avoid any potential misinterpretation.

2.5 Reviewer comment

The other obvious misread of the literature is on L647 where the authors stated that some studies with no apparent link to strategy actually reported a large drop from adaptation to washout and that this finding thus questioned the validity of initial movement vectors as a reliable estimate of the true level of adaptation. First, I don't understand the reasoning here. Second, the quoted studies are mostly old ones before re-aiming strategies are well understood or measured. Third, the washout trial with an exclusion instruction (i.e., excluding the use of strategy) is widely accepted as a reliable measure of implicit learning. This is just another sign that the authors should interpret and discuss the current findings with a clear view of the current understanding of motor adaptation.

Author response

We have removed this section from our discussion.

2.6 Reviewer comment

Going back to the three studies that the current findings appear to contradict. All three of them are probably free from the contamination of explicit strategy. Burge (2008) used a relatively small step change of perturbations (8.2 degrees) in both directions with blurring cursor feedback in their experiment 1. These small angles might make the detection of external perturbations hard (see Oh & Schweighofer 2019), and thus adaptation is mostly driven by slow implicit learning (see their data). Tsay's (2021) study used task-irrelevant error clamps that presumably elicit implicit adaptation only. Wei (2010) study had a zero-mean perturbation size across trials, thus preventing a stable explicit strategy and a slow learning envelope. This is not correctly recognized by the authors (Line 666). Thus, careful examination of the literature and the current findings

should lead us to conclude that explicit strategy use overshadows the effect of sensory uncertainty on adaptation, which are primarily implicit in relevant studies.

Author response

We have since updated our discussion section "Key paradigm differences" to acknowledge these key distinctions across paradigms.

2.7 Reviewer comment

In summary, the study did not fulfill its aims through its experimental design since explicit strategy explains away the major findings. The study aims to study the effect of sensory uncertainty when both feedback integration (midpoint feedback if more precisely called) and feedforward adaptation (i.e., endpoint feedback) are present during visuomotor rotation adaptation. However, having midpoint feedback is not equivalent to independently invoking feedback integration, and having endpoint feedback is not equivalent to independently invoking feedforward adaptation. As I put above, midpoint feedback also drives feedforward adaptation, and endpoint feedback can also be part of the feedback process (it just does not lead to within-trial correction). Thus, the theoretical framework of the study and its actual implementation do not align.

Author response

We feel that this comment reflects several deep confusions about our paper, and we sincerely appreciate the opportunity to improve our writing so that others may more easily benefit from our research.

First, we agree that explicit strategies are a plausible explanation of the major findings but disagree that such an explanation is obvious. The "Adaptation versus aiming" section has been revised to clarify our reasoning. Since the reviewer seems to already favor the explicit strategy explanation, we quote below only our paragraphs from that section that underscore the alternative:

> On the other hand, our proposed explanation for the involvement of explicit aiming in the observed data remains highly speculative. For instance, it is unclear why high uncertainty trials would prompt participants to abandon their previously successful explicit strategy, only to suddenly revert to the same strategy just a few trials later. Additionally, it is puzzling why almost all participants would invoke the same explicit strategy in the same way during these trials. This observation seems inconsistent with explicit aiming, as one would expect humans to devise different coping strategies.
>
> Furthermore, it is important to acknowledge that our study was not designed to distinguish between feedforward adaptation and explicit

> aiming processes. Additionally, all the models we tested yielded relatively high R^2 values, and this suggests that that models that do not resemble explicit aiming can still effectively account for our data. Ultimately, further research is needed to understand the how sensory uncertainty affects the interplay between feedforward adaptation and explicit aiming.

Second, we disagree with the idea that explicit strategies can "explain away" or otherwise trivialize our main findings. Rather, we feel that while they might one day be shown to play an important role in our main findings, this would not in any way undercut the significance of our results. We think that explicit strategies can be an important part of human motor behavior, and understanding how, when, and in what ways they influence other aspects of motor behavior (e.g., feedforward adaptation) is well deserving of scientific coverage.

Finally, we do not equate endpoint feedback to feedforward adaptation and midpoint feedback to feedback-integration. Please see our previous response to comment 2.2 for further elaboration.

2.8 Reviewer comment

More importantly, the blurring of cursor feedback, designed to manipulate sensory uncertainty, leads to undesired consequences, e.g., "touching" the ideal direction or the target, which in turn causes strategic corrections. These strategic corrections dominate the otherwise monotonic learning pattern. On the surface, the current study challenges the Bayesian view of sensory uncertainty in motor adaptation; in reality, it is the experimental specifics that produced some intriguing data.

I suggest the authors correctly quote relevant studies, precisely define their terms, and modestly interpret their findings with the consideration of the current understanding in the field. This means an overhaul of the Introduction and the Discussion, but it would certainly improve the study's contribution to the field.

Author response

We appreciate the reviewer's valuable insights, and the revised manuscript now incorporates the necessary changes to ensure a more accurate representation of the literature, more precise terminology, and a more modest interpretation of our findings within the current understanding of motor adaptation.

2.9 Minor correction

2.10 Reviewer comment

Minor:

Line147: I have no idea why checking these t-tests would indicate the washout

decay is “somewhere between moderate and high uncertainty trials.” This statement is wrong on multiple levels. t statistic and its p values are not for directly showing the effect size. If explicit learning is to be quantified, it is a common approach to compute the difference between the last couple of adaptation trials and the first washout trial, under the condition that participants are instructed to refrain from using the re-aiming strategy before the washout.

Author response

We have completely revised these analyses and our reporting of the corresponding results.

2.11 Reviewer comment

Please correct various writing errors, e.g., L809: Th is; Line285, ?; Line475: Experiment 3.

Author response

Corrected.

2.12 Reviewer comment

Line288: what caveats?

Author response

Revised.

2.13 Reviewer comment

Line640: combine this small paragraph with the one below.

Author response

Paragraphs consolidated.

2.14 Reviewer comment

Line656: high r square means little for showing the validity of the models. The adaptation pattern here is a simple logarithmic function, and the log(trial) can explain most of the data variance. The additional uncertainty bias terms, retention, and learning betas are icing on the cake. Please show the partial eta squares for each term to drive this message home.

Author response

We have added a column indicating the relative importance of each regression term in the summary tables for these results. This relative importance term was computed using the methods of Lindeman RH, Merenda PF, Gold RZ (1980) but see also Groemping et al. 2006 [4, 5]. This is now stated in the "Statistical modelling" section.

References

1 Körding, K. P., & Wolpert, D. M. (2004). Bayesian integration in sensorimotor learning. *Nature*, *427*(6971), 244–247. https://doi.org/10.1038/nature02169

2 Kim, H. E., Morehead, J. R., Parvin, D. E., Moazzezi, R., & Ivry, R. B. (2018). Invariant errors reveal limitations in motor correction rather than constraints on error sensitivity. *Communications Biology*, *1*(1), 1–7
Publisher: Nature Publishing Group.

3 Tsay, J. S., Avraham, G., Kim, H. E., Parvin, D. E., Wang, Z., & Ivry, R. B. (2021). The effect of visual uncertainty on implicit motor adaptation. *Journal of neurophysiology*, *125*(1), 12–22
Publisher: American Physiological Society Bethesda, MD.

4 Lindeman, R. H., Merenda, P. F., & Gold, R. Z. (1980). Introduction to bivariate and multivariate analysis. *(No Title)*.

5 Grömping, U. (2007). Relative importance for linear regression in r: The package relaimpo. *Journal of statistical software*, *17*, 1–27.
