# Supplementary material for: Error-independent effect of sensory uncertainty on motor learning when both feedforward and feedback control processes are engaged
Source: PLoS Comput Biol. 2023 Sep 8;19(9):e1010526. doi: 10.1371/journal.pcbi.1010526 (PMC10522034; doi:10.1371/journal.pcbi.1010526)
Supplement: Attachment — Submitted filename: review3.docx [file pcbi.1010526.s034.docx]

**Summary**

Here the authors test how uncertainty about feedback modulates feedforward adaptation (and also feedback commands). They conduct experiments where participants experience a visuomotor rotation, with visual feedback provided at the reach midpoint and/or endpoint. Their most striking finding (consistent across 3 experiments) is that when trials transition from low uncertainty to high uncertainty, there is a transient increase in adaptation, which is subsequently lost in an “anti-adaptive” fashion. This phenomenon could not be captured with a single-state model, suggesting that this behavior is due to the interplay between at least two states. The authors observe that two states models account for behavior with a gradually developing envelope captured by a slow learning state, and a fast state that has transient increases near low-uncertainty trials, but otherwise, remains close to zero. This behavior is achieved in the fast-state, via a context-dependent scaling in either error-based learning, retention, or state bias. The authors observed that the latter two scaling models best described their data.

**Major concerns**

Overall, I think this paper is very interesting. The effect of zero or low-uncertainty conditions transiently boosting adaptation seems very consistent across Experiments 1-3. However, there are various concerns I have about model behavior, model structure, overfitting, and interpretation. I think the authors could strengthen their paper by addressing these concerns.

**Point 1:** Why are no error bars provided in the primary figures? These should be added.

**Point 2:** I am very confused about the “bias” term that is included in the model. The authors explain (Line 556) that this bias term was intended to “determine the baseline mapping that will be returned to in the absence of sensory input”. But this does not appear to be the case. The bias term, λ, is placed in the state update equation:

*x*(n+1) = β*x*(n) + αδ(n) + λ

As such, λ, behaves as a bias not in the baseline mapping, but in the state-update process. I would be more inclined to describe this as drift. The equilibrium state of this model in the absence of feedback, is not λ. A proper model in which reach angle decays to λ, would have a bias perhaps in the process of motor production, not in the state update process:

*y*(n) = *x*(n) + λ + *r*(n)

Where *x*(n) + λ is the overall motor command, and *r* is the perturbation. This model would indeed decay to *y*(n) = λ, as *x* decays to 0 in the absence of feedback (and perturbation).

As such, with this λ term functioning as a drift in the state-update process, I find it very difficult to interpret the behavior of these models. More importantly, including λ as a drift term in the state update process, would in many circumstances lead to unphysiological behavior. Let us suppose λ = 2 (what is intended to be a 2° bias in reach angle) and that β = 0.99, and α = 0.1. Setting y* to 0 and *r* = 0 (no rotation) yields the following behavior over 100 trials (note, in these simulations, the “scaling” models were not used, solely Eqs. (1)-(3) in the paper).

As you can see, even though there is no perturbation, and the intended goal is to reach to a y* of 0, the model drifts to about 9° (rather than a bias of 2°). Thus, it is as if the participant is adapting to a rotation that does not exist. Similarly, let us re-run this simulation, but after trial 100, remove error entirely (so all that remains is *x*(n+1) = β*x*(n) + λ, because there is no error feedback (treating δ(n) as 0).

Here we can see even more strange model behavior. After the initial steady-state at 9°, removing error-feedback at trial 100 (setting the error term to 0, because it is not observed) causes an acceleration in the drift (because now there is no error-based learning process to counteract the drift). There is no physiological basis for these drifts.

At any rate, I hope these toy simulations are helpful in explaining why I think there is an error in the current computational models. This significantly impedes my ability to interpret model behavior and their comparison. While model parameters are not reported as far as I can see in the paper, I would imagine that the way the models avoid this pathologic behavior, is by estimating λ to be very small. This seems the only way that simulated behavior would not be plagued by the issues above.

The only exception to this rule, is cases where retention factors are 0. For example, in the single state fits in Experiment 1, the authors state that retention parameters were estimated to be 0 (e.g., Figs. 4A-C). When the retention parameter is 0 (not physiological) then indeed, the bias term λ, will simply act to fix the internal state to an unchanging value. But in physiological scenarios where the retention parameter > 0, the bias term λ appearing in the state-update creates issues, as shown in the toy simulations above.

I think these bias terms are currently very problematic in the two-state model simulations, as they appear to lead to a strange behavior in which the fast state decays toward “negative” values (see Point 4 for more details on this). I do not think this unphysiological behavior should be allowed (instead of λ bounded between -10 and 10, it should be positive, if it is assumed the bias is acting on states, as opposed to the overall motor command). On a similar note, could the authors explain the foundation for these bias terms? While I am familiar with the idea that motor execution can be biased (i.e., sometimes a baseline bias in movement is subtracted from data to remove biases in motor commands) this does not seem to be a bias related to the adaptive processes, but rather to the production of movement even when no adaptation has taken place.

**Point 3:** The R^2^ values, shown for example in Figs. 4D-F, for the two-state model fits are very high: higher than I would anticipate (other evidence for this would be the very high ω^2^ reported with ANOVA statistics). This raises the concern of overfitting.

If I understand correctly, a two-state model currently has 2 retention parameters, 2 error sensitivity parameters, a bias term, 4 uncertainty parameters, a temporal discounting parameter, for a total of 10 parameters. This is a lot of free parameters, especially considering that the 4 uncertainty parameters, can exert their influence independently on a trial-by-trial basis (they never all act at the same time, given multiplication by the *I* matrix).

The possibility of overfitting is even stronger for the endpoint hand angle simulations, as seen for example in Figs. 4J-L. Here the R^2^ values are nearly 1. This seems unreasonably high to me and is highly suggestive that the high parameter count / model complexity is overfitting noise in the data.

On this note, while fits are provided to the average timecourse, trial-to-trial noise in movement execution/process noise, will still be present even at n=20. Given the random nature of these Gaussian noise sources, it should not be possible to attain R^2^ near 1, unless the data are overfit.

One way to check whether overfitting is indeed an issue, would be to fit models to individual participants and then calculate/report the R^2^ statistic. For individual participants, trial-to-trial noise should be a dominant feature in the data. R^2^ values for individual participants should be low. If the R^2^ values are high even at the level of fitting individual participants, this again would show that there is significant overfitting of the data.

While I am not sure, I suspect that a major contributor to overfitting is the nature of the model, with the 4 uncertainty terms. These terms will exert a strong trial-by-trial effect, that is independent for each of the 4 uncertainty trial types. I think this way of modeling uncertainty effects gives too much power to the models to fit unique “blips” in the data (be they positive or negative spikes) that can be significantly influenced by noise.

**Point 4:** There appears to be something amiss in model behavior: the “negative” steady-state “equilibria” seen in the fast state in the 2-state model fits, despite perturbations having a “positive” mean value.

In the original two-state model paper by Smith et al., 2006, the fast state does indeed achieve “negative” values, but this is only in the case of a spontaneous recovery experiment, where the perturbation switches to a negative value, and the fast state rapidly approaches that value, thus becoming negative. No such condition occurs in the present study, with the perturbations being on average “positive”, and then entirely removed with no error during the washout period.

The idea that there is a fast state that attains a negative bias, despite the fact that the perturbation is “positive” and remains at this negative bias even during the washout phase seems to me highly suspect and unphysiological. This strange behavior is exemplified in Figs. 6F/8F. Here the slow state obtains a value that is larger than the overall behavior, and the fast state obtains a negative value, and ultimately a bias of about -10 deg during the washout phase. I have never seen this behavior reported in a two-state model, and this seems to me to be a function of unphysiological models overfitting the data.

I think this behavior is the symptom of at least two concerns, that I have noted: (1) that the data are likely being overfit, and (2) that the bias term λ, can produce unphysiological behavior when included in the state update term. Given the high model complexity I cannot be sure, but my guess is that these negative steady-states, are due to bias terms becoming negative. I would not consider these fast states becoming negative as a “feature” of the models used in this paper, but rather a “bug” that should be carefully examined / prevented.

**Point 5:** Model parameters should be reported somewhere, at the very least in a supplemental item.

**Point 6:** The data provided in Fig. 3 is informative, and the text on Lines 133-144 is easy to follow. While it does appear visually that sensory uncertainty has a strong effect on feedforward adaptation, and perturbation size less clear, these claims should be validated quantitatively through some empirical approach. I would recommend calculating trial-by-trial change in initial movement vector. I would then compare these trial-by-trial changes across bins for each uncertainty level. Trial-by-trial change in angle could also be plotted against perturbation size in a continuous fashion.

Two notes here, however. First, it is not the case that perturbation size should drive differences in trial-by-trial adaptation (as implied in Fig. 3B). Rather, it is error that drives adaptation. And so, I would suggest the authors calculate trial-by-trial error. They could then calculate trial-by-trial change in angle against error size. To depict this visually, they could bin this relationship based on error size.

The second note is that it seems these claims could be tested statistically. One issue is that comparing trial-by-trial changes in angle based on perturbation/error size, will be confounded by changes in sensory uncertainty (which clearly, at least in the 0 uncertainty condition, has a strong effect on behavior). Thus, I might suggest the authors perform some sort of multivariate regression, the outcome being trial-to-trial change in reach angle, and the independent variables being (1) error/perturbation size and (2) sensory uncertainty. They should look to see whether error/perturbation size, and/or sensory uncertainty have a statistically significant effect on trial-by-trial change in reach angle.

Similar to the point above, empirical analysis could also be done to support the claims made on Lines 145-155. Here the authors describe an anti-adaptive trend that happens when trial uncertainty increases between two trials. The authors can calculate the trial-by-trial change in reach angle, and bin it based on each transition in uncertainty level. Again, it seems that these responses could then be compared statistically.

The authors should also consider that this is not about a transition in uncertainty per se, but simply the previous trial’s uncertainty. For example, in Fig. 3A, on approximately trial 80, there is a sequence of 3 trials that have zero-uncertainty, all which lie in the “higher” adaptation envelope. This seems to suggest that it is really only the last trial’s uncertainty that leads to the upper/lower envelope, not a transition between uncertainty levels per se.

All in all, it would seem to me that there is additional empirical analysis of the data that the authors could conduct to better explore their data and clarify their model results. Wherever possible, conclusions about behavior should be drawn from an empirical analysis of the data, as opposed to simulations.

**Point 7:** I think the discussion about implicit and explicit systems largely explaining the current results is relatively spot on (see Comment 8). I agree that these experiments are not designed to really test implicit and explicit learning. But if there is a strong suspicion that an interplay between implicit and explicit learning is the root cause of central phenomena in the current data, it seems incumbent upon the authors to evaluate this possibility, with another experiment if need be.

Observations that suggest an explicit process:

A. Consider the behaviors in Figs. 3A, 5A, and 7A. Here, no uncertainty trials (either in midpoint, or endpoint feedback) show a very strong transient boost in adaptation. I think this is highly suspicious (as acknowledged by the authors) for an explicit process. When feedback is unambiguous, participants engage a strategy on the next trial to counter the error. When feedback is uncertain, this explicit process is not used on the next trial. This to me seems a much more reasonable explanation for the “anti-adaptive” behavior.
B. Note that sometimes no feedback trials produce the transient increase in learning in a sporadic manner (this can be seen with the “bumps” upward in Fig. 3A and 5A). While I do not have a clear explanation why this should be the case, it would appear that participants do occasionally choose large aiming angles after a no feedback trial. This transient increase after no error at all, seems to indicate the activity of an intentional process that can be turned on and off (on a side note, by analyzing their data further in an empirical fashion, they can detect how specific “jumps” to the higher envelope are to low-uncertainty conditions).
C. The authors state that during washout, participants were not given feedback, and were instructed to reach straight to the target. These are conditions that are used to assess implicit learning. Interestingly, in all 3 experiments, it appears that there is a discrete jump to the “lower adaptation envelope” at the start of each of these washout periods. This would seem to suggest that the lower adaptation envelope is capturing an implicit process that is revealed during the washout period (via instructions). The “jump” to the upper transient envelope that is abruptly “turned off” during washout, would be exactly consistent with an explicit, intentional process.
D. The models roughly converge to the idea that the primary response to uncertainty is mediated by two qualitatively different states. The slow state accomplishes the lower adaptation envelope. The fast state accomplishes the transient “boosts” in the data to the higher envelope. With the interpretation that the slow state largely captures implicit learning, and the fast state largely captures explicit processes (see McDougle et al., 2015), this again would be consistent with the idea that the apparent anti-adaptive behavior, is simply due to a fast explicit state that switches between two levels based on context.

I think that the possibility that the results are highly influenced by implicit/explicit processes is really critical, as it would completely change the authors’ classification of low to high uncertainty trials as “anti-adaptive”. Rather than the strange transient increases following zero-uncertainty trials be described as anti-adaptive, these could be ascribed to an intentional explicit process that responds in a context-dependent manner to uncertainty level. This would therefore not call for an overhaul of how error-based learning is typically modeled, but rather the inclusion of previous unknown context-dependent effects for the explicit system.

It seems to me that the authors really should attempt to rule out this implicit/explicit hypothesis with additional analysis, or ideally with an additional experiment. There are many that could be performed, but one simple control would be a repeat of Exp. 1, but with an instruction to reach straight to the target after some zero-uncertainty trials. In other words, the participants should be introduced to the idea about reaching straight to the target without feedback. On some trials following a zero-uncertainty trial, participants should be told not to aim, but to move straight to the target. On other trials, this should not be done. If there is a sensitivity to instruction in the response, this would indicate that explicit processes are at play in the current data. If indeed this is done, I might suggest to the authors to not simply do one aftereffect effect trial at a time, but 2 or 3, to make sure that participants do indeed listen to the instructions to stop aiming.

**Point 8:** I really like the Discussion in the “Adaptation versus aiming” section and am inclined to mostly agree here (see Point 7 above). There is however, one statement I take some issue with, and this is the notion of a clear distinction between SPEs driving implicit learning, and performance errors driving explicit strategy. This indeed has been a strongly held belief in the field, but many authors now have observed that performance errors play a strong role in driving both types of learning (see for example Miyamoto et al., 2020 Nat Neuro, Albert et al., 2022 eLife, Ranjan and Smith, 2020, Advances in Motor Learning and Motor Control).

I don’t follow the idea that an SPE (which is midpoint or endpoint) that drives implicit learning, and a performance error (endpoint) that drives explicit learning, could account for their results. Experiments 1-3 are similar in overall subject behavioral trends, but in Experiment 1, only midpoint feedback is given. Here, the implicit and explicit systems would have to learn from the same error source (the midpoint error). Thus, I’m not sure this is about implicit/explicit adapting to different error sources, but rather the system responding to the same error source in qualitatively different ways.

In any case, it should be noted that SPE versus performance errors are generally not distinguishable. There are only 2 studies which really clearly disentangled SPEs and performance error: Mazzoni and Krakauer, 2006 and Taylor and Ivry, 2011. The latter study clarifies the former and shows that visual target errors (which would not be considered an SPE per se) strongly modify the implicit response, again casting significant doubt that performance/target related phenomena do not modulate implicit learning.

All in all, I do not see why the assumption that implicit and explicit learning from separate error sources is needed to explain the data. On a general level, the notion that the slow envelope is implicit, and the fast and fleeting uncertainty-tuned corrections are explicit, does not seem to rely on these assumptions.

**Point 9:** One thing I wonder is whether the authors should include different classes of models. Here, uncertainty effects on feedforward adaptation are achieved by a context-dependent scaling of either state-update bias, error-based learning, or state retention. I think the authors should add other model types, that achieve uncertainty effects in a different way: e.g., an additive process. For example, a model could look something like:

*x_s_*(n+1) = β_s_*x_s_*(n) + α*_s_*δ(n)

*x_f_*(n+1) = β_f_*x_f_*(n) + α*_f_*δ(n) + ε(n)

Here ε is a context-dependent term that assumes different values depending on uncertainty. Even simpler models than this could be included in the model comparison, one in which the fast state behaves like an intentional explicit system, and simply turns on/off to 1 of 3 values depending on uncertainty:

*x_f_*(n+1) = ε(n)

Here *x_f_* will simply be set to 1 of 3 values, presumably 0 when trial *n* is no feedback or high uncertainty (no explicit process is used after this), a medium value for a low-uncertainty trial, and a high value for a zero-uncertainty trial.

I think trying a model like this will reduce model complexity (and thus overfitting), while potentially capturing the main features of the data. My secondary hope is that additive models (in which the issue in the bias term noted in Point 2 is fixed) will not show the pathologic “negative” fast state behavior noted in Point 4. Overall, I think the authors could try ways to reduce their model complexity, while still capturing the main features in the data.

**Minor concerns**

**Point 10:** Be careful with language on Line 148-149. Yes, on average trial-by-trial changes should be in the positive direction. But even if uncertainty level never changed, there is motor/process noise that would cause the adaptation level to not increase monotonically on each trial. Secondly, when participants reach asymptotic behavior (steady-state), then on average there should be an equal number of trials where reach angle increases and reach angle decreases.

**Point 11:** Be careful with language on Lines 193-194. Yes, it is the case that the state and bias scaling models provide a better fit to the data than the error-scaling model. But that does not necessarily mean that feedforward adaptation is not well captured by the error-scaling model. Note again that the R^2^ values, e.g., R^2^=0.84 in Fig. 4D, are exceptionally high, and indicate that each model provides an excellent fit. It is really more a function here of choosing between 3 models that provide extraordinarily good fits to behavior.

**Point 12:** I’m not exactly sure where the conclusion is coming from in Lines 206-208, that feedback uncertainty does not inversely scale the magnitude of error-driven feedforward update. Is this related to the comment on Lines 193-194, that the error-scaling model does not do as well at fitting the data? If so, again I would be very cautious here, because all the models provide excellent fits to behavior. Because the error-scaling model isn’t as good as the other models, should not be taken in a strong form to mean that Exp. 1 disproves the idea that error uncertainty does not inversely scale trial-by-trial error-based learning magnitudes. It seems this claim could be evaluated empirically in the data (see Point 6 above).

**Point 13:** I’m not following the narrative on Lines 213-216, where the authors bring up “temporal proximity of the sensory feedback signal to movement offset”. My confusion arises because the authors bring this up as what appears to be an alternate hypothesis for their results in Exp. 1. But all participants and all trials had feedback only at movement midpoint, and the effect of temporal proximity of feedback to movement offset was not tested yet. So, I am a little confused where this line of thought/exposition is coming from.

**Point 14:** Typo on Line 393: the experiment number is not indicated here.

**Point 15:** Low uncertainty (orange) trials seem to cause a moderate increase in adapted reach angle on the next trial (see Figs. 3A and 5A). This may have two causes. A true intermediate boost in adaptation. Or a large, but improbable boost in adaptation. To explain what I mean, it could be that on no-uncertainty trials, adaptation boosts by 3 degrees on the next trial only, 100% of the time. But on low-uncertainty trials, adaptation boosts by 3 degrees on the next trial, but only 20% of the time. The rest of the time there is little to no boost. Alternatively, low-uncertainty trials might boost adaptation by 0.6 degrees, 100% of the time. I would be interested in the authors examining the histogram of the trial-by-trial change in reach angle on the low-uncertainty and zero-uncertainty trials, to arbitrate between these possibilities.
