# Supplementary material for: Error-independent effect of sensory uncertainty on motor learning when both feedforward and feedback control processes are engaged
Source: PLoS Comput Biol. 2023 Sep 8;19(9):e1010526. doi: 10.1371/journal.pcbi.1010526 (PMC10522034; doi:10.1371/journal.pcbi.1010526)
Supplement: Attachment — Submitted filename: review3.docx [file pcbi.1010526.s036.docx]

I really appreciate the extensive changes the authors made to the manuscript. I think they have vastly improved the clarity and strength of the results. There are some remaining concerns I have, and hope these will be helpful to the authors. Please note that all Line numbers here refer to the tracked changes copy of the manuscript.

Major comments

1. The new analysis is much appreciated, but I found the narrative here confusing. A couple points:

- It is unclear how the authors reach the current conclusion that “These tests indicate that initial movement vectors start their decay back to baseline from the current state somewhere between moderate and high uncertainty trials.” All that is reported are three t-tests between washout and the 3 conditions (which are all significant). It would be helpful to walk the reader through the reasoning here more explicitly.
- At least visually it seems that the washout period begins at the level of the “medium” and “high” uncertainty conditions. 10 trials to average the aftereffect causes contamination from forgetting and likely underapproximates initial washout state. I would suggest repeating these analyses with smaller numbers of trials: e.g., 1, 2, or 3 for each period, etc. Finally, rather than conducting 3 paired t-tests here, it seems better to run a repeated-measures ANOVA and report on the post-hoc tests (Dunnett’s can be used with washout treated as the “control” condition). My suspicion is that using fewer trials for averaging during washout and then correcting for multiple comparisons will show that initial washout is not different from “medium” and “high” uncertainty conditions but is for “low” uncertainty. This may aid in the authors’ arguments.
- Note that these comments apply for the relevant analyses in Experiments 2 and 3 as well.

2. The new regression analyses help to quantitatively argue that sensory uncertainty makes incremental changes to initial movement vectors (L > M > H > total). This result is convincing now. However, I have confusion around the role that error plays in trial-to-trial learning.

In their regression analyses the authors focus on the effect of uncertainty on learning, and its interaction with error, but not the independent effect of error itself on learning. For this reason, the results are confusing at various places:

- The authors discuss at length that the uncertainty terms are significant in their regression model. But they do not mention that in Table 2, both midpoint and endpoint error are statistically significant as well. In fact, the narrative would almost suggest otherwise. For example, on Lines 327-330, the authors state that the results of their regression model that considers change in initial movement vector “echo the results of the regression model built with initial movement vector…”. But in the earlier regression, including the log(Trial) factor made the error terms not significant. When this log(Trial) term is removed, and trial-to-trial change is measured, the authors find in Table 2 that both midpoint and endpoint errors are statistically significant. This does not appear to be noted and the narrative confuses on this point a little.
- Along these lines, every plot provided for trial-to-trial change versus error size seems to show a negative relationship within an uncertainty condition across all the experiments. This is why the regression coefficients relating midpoint error and learning tend to be negative across the different experiments (because of these within-condition negative trends). Note that intuitively, we tend to think of larger errors causing more learning, so I might recommend the authors to simply sign-flip the errors in these plots as they will be more intuitive to the reader.
- I think the authors need to clarify these points in their text, as it is currently either confusing or absent. The lack of significant interactions between uncertainty and error size, but the significant marginal uncertainty and error size factors simply suggest that both uncertainty and error alter learning, but exert independent effects on behavior. I know this is the point the authors are going for, but again, this is somewhat confusing in the way the Results section currently reads. I would suggest that the authors modify this accordingly.
- Finally, I really appreciate the extensive changes the authors have made to their manuscript with regards to their regression models. But I think including both endpoint error and midpoint error in each regression model yields some counterintuitive results. In particular, in many cases there is a clear negative relationship between trial-to-trial learning and endpoint error for each uncertainty level, but the regression models fail to detect these (e.g., compare Fig. 5c to the positive coefficients in Table 2 for endpoint error; compare Fig. 9c to the positive coefficient for endpoint error in Table 5; and again Fig. 13c with the position coefficient for endpoint error in Table 8). I would recommend that the authors revisit their decision to include both error terms. At the very least in Exp. 1 (when there is no visual endpoint error), I would recommend running the model solely for midpoint error and removing endpoint error and its interaction terms. For the other experiments, I might also run two separate regression models, one with midpoint errors and the other with endpoint. While I can see an argument for including both terms in a single regression model, separating them would be more consistent with past literature which uses a single error in a state-space model to drive learning, even when feedback is provided continuously. I think this will help to clear up some of these regression results and might also give the regressions more power to detect interactions that may be significant.
- Finally, related to the last point, the authors at many points make the statement that uncertainty exerts an error-independent effect on feedforward adaptation. They explain that low-uncertainty should have lines with the steepest slope in Figs. 5, 9, and 13. While I agree this never appears to be the case for midpoint errors, for endpoint errors in both Experiment 1 and 2 (Fig. 5c and 9c) it does appear that the low-uncertainty condition has a much steeper slope than other conditions. I do wonder whether this might come out in the regression analyses if midpoint and endpoint regressions are done separately. Now, what is interesting here, is that if indeed it is the case that low-uncertainty has the highest slope for endpoint errors, I’m not sure this necessarily means that the response to error is scaled by uncertainty. The reason is that endpoint error = midpoint error + feedback integration, and the authors show that uncertainty impacts feedback integration. So, any interactions between uncertainty and endpoint error size in Figs. 5c or 9c, might actually be due to a feedback integration effect, rather than a true scaling in the error response based on uncertainty. I think the authors may want to touch on these points.

3. I appreciate very much the authors’ taking my suggestions to heart, concerning simpler models that add a context-dependent bias to reaching output. While I do think this is a step in the correct direction, there are some follow-up points I think the authors should consider.

As Reviewer 3 states, the modeling efforts at the moment feel a bit post-hoc. It is not clear what the motivation is for each of the models applied in the paper. With that said, in light of their new results, I have a suggestion. The retention, error, and bias scaling models all seem to me to be in line with the idea that sensory uncertainty may interact with the updating of an adaptive learning process (the authors seem to hint at this in their Discussion). That is, the trial-to-trial change in adapted state depends on forgetting (which would be modulated by the retention-scaling model), error sensitivity (which would be modulated by the error-scaling model), and bias in the state-update process (again recall that while the bias term will ultimately produce a stable bias, in its time-varying state it acts to cause a time-varying trial-to-trial change in the underlying adapted states). Thus, in a sense, these 3 model classes seem to me to embody the possibility that uncertainty will somehow change the trial-to-trial error-based learning process. Therefore, the result that these 3 model classes perform the poorest, is very much in line with the authors’ empirical analyses that observe an independence between error-based learning and sensory uncertainty.

The state and output-aim scaling models, however, embody a very different possibility – that sensory uncertainty does not change the way we learn trial-to-trial, but instead differentially activates an additional aiming process with no memory. This would seem to be a class of models that attempt to treat traditional error-based learning processes as independent from the “boosts” in motor output activated by sensory uncertainty. The improved performance of these state-space models seems therefore to be in line with the regression models, which find independence between error-based learning and sensory uncertainty.

I state all this because as it stands, the authors provide no context for the 5 model classes they tested (some is included in Discussion). And coming full circle, this makes the whole modeling effort seem post-hoc and unmotivated. I don’t think it needs to be this way, and I think the authors can more clearly delineate very important sub-classes in their models. Namely, that the error, retention, and bias-scaling models all postulate that the states which contribute to the washout aftereffect are modulated trial-to-trial by sensory uncertainty. These are qualitatively similar to the notion that sensory uncertainty should modulate the way we change our behavior in response to an error, as tested in the regression models. On the other hand, the state and output-scaling models, postulate that a memory-free state (i.e., it does not retain its value from one trial to the next) is activated differentially by different uncertainty contexts. In other words, the process of error-based learning is not altered by uncertainty, but rather uncertainty can transiently activate additional memory-free processes that modulate overall behavior. I apologize for the wordiness of these past 3 paragraphs, but to me, I think this could be very important context to provide the reader, and a helpful way to interpret the differential performance between the 5 model classes (ultimately, the state and output-scaling models are in line with the idea that uncertainty transiently activates an explicit aiming state that can be turned on and off). Whether or not the authors agree with the interpretation above, ultimately, I hope these comments help to explain the need for the authors to better contextualize and motivate their state-space modeling efforts in their Results (and then further discuss them in Discussion in more detail).

Now, on to some criticisms and suggestions for improvement in the model:

- While I appreciate the authors approach to compare the model evidence via t-tests on BIC, I am not sure this is correct. A better way to compare these models is to for each participant, rank the models from most to least likely (based on BIC for each model). So, for example, in a study with 3 participants and 3 models. Say for Participant 1, the order of models from most to least likely in 1, 2, 3. For Participant 2 it is 1, 3, 2, and for Participant 3, it is 2, 1, 3. What we can then say is that Model 1 is best for 2 participants, and second-best for 1. Model 2 is best for 1 participant, second-best for 1 participant, and worst for 1 participant. Finally, Model 3 is second-based for 1 participant, and worst for 2 participants. The clear ranking emerges that Model 1 explains subject performance for the majority of subjects. For two references that employ this approach to model comparison see both Forano & Franklin (2020, PLoS Comp Biol), and Orozco et al. (2021, PLoS Comp Biol). The Forano paper is more helpful in the present case, and could be used to construct the heat maps shown for model comparison in their paper. The authors should replace their paired t-test approach with this preferred method.
- Given the high complexity of the models (nearly 20 parameters in some cases), I would recommend that the authors perform a model recovery analysis. Both the Forano and Orozco papers above show how this can be done. Essentially, “fake” data are simulated with a given model, and then all 5 models are fit to the simulated data as if real participants performed the task. You then calculate the probability that the “true” model is selected by the BIC comparison. Ideally, if Model X is used to simulate the data, then Model X will be recovered by the BIC analysis 100% of the time (though this will certainly not be true in practice). If the authors find that for example, a model recovery analysis shows that data simulated by a state aim-scaling model are equally likely to be recovered by a state aim-scaling model and an output aim-scaling model, this would help to explain why these models perform similarly well in their model comparisons (along these lines, if these simulations are performed for Exp. 3, they might find that the aim-scaling models are often recovered by bias-scaling models, which might help to explain the pattern in Fig. 15). It would also suggest that the current experiments are not sufficient to tease apart these two model classes, and this point should be listed as a limitation.
- While I appreciate that the authors added models that constrain the model parameters to be “positive”, there are a couple things that could be improved. First, it is not meaningful for the authors to compare “complete parameter space” models to “non-negative constrained” models as they do in their Results. Their result that the complete parameter space model will perform best is necessarily true, as these models are a superset of the non-negative models. That is, for any particular participant, a non-negative parameter model can only do just as well as the complete-parameter model or worse: it can never do better (provided the least-mean squared fitting process truly identifies the global minimum error). Therefore, these analyses should be removed from the Results.
- Along the lines of my past point, I think the authors can simplify their paper (and reduce supplemental figure count) by doing away with the “full parameter set” models. Again, models with the “negative” parameter sets can produce negative steady-states in the underlying states that are not physiologically meaningful. It does not seem good practice to fit a model to the data whose underlying states cannot be a realistic interpretation of biology. I do not agree with the authors that the two-state model was intended to be completely phenomenological. When this model was proposed by Smith et al. (2006), its primary use was to account for the phenomenon of spontaneous recovery: the idea that multiple states can track a perturbation at different rates, thus giving rise to complicated motor outcomes. The notion that one state would completely fail to correctly track a perturbation’s sign (as can occur without non-negative constraints), goes entirely counter to the ethos of what a two-state model is intended to do: produce two states with differential forgetting that track a perturbation’s state with different rates. Thus, while I appreciate past discussion (Haith and Krakauer) about the phenomenological nature of the two-state model (i.e., we do not have specific neural correlates for the states), this model is indeed intended to be composed at its core of two states that track a perturbation (not one that tracks a perturbation, and the other that goes off to a unphysiological value). Unless the authors have reason to suspect that an adaptive state that could contribute to learning in this task would reasonably go counter to the rotation’s sign, I do not think it correct to permit this behavior. I do not see why it matters to include both types of models, given that the authors state their conclusions are analogous either way (their Results and Figure counts could be simplified).
- Finally, I wish to again re-visit the way bias is incorporated in the authors’ models. I appreciate the authors’ reference to the Cheng and Sabes (2006) paper. But I do not necessarily agree with the notion that placing the bias in the state-update is “preferred over placing the bias on the motor output”. Yes, Cheng and Sabes do show that placing the bias on the motor output can cause this bias to be adapted away. But the only time this occurs (see their derivation and exposition) is a case where *A* (the retention matrix) is the Identity matrix. In other words, the notion that placing bias on the motor output is less preferred that the state update comes only in the case of assuming that retention factors (*a*) are equal to 1. This, however, is not true, as memory systems exhibit forgetting. Thus, both formulations produce sensorimotor biases for realistic parameter sets. With that said, Cheng and Sabes appear to miss a qualitative structural difference for how these different model formulations will create biases. See the toy simulation below. These are single state models. At left, a bias is placed on the motor output equation (my suggestion last review); at right, a bias is placed on the state-update equation (current paper).

Note that both formulations produce stable and consistent biases such that reaching behaviors are offset from the “0” baseline both prior to adaptation and after washout. Both formulations also have a time-varying period at the start of experiment, over which steady-state bias is reached. But there is a critical qualitative difference between the two. When biases are placed on motor output (left), the system acts to reduce this bias through error-based learning, and so it decreases. When biases are placed on the state-update equation, behavior starts at “0”, but then grows into its stable bias. Thus, the magnitude of bias increases to its steady-state for the state-update formulation; and the magnitude of bias decreases (is partially adapted away) in the motor output formulation.

I think the authors need to answer a couple questions to inform their modeling decision.

1. Is there any appreciable bias in reaching behavior? When the authors analyze behavior during their baseline period, or during the washout period, do participant behaviors reach steady-states that are significantly different from 0? If this is not prevalent in many participants, then why include a bias in the model at all?
2. The authors should analyze their baseline behaviors. Do biases start with a large magnitude (either positive or negative) and then move towards “0”. In this case, the motor output formulation should be used. Do biases appear to grow during the baseline period? If so, their current state-update formulation should be used.
3. Are biases in the data transient? In other words, can motor biases be present during baseline because the way participants handle the KINARM? Might they make structural changes to their arm posture, such that biases are eliminated, and then during the washout period, they would no longer decay to the bias exhibited at baseline? In other words, the bias should be consistent prior to adaptation, and then after adaptation. Otherwise, the models will incorrectly incorporate a bias throughout the entire adaptation process that is actually only transient.
4. Ultimately, if there is no strong evidence for these biases, I recommend the authors simplify their models and remove the bias process altogether. The current distinction between their model classes (that uncertainty acts on the process of learning, or that uncertainty activates a transient and separate aiming state), can be made by comparison of the retention/error-scaling models to the state/output aim-scaling models, without any need for this tricky business of bias. Especially in their “full parameter set” models, I remain concerned that these bias terms can produce unphysiological over-fitting of the data. I think it is really important to get this “bias” question correct, or to remove it entirely, because to my knowledge, two-state modeling work has not used bias terms in the field (excepting the Cheng and Sabes paper from 2006), and the authors’ model formulation may set a precedent for the field that needs corroboration.

Minor

Lines 192-209 in tracked changes

- I’d recommend that where it says “…washout phase (purple dots in Fig. 3)…” that this is changed to “Fig. 3a” because purple appears in the other sub-plots as well.
- The statements “…adaptation estimated from the high uncertainty trials…”, or “…adaptation estimated from low uncertainty trials…” is a little ambiguous here. I thought that the colors in Fig. 3a are not for a particular trial type, but for the preceding trial type.

The authors cite (Line 945) two previous studies that sampled perturbations from a normal distribution. There are at least two more studies that used such a paradigm (Fernandes et al., PLoS One, 2012; Albert et al., Nat Hum Behav, 2021).

Typos:

Line 344: “…integrations as a function or error at midpoint…”. Should be “of”.

Line 370: “…these results are consistent [with] prior studies…”.

Fig. 12: should the factors have subscripts “LL”, “LH”, etc., instead of solely “L”, “M”, and “H”?

Type: Line 764: Table “??”.
